# Differences of Behavioral and Psychological Symptoms of Dementia in Disease Severity in Four Major Dementias

**DOI:** 10.1371/journal.pone.0161092

**Published:** 2016-08-18

**Authors:** Hiroaki Kazui, Kenji Yoshiyama, Hideki Kanemoto, Yukiko Suzuki, Shunsuke Sato, Mamoru Hashimoto, Manabu Ikeda, Hibiki Tanaka, Yutaka Hatada, Masateru Matsushita, Yoshiyuki Nishio, Etsuro Mori, Satoshi Tanimukai, Kenjiro Komori, Taku Yoshida, Hideaki Shimizu, Teruhisa Matsumoto, Takaaki Mori, Tetsuo Kashibayashi, Kazumasa Yokoyama, Tatsuo Shimomura, Yasunobu Kabeshita, Hiroyoshi Adachi, Toshihisa Tanaka

**Affiliations:** 1 Department of Psychiatry, Osaka University Graduate School of Medicine, Suita, Osaka, Japan; 2 Department of Neuropsychiatry, Faculty of Life Sciences, Kumamoto University, Kumamoto, Kumamoto, Japan; 3 Department of Behavioral Neurology and Cognitive Neurosciences, Tohoku University School of Medicine, Sendai, Miyagi, Japan; 4 Department of Neuropsychiatry, Ehime University Graduate School of Medicine, Toon, Ehime, Japan; 5 Comprehensive community care for elderly, nursing and Health science, Graduate School of Medicine, Ehime University, Toon, Ehime, Japan; 6 Zaidan-Niihama Hospital, Niihama, Ehime, Japan; 7 Departments of Neurology and Cognitive disorders, Hyogo Prefectural Rehabilitation Center at Nishi-harima, Tatsuno, Hyogo, Japan; 8 Department of Dementia Research, Akita Prefectural Center for Rehabilitation and Psychiatric Medicine, Daisen, Akita, Japan; 9 The Sleep Medical Center of Osaka University Hospital, Suita, Osaka, Japan; 10 Department of Psychiatry, Osaka University Health Care Center, Toyonaka, Osaka, Japan; Taipei Veterans General Hospital, TAIWAN

## Abstract

**Background/Aims:**

Behavioral and psychological symptoms of dementia (BPSDs) negatively impact the prognosis of dementia patients and increase caregiver distress. The aims of this study were to clarify the differences of trajectories of 12 kinds of BPSDs by disease severity in four major dementias and to develop charts showing the frequency, severity, and associated caregiver distress (ACD) of BPSDs using the data of a Japan multicenter study (J-BIRD).

**Methods:**

We gathered Neuropsychiatric Inventory (NPI) data of patients with Alzheimer’s disease (AD; n = 1091), dementia with Lewy bodies (DLB; n = 249), vascular dementia (VaD; n = 156), and frontotemporal lobar degeneration (FTLD; n = 102) collected during a 5-year period up to July 31, 2013 in seven centers for dementia in Japan. The NPI composite scores (frequency × severity) of 12 kinds of items were analyzed using a principal component analysis (PCA) in each dementia. The factor scores of the PCA were compared in each dementia by disease severity, which was determined with Clinical Dementia Rating (CDR).

**Results:**

Significant increases with higher CDR scores were observed in 1) two of the three factor scores which were loaded for all items except euphoria in AD, 2) two of the four factor scores for apathy, aberrant motor behavior (AMB), sleep disturbances, agitation, irritability, disinhibition, and euphoria in DLB, and 3) one of the four factor scores for apathy, depression, anxiety, and sleep disturbances in VaD. However, no increases were observed in any of the five factor scores in FTLD.

**Conclusions:**

As dementia progresses, several BPSDs become more severe, including 1) apathy and sleep disturbances in AD, DLB, and VaD, 2) all of the BPSDs except euphoria in AD, 3) AMB, agitation, irritability, disinhibition, and euphoria in DLB, and 4) depression and anxiety in VaD. Trajectories of BPSDs in FTLD were unclear.

## Introduction

Behavioral and psychological symptoms of dementia (BPSDs) negatively impact the prognosis of dementia patients [1] and increase caregiver distress [2], and accelerate the need for institutionalization [3–5]. The first step in treating BPSD involves non-pharmacological therapies [6]. Effective non-pharmacological therapies include group activities and music therapy for agitation and depression [7–9], and music therapy [10] and cognitive behavioral therapy [11] for anxiety. However, these therapies must be implemented according to clearly defined programs devised by specialists, and cannot be administered by non-professionals. If no improvements are seen with non-pharmacological therapies, pharmacological therapy should be considered [6]. Cholinesterase inhibitors [12–14] and memantine [15] can be used against BPSD in Alzheimer’s disease (AD), while rivastigmine [16] and donepezil [17] are used for dementia with Lewy bodies (DLB). The effectiveness of these drugs is often insufficient, in which case, atypical antipsychotic drugs are an option. However, adverse events often occur with atypical antipsychotics, and their effectiveness is limited.

It is important that BPSDs are detected soon after onset while the symptoms are still mild. At this time, they should be handled according to the general standards recommended by the American Psychiatric Association Work Group on Alzheimer's Disease and Other Dementias [18]. Policies that can help to prevent BPSD progression in dementia patients include keeping requests and demands relatively simple, deferring requests if the patient becomes overly upset or angered, avoiding overly complex tasks that may lead to frustration, and so on. The complete guide to BPSD developed by the international psychogeriatric association is also available online (https://www.ipa-online.org/publications/guides-to-bpsd). In order to prevent progression of BPSD, it is important that family caregivers, who spend a long time with and are closest to the patient, detect BPSD as quickly as possible. Knowledge about which symptoms are likely to occur, and in which patients can facilitate early detection, as observation can be focused on symptoms that have a high possibility of occurring, and reduce the chance that they might be overlooked. Different BPSDs are associated with different dementias; for example, visual hallucinations often occur in DLB, while disinhibition is common in frontotemporal lobar degeneration (FTLD) [19]. Symptom characteristics also differ depending on disease severity. Agitation and disinhibition are more severe in AD and vascular dementia (VaD) patients with moderate dementia (clinical dementia rating scale score 2 (CDR 2)) than in patients with mild dementia (CDR 1) [20]. However, no studies to date have comprehensively investigated BPSD frequency, severity, and associated caregiver distress in a large number of patients.

The present study collected BPSD data from seven specialized centers for dementia in Japan. The first aim of this study was to clarify the effect of dementia severity on 12 kinds of BPSDs in four major dementias (AD, DLB, VaD, and FTLD). The second aim was to develop charts that summarize the frequency, severity, and associated caregiver distress (ACD) of 12 BPSDs in the four types of dementia.

## Methods

This study was part of a large, multicenter project conducted in Japan, "The Japan multicenter study: Behavioral and psychological symptoms Integrated Research in Dementia (J-BIRD)". The main aim of the J-BIRD project was the development of strategies to prevent and treat BPSDs based on how and why they arise in patients with dementia. Seven specialized centers for dementia in Japan (J-BIRD centers, see Acknowledgement) participated in the project. This retrospective observational study was conducted without intervention and in compliance with national legislation and the Declaration of Helsinki. All patient information was anonymized and stored as unlinked data prior to analysis in order to prevent the disclosure of personal information. No monetary incentives were provided to the participants or caregivers. The study was undertaken after obtaining approval from the ethics committee at one of the seven participating centers, Osaka University Hospital. The ethics committees of the other six centers considered this study a quality-controlled study that did not necessitate ethical approval.

### Procedures

At the outpatient departments of the J-BIRD centers, patients were examined by psychiatrists and neurologists specializing in dementia, as well as routine laboratory tests and standard neuropsychological examinations. They were also evaluated in terms of activities of daily living. Based on these data, CDR scores are calculated to determine disease severity [21]. BPSDs are evaluated using the Neuropsychiatric Inventory (NPI) [22]. In addition, electroencephalography, brain magnetic resonance (MR) imaging, and/or cerebral perfusion studies with single-photon emission computed tomography, cerebrospinal fluid examination including concentrations of amyloid β42, total tau, and hyperphosphorylated tau, were performed. Dementia type was diagnosed based on the results of all tests, and all data were registered in the clinical databases at each facility.

In the present study, NPI, demographic and clinical data were collected regarding patients who came to the J-BIRD centers for the first time during the 5-year period from August 1, 2008 to July 31, 2013 and were suspected to have dementia. The NPI data were collected by each facility using one of the following four surveys: the original 10-item survey [22]; a 12-item survey comprising the 10-item survey plus eating abnormalities and sleep disturbances [23]; an 11-item survey comprising the 10-item survey plus cognitive fluctuation [24]; and a 13-item survey comprising all items. ACD of each item was evaluated according to NPI Caregiver Distress Scale (NPI-D) [25]. We collected all of the NPI data of the four surveys and used 12 BPSDs except for cognitive fluctuation in this study.

The present investigation focused on patients with AD, VaD, DLB, and FTLD, which are commonly occurring dementias. The diagnosis of dementia was made according to the criteria of the Diagnostic and Statistical Manual of Mental Disorders, 3rd edition-revised (DSM-III-R) [26]. Diagnoses of AD, VaD, DLB, and FTLD were based on criteria established by the National Institute of Neurological and Communicative Disorders and Stroke/Alzheimer’s Disease and Related Disorders Association [27]; the International Workshop convened by the National Institute of Neurological Disorders and Stroke with support from the Association Internationale pour la Recherche et l'Enseignement en Neurosciences [28], and/or the Alzheimer's Disease Diagnostic and Treatment Centers [29]; the Consortium on DLB International Workshop [30]; and the International collaborative workshop on FTLD [31], respectively. We collected all of the three kinds of FTLD, frontotemporal dementia (behavioral variant of FTD), semantic dementia, and progressive nonfluent aphasia.

In order to reflect BPSD status from a clinical perspective, we included the data of patients with AD, DLB, and FTLD with complications of cerebral vascular disease (CVD), which could not solely be a cause for dementia. However, patients with two or more concomitant dementias were excluded from the analysis. Patients without a definitive diagnosis and those with developmental abnormalities, serious psychiatric diseases, substance abuse, or significant neurologic antecedents such as brain trauma, brain tumor, epilepsy, and inflammatory disease, were excluded. Patients with one or more missing NPI data item were analyzed based on the available data; however, those with missing total CDR scores or with CDR scores that were 0 were excluded.

### Statistical analysis

The NPI composite scores (frequency × severity) of 12 kinds of items were analyzed using a principal component analysis (PCA) to explore the structure of the NPI. Although the same factor solution is applicable in the comparison of AD, VaD, DLB and Parkinson’s disease [32], we were not confident that the same factor solution could be used when FTLD is included, so we used separate PCAs for each type of dementia. Before carrying out the PCA, the suitability of data and the factorability of the correlation matrix were assessed by calculating the Kaiser-Meyer-Olkin Measure of Sampling Adequacy (KMO) and Barlett’s Test of Sphericity. The PCA was used to analyze the inter-item relationship and to extract the initial factors, and then a Varimax rotation was performed. The number of factors to be retained was determined by examining the eigenvalues exceeding 1.0 and by examining a scree plot. Items with factor loadings ≥ 0.30 were entered into a factor. A factor analysis accompanies factor scores, which can be used as variables in subsequent statistical analyses. We compared the factor scores at four stages of disease severity (CDR 0.5, 1, 2, and 3) using the Kruskal-Wallis test. Significance was set at p<0.05. Items with a significant difference were then subjected to post hoc tests using of the Mann-Whitney U. Significance in the post hoc tests was set at p<0.05/6 = 0.0083. As additional analyses, we compared NPI frequency, severity, and ACD scores for each of the 12 BPSD at four stages of disease severity using the Kruskal-Wallis test. Items with a significant difference were then subjected to post hoc tests using of the Mann-Whitney U. The PCA was conducted using the data of patients who had all of the 12 kinds of BPSDs in the NPI. Additional analyses were conducted using the data of patients who had each of the 12 kinds of BPSD.

The same analyses were repeated for each type of dementia. All statistical analyses were performed with SPSS18.0 (SPSS Inc. Chicago, Illinois).

### Chart development

Family caregivers have few opportunities to compare BPSD for different dementias. The present charts are primarily intended to enable family caregivers to understand how BPSD will change as the relevant dementia progresses. During chart development for AD, data for patients with a diagnosis of AD were extracted from the overall NPI data. Then, one of the 12 BPSDs was selected, and bar graphs were created for “Frequency,” “Severity,” and “Caregiver distress,” respectively. On the NPI, symptom frequency was scored as follows: 0, never; 1, less than once a week; 2, about once a week; 3, more than once a week but less than once a day; and 4, at least once a day. The proportions of the patients in each score group were represented in one bar on the ‘Frequency’ graph. Separate bars were created for each CDR group. Bar graphs were then created in the same way for “Severity” and “Caregiver distress”. Data from patients with a frequency score of “0, none” for BPSD were excluded from the graphs for “Severity” and “Caregiver distress”. This process was performed for all 12 BPSDs and then repeated for the other three dementias.

For each BPSD, patients with an NPI severity score of 2 or more were categorized as “patients with S≥2,” and patients with moderate or greater caregiver distress were categorized as “patients with ACD≥M”. These groups were regarded as those with considerable severity or caregiver distress. We also focused on low frequency (LF) BPSDs that caused high caregiver distress (BPSDs-LF/HCD). We regarded BPSDs whose frequency was less than 20% but the percentage of patients with ACD≥M was more than 50% as BPSDs-LF/HCD in this study. Whether the BPSDs met the criteria for BPSDs-LF/HCD was determined in each of 192 groups (12 BPSDs × 4 kinds of CDR scores × 4 types of dementia).

## Results

Data were collected for a total of 2447 patients in the J-BIRD. Patients with mild cognitive impairment (MCI; n = 293), normal pressure hydrocephalus (n = 161), subjective cognitive impairment (n = 46), corticobasal degeneration (n = 32), progressive supranuclear palsy (n = 26) and CVD without dementia (n = 4) were excluded from the study. The remaining patients, who were the subjects of this study, included 1301 AD patients, 269 DLB patients, 191 VaD patients, and 124 FTLD patients.

### Alzheimer’s disease (AD)

Of the 1301 AD patients, six had CDR 0, 161 had incomplete CDR data, one had no NPI data, and 42 had other dementias; these patients were excluded. Therefore, the AD charts (S1 File) were finally developed based on 1091 patients (339 men, 752 women; mean age was 76.9±8.7 years; mean mini-mental state examination (MMSE) score was 18.9±5.4; mean length of education was 10.8±2.8 years). The complete demographic data for the patients with all for dementias are given in S1 Table.

The results of PCA for NPI composite scores classified the 12 BPSDs into three factors (S2 Table). The BPSD that were loaded into Factor 1 were delusions, agitation, depression, anxiety, disinhibition, and irritability; Factor 2 included hallucinations, apathy, aberrant motor behavior (AMB), sleep disturbances, and eating abnormalities; and Factor 3 included hallucinations, euphoria and disinhibition. Significant differences were observed between the CDR groups in Factors 1 and 2, but not Factor 3 (Table 1). Post hoc test results showed that the scores of Factors 1 and 2 increased with higher CDR scores.

**Table 1:** NPI factor scores according to dementia severity in patients with four dementias. No. patients shown in bold.

	CDR					
Factor (BPSDs^1^)	0.5	1	2	3	p value^2^	Post hoc test^3^
**Alzheimer’s disease**	**261**	**336**	**142**	**30**		
1 (Del, Agit, Dep, Anx, DI, Irrit)	-0.24±0.06	0.03±0.05	0.31±0.08	0.38±0.18	<0.001	0.5<1, 2, 3; 1<2
2 (Hal, Ap, AMB, SD, EA)	-0.43±0.06	0.09±0.05	0.36±0.08	1.07±0.17	<0.001	0.5<1, 2, 3; 1<2, 3
3 (Hal, Eup, DI)	-0.01±0.06	-0.06±0.05	0.17±0.08	-0.05±0.18	0.124	
**Dementia with Lewy bodies**	**43**	**69**	**41**	**9**		
1 (Del, Hal, Agit, Irrit, EA)	0.04±0.15	-0.06±0.12	0.06±0.16	0.02±0.34	0.963	
2 (Dep, Anx, EA)	0.13±0.15	0.02±0.12	-0.06±0.16	-0.49±0.33	0.173	
3 (Ap, AMB, SD)	-0.30±0.14	-0.14±0.11	0.32±0.15	1.06±0.32	<0.001	0.5<2, 3; 1<3
4 (Agit, Eup, Ap, DI, Irrit)	-0.15±0.15	-0.18±0.12	0.40±0.15	0.28±0.33	<0.001	0.5<2; 1<2
**Vascular dementia**	**23**	**49**	**21**	**6**		
1 (Agit, DI, Irrit, AMB, SD)	-0.24±0.20	-0.17±0.13	0.28±0.20	1.34±0.38	0.036	
2 (Dep, Anx, Ap, SD)	-0.33±0.21	-0.03±0.14	0.28±0.22	0.48±0.40	0.006	0.5<1, 2
3 (Eup, AMB, SD, EA)	0.13±0.21	-0.11±0.14	0.07±0.22	0.15±0.41	0.908	
4 (Del, Hal)	0.05±0.21	-0.04±0.14	-0.09±0.22	0.45±0.41	0.183	
**Frontotemporal lobar degeneration**	**23**	**18**	**17**	**4**		
1 (Del, Agit, Eup, DI, Irrit, EA)	-0.30±0.20	0.42±0.23	0.07±0.24	-0.42±0.49	0.245	
2 (Hal, Anx, Eup, SD)	-0.06±0.20	-0.07±0.23	-0.17±0.23	1.38±0.48	0.513	
3 (Del, AMB, SD)	-0.40±0.20	-0.07±0.22	0.49±0.23	0.52±0.47	0.045	
4 (Ap, AMB, EA)	-0.34±0.20	-0.13±0.23	0.44±0.23	0.64±0.48	0.037	
5 (Del, Dep, Anx)	-0.003±0.21	0.19±0.24	-0.08±0.25	-0.51±0.51	0.716	

^1^Del, delusions; Hal, hallucinations; Agit, agitation; Dep, depression; Anx, anxiety; Eup, euphoria; Ap, apathy; DI, disinhibition; Irrit, irritability; AMB, aberrant motor behavior; SD, sleep disturbances; EA, eating abnormalities; CDR: clinical dementia rating

^2^Comparison between 4 CDR groups, Kruskal-Wallis test

^3^Mann-Whitney U test (p <0.05/6 = 0.0083)

When the associations between dementia severity and each of frequency, severity, and ACD were separately analyzed, significant differences were observed between the four CDR groups in NPI frequency scores for all BPSDs excluding euphoria; in NPI severity scores for agitation, apathy, irritability and sleep disturbances; and in NPI-ACD scores for agitation, depression, anxiety, apathy, AMB, sleep disturbances, and eating abnormalities (S3 Table). Post hoc test results also showed increased NPI frequency, severity, and ACD scores with higher CDR scores for all BPSDs except NPI severity score for agitation in which significant differences were observed in the Kruskal-Wallis test.

Apathy was the most prevalent BPSD in all CDR groups and only the symptom with frequencies exceeding 50% in AD patients with CDR 0.5, 1, and 2 (Table 2 and S4 Table). The number of symptoms with proportions of patients with S≥2 were 50% or more were higher in patients with CDR 2 and CDR 3 than those with CDR 0.5 and CDR 1. The symptoms whose prevalence of ACD≥M exceeded 50% were included in the symptoms whose prevalence of S≥2 were 50% or more, except for anxiety in CDR 3 patients. Disinhibition in AD patients with CDR 0.5 met the criteria for BPSDs-LF/HCD in this study. The frequencies of hallucinations were very low for CDR 0.5 and 1 patients, at 4.6% and 7.0%, respectively, but increased to 14.5% in CDR 2 and again to 35.5% in CDR 3 patients.

**Table 2:** Symptoms whose prevalence of dementia patients were 50% or more in each CDR score. Symptoms in each column are shown in decreasing order of prevalence. Only symptoms that were found in at least 5 patients are shown in the table.

	CDR^1^			
NPI^2^ Prevalence	0.5	1	2	3
**Alzheimer’s disease**				
frequency≥1	Ap	Ap	Ap	Ap, Agit
severity≥2	DI, EA	EA	DI, AMB, Ap, Agit, Eup	SD, AMB, Agit, Ap, EA
ACD≥M^3^	DI		DI, Agit	SD, Agit, EA, AMB, Anx
BPSDs-LF/HCD^4^	DI			
**Dementia with Lewy bodies**				
frequency≥1	Hal, Ap, Del	Ap, Hal, Del, SD	Ap, Del, Hal, SD	Ap, SD, Del, Hal, AMB
severity≥2	EA, Del, Hal, Dep, DI	EA, Del, Irit, Hal, AMB	Ap, DI, Del, AMB, SD, EA	SD, Ap, AMB, Del, Agit
ACD≥M	Anx	DI, Agit, Del	DI, Irit, Agit, Del, SD	Agit, SD
BPSDs-LF/HCD		DI	DI	
**Vascular dementia**				
frequency≥1	Ap	Ap	Ap, SD, Agit	Ap, SD, Agit, Irrit, Del
severity≥2	Agit, Del, EA	AMB, Del, Ap, Agit, EA	Irrit, Del, Ap, DI, AMB, SD, Agit	DI, Del, SD, Ap, AMB, Agit, Irrit
ACD≥M	Del	DI, Del, Agit, Irrit	Anx, Agit, Del, Dep, SD, Irrit	Agit, SD, Ap, Dep, Irrit
BPSDs-LF/HCD	Del	Del		
**Frontotemporal lobar degeneration**				
frequency≥1	Ap	EA, Ap, Agit, Irrit	Ap, Agit, DI, AMB, EA, SD, Irrit	Ap, SD, EA, AMB
severity≥2		AMB, EA	Ap, AMB, Agit, SD, Irrit, DI, EA	Ap
ACD≥M	Irrit		DI, Irrit, AMB, SD, Agit	Ap
BPSDs-LF/HCD	Irrit			

Del, delusions; Hal, hallucinations; Agit, agitation; Dep, depression; Anx, anxiety; Eup, euphoria; Ap, apathy; DI, disinhibition; Irrit, irritability; AMB, aberrant motor behavior; SD, sleep disturbances; EA, eating abnormalities

^1^CDR: clinical dementia rating

^2^NPI, Neuropsychiatric inventory

ACD≥M ^3^: Moderate or greater associated caregiver distress

BPSDs-LF/HCD^4^: BPSD whose frequency was less than 20% but the percentage of patients with ACD≥M was more than 50%.

### Dementia with Lewy bodies (DLB)

Of the 269 DLB patients, three were CDR 0, 14 had incomplete CDR data, one had no NPI data, and 2 had other dementias; these patients were therefore excluded. Finally, the DLB charts (S2 File) were developed based on 249 patients (102 men, 147 women; mean age, 78.9±5.9 years; mean MMSE score, 19.0±5.4; mean length of education, 10.5±2.7 years) (S1 Table).

The results of PCA for NPI composite scores classified the 12 BPSD into four factors (S5 Table). The BPSDs that were loaded into Factor 1 were delusions, hallucinations, agitation, irritability, and eating abnormalities; Factor 2 included depression, anxiety, and eating abnormalities; Factor 3 included apathy, AMB, and sleep disturbances; and Factor 4 included agitation, euphoria, apathy, disinhibition, and irritability. Significant differences were observed between the four CDR groups in scores of the Factors 3 and 4, but not in Factors 1 and 2 (Table 1). Post hoc test results showed the scores of the two factors increased with higher CDR scores. Significant differences were observed between the four CDR groups in NPI frequency scores for delusions, agitation, apathy, AMB, and sleep disturbances; and in NPI severity scores for apathy (S6 Table). No differences were observed in NPI-ACD scores. Post hoc test results showed increased frequency in agitation, apathy, and AMB, and increased severity in apathy with higher CDR scores.

The most prevalent symptom was hallucinations in DLB patients with CDR 0.5 and apathy in patients with CDR 1, 2, and 3 (Table 2 and S7 Table). BPSDs with frequencies that exceeded 50% in all four CDR groups were delusions, hallucinations, and apathy. Frequencies of sleep disturbances also exceeded 50% for all CDR groups except for CDR 0.5 (48.9%). The proportions of patients with S≥2 were 50% or more in many BPSDs in DLB patients with all CDR scores. The proportions of patients with ACD≥M were also 50% or more in five BPSDs in patients with CDR 2. Disinhibition in DLB patients with CDR 1 and CDR 2 met the criteria for BPSDs-LF/HCD in this study.

### Vascular dementia (VaD)

Of the 191 VaD patients, 21 had incomplete CDR data and 14 had other dementias were excluded; therefore, the VaD charts (S3 File) were developed based on 156 patients (80 men, 76 women; mean age, 75.9±10.0 years; mean MMSE score, 19.6±5.0; mean length of education, 10.8±2.7 years) (S1 Table).

The results of PCA for NPI composite scores classified the 12 BPSD into four factors (S8 Table). The BPSDs that were loaded into Factor 1 were agitation, disinhibition, irritability, AMB, and sleep disturbances; Factor 2 included depression, anxiety, apathy, and sleep disturbances; Factor 3 included euphoria, AMB, sleep disturbances, and eating abnormalities; and Factor 4 included delusions and hallucinations. Significant differences of factor scores were observed between the four CDR groups in Factors 1 and 2, but not in Factors 3 and 4 (Table 1). Post hoc test results showed the scores of Factor 2 were higher in patients with CDR 1 and 2 than in patients with CDR 0.5.

Significant differences were observed between the four CDR groups in NPI frequency scores for delusions, agitation, apathy, AMB, and sleep disturbances; in NPI severity scores for apathy, irritability, and sleep disturbances; and in NPI-ACD scores for anxiety, apathy, and sleep disturbances (S9 Table). Post hoc test results showed increased frequency in delusions, agitation, apathy, AMB, and sleep disturbances; and increased severity in apathy, irritability, and sleep disturbances; and increased ACD in anxiety, apathy, and sleep disturbances with higher CDR scores.

Apathy was the most common BPSD in VaD patients with all CDR scores (Table 2 and S10 Table). BPSDs with frequencies of 50% or more included sleep disturbances and agitation in VaD patients with CDR 2 and CDR 3, and irritability and delusions in patients with CDR 3. The numbers of symptoms whose proportions of patients with S≥2 and with ACD≥M were 50% or more were equal to or greater than the number of symptoms whose frequencies were 50% or more. Delusions in VaD patients with CDR 0.5 and CDR 1 met the criteria for BPSDs-LF/HCD in this study.

### Frontotemporal lobar degeneration (FTLD)

Of the 124 FTLD patients, five were CDR 0 and 17 had incomplete CDR data; these patients were therefore excluded. Finally, the FTLD charts (S4 File) were developed based on 102 patients (52 men, 50 women; mean age, 69.9±8.4 years; mean MMSE score, 18.2±6.9; mean length of education, 11.7±2.8 years) (S1 Table).

The results of PCA for NPI composite scores classified the 12 BPSDs into five factors (S11 Table). The BPSD that were loaded into Factor 1 were delusions, agitation, euphoria, disinhibition, irritability, and eating abnormalities; Factor 2 included hallucinations, anxiety, euphoria, and sleep disturbances; Factor 3 included delusions, AMB, and sleep disturbances; Factor 4 included apathy, AMB, and eating abnormalities; and Factor 5 included delusions, depression and anxiety. Significant differences of factor scores were observed between the four CDR groups in Factors 3 and 4, but not in other factors (Table 1). However, post hoc test results showed there were no pairs of patients with each CDR score whose factor scores of Factors 3 and 4 were significantly different.

Significant differences were observed between the four CDR groups in the NPI frequency scores for hallucinations, apathy, disinhibition, irritability, AMB, and sleep disturbances; in NPI severity scores for apathy; and in NPI-ACD scores for disinhibition and sleep disturbances (S12 Table). Post hoc test results showed increased frequency in hallucinations, apathy, irritability, and AMB; and increased severity in apathy; and increased ACD in disinhibition with higher CDR scores.

Apathy was the most prevalent BPSD in FTLD patients with CDR 0.5 (68.6%), 2 (92%) and 3 (100%) (Table 2 and S13 Table). The proportions of CDR 3 patients with S≥2 and with ACD≥M for apathy were 85.8% and 71.5%, respectively, indicating that apathy severity and ACD were also high in patients with CDR 3. The most prevalent symptom in CDR 1 patients was eating abnormalities and the frequency of the symptom was the highest in CDR 1 among all CDR scores, at 77.8%. Although the frequencies of delusions and hallucinations were low in all CDR groups, both symptoms had a frequency of 28.6% in CDR 3 patients. Compared with the other three dementias, the frequency of euphoria was high in FTLD, at 11.4% in CDR 0.5 and 32% in CDR 2 patients. Irritability in FTLD patients with CDR 0.5 met the criteria for BPSDs-LF/HCD in this study.

## Discussion

The J-BIRD study collected NPI data from 2447 consecutive patients who came to the J-BIRD centers for the first time during a 5-year period and were suspected of having dementia. Of the 2447 patients, analyses in this study were conducted on 1091 AD, 249 DLB, 156 VaD, and 102 FTLD patients to clarify the trajectories of BPSD in four types of dementia by disease severity. Although previous studies have investigated BPSD by dementia type and disease severity, the study populations were small. Hirono et al. [19] investigated 240 AD, 23 DLB, and 24 FTLD patients; Srikanth et al. [20] investigated 44 AD, 31 VaD, and 23 FTLD patients; and Hashimoto et al.[33] investigated 393 AD and 97 DLB patients. The population of the present study was considerably larger than the previous ones. Although it has been reported that BPSDs vary greatly even in one type of dementia [34], the J-BIRD data allow the effects of disease severity on the frequency, severity, and ACD of each BPSD in each dementia to be clarified. Studies to date regarding the association between disease severity and BPSD have mainly investigated patients up to CDR 2 [20, 33], which enhances the value of the CDR 3 patient data obtained in the present study. As the four types of dementia covered in the present study represent 92.2% of all dementias in Japan [35], the present charts for BPSD should be applicable for the majority of family caregivers of dementia patients.

### BPSDs at CDR 0.5 and how to use the BPSD charts

The J-BIRD data reflect the present situation of clinic visits of dementia patients because it is comprised of data of consecutive, first-time patients. The most prevalent CDR score of the participants in this study was CDR 1 (43.6%), followed by CDR 0.5 (31.9%), 2 (19.6%), and 3 (4.9%), indicating that dementia patients often visit dementia centers when the patients are at CDR 0.5 or 1. The results of this study showed that various kinds of BPSD were observed with high frequency even in patients with CDR 0.5. The BPSDs with frequencies exceeding 20% at CDR 0.5 were 5 symptoms in AD, 9 symptoms in DLB, 7 symptoms in VaD, and 8 symptoms in FTLD. When a patient and his/her caregiver are given a diagnosis of causal disease of dementia and dementia severity at a hospital, they should also be given the information about which kinds of BPSD are likely to occur at the stage of dementia. Information about the BPSDs that are likely to occur at the next stage of dementia should also be given to a patient and his/her caregiver. They should be instructed not to overlook the BPSDs and to handle them soon after onset while the symptoms are still mild.

### Trajectories of BPSDs by dementia severity

In this study, we conducted a PCA to integrate 12 kinds of NPI composite score into 3 to 5 factors in each dementia to grasp the associations between BPSD and severity of dementia. The PCA findings demonstrated that disease severity had different effects on BPSD depending on the BPSD and dementia type. The unique pathophysiological process of each kind of dementia may contribute to this variability. The associations between dementia severity and frequency, severity, and ACD were separately analyzed in this study. The frequency and the degrees of severity and ACD were not always correlated with each other in this study, which is consistent with the results of a previous study [25]. The ACD was not always in line with the frequency and severity of BPSD, possibly because it was related to a general summation of patients’ emotional, behavioral, and functional problems.

#### AD patients

In AD patients, the scores of two of the three factors from the PCA, consisting of 11 kinds of BPSD, except for euphoria, increased with increasing CDR scores. The trajectories of the two factor scores are similar to the trajectories of frequency of BPSD in AD patients: in that the frequency of all of the BPSDs except euphoria increased with increasing CDR scores. However, the progression of dementia was significantly associated with the severity of BPSD in only 3 symptoms in AD patients. Although differences in statistical power due to sample size between patients with NPI frequency score and those with NPI severity score may have had some effect, the present findings show that as AD progressed, the frequency increased but the severity of most symptoms did not change.

#### DLB patients

The frequencies and severities of many BPSDs at the early stage of dementia were higher in DLB patients than in AD patients in this study. The PCA revealed that in DLB patients, two of the four factors, consisting of 7 BPSDs (apathy, AMB, sleep disturbances, agitation, euphoria, disinhibition, and irritability) increased as the disease progressed. There were no significant differences in the factor scores of the rest of the two factors, which were loaded for delusions, hallucinations, depression, anxiety, and eating abnormalities, between the four CDR groups in this study. These findings indicated that the progressions of BPSD are not always associated with the progression of dementia in DLB patients, which was consistent with the results of our previous study [33]. We should inform DLB patients and their caregivers that patients with DLB are likely to have various and severe BPSDs in the early stage of dementia and some BPSD symptoms increase with progression of the dementia.

#### VaD patients

The results of the analyses of factor score from the PCA indicated that apathy, sleep disturbances, anxiety, and depression increased with higher CDR scores in VaD patients. The percentages of patients with S≥2 and those with ACD≥M were high in delusions, hallucinations, agitations, depression, and euphoria in VaD patients compared to AD patients in almost all of the CDR groups, especially in VaD patients with CDR 3. These results contradicted the results of a previous report that in severe stages, VaD patients impose a milder distress on relatives than do AD patients [36]. The reason for the discrepancy is unclear, but it might be because our patients had more severe cases of VaD than did those in the previous study. This might also explain the lower prevalence of VaD in our study compared to that in the general population [37]

#### FTLD patients

To our knowledge, no studies have clarified the associations between BPSD and CDR scores in FTLD patients. The PCA in this study revealed that there were no factor scores which were significantly different according to dementia severity in FTLD patients, indicating that NPI composite scores did not change with dementia progression in FTLD. However, significant increases of frequency with increasing CDR were observed between some pairs of CDR groups in hallucinations, apathy, irritability, and AMB in FTLD patients in this study. Significant increase of severity with increasing CDR was also observed in apathy. These findings indicated that some BPSDs increased with progression of the dementia in FTLD patients. The frequencies of eating abnormalities were higher in FTLD patients than in AD patients in all CDR groups. Eating abnormalities in the NPI include rigid dietary preferences, pathological sweet tooth, rushing through meals, and refusing to eat [38]. The present study did not collect data on types of eating abnormalities, which may differ depending on CDR.

### BPSDs with low frequency and high caregiver distress

In this study, we focused on low frequency BPSDs that caused high caregiver distress because these symptoms are likely to be overlooked but strongly affect the quality of life of the patients and caregivers. In this study, we would like to discuss the BPSDs-LF/HCD whose percentage of patients with ACD≥M were calculated based on 5 or more patients. The BPSDs-LF/HCD in this study were disinhibition in AD patients with CDR 0.5 and DLB patients with CDR 1 and 2 and delusions in VaD patients with CDR 0.5 and 1. The percentages of patients with ACD≥M for disinhibition were around 50% or more in all dementias with all CDR scores, except for DLB with CDR 0.5, and FTLD with CDR 0.5 in this study. These findings indicated that disinhibition causes greater caregiver distress. Delusions in VaD patients with CDR 0.5 and 1 cause greater caregiver distress than do delusions in AD patients with CDR 0.5 and 1. The present result is consistent with a previous finding that in the early stages, VaD patients impose a greater distress on relatives than do AD patients [36].

### Hallucinations

Hallucinations were present in a high proportion (35.5%) of the 45 AD patients with CDR 3. Although it is unclear whether Hirono et al. included CDR 3 patients in their study, as CDR scores were not reported, the overall frequency of hallucinations in AD patients was low, at 5% [19]. In the study by Srikanth et al. [20], two of the 44 AD patients were CDR 3, and the rest were CDR 1 or CDR 2, and the overall proportion of patients with hallucinations was 4.6%. The proportions of CDR 0.5 and CDR 1 AD patients with hallucinations in the present study, at 4.6% and 7.0%, respectively, were similar to those of previous studies. However, the frequency of hallucinations in CDR 2 patients was high, at 14.5%, suggesting that hallucinations occur more frequently in AD patients as dementia progresses. These results do not contradict previous findings that AD patients with visual hallucinations present with worse cognitive impairment [39] and visuospatial abilities [40] than do those without visual hallucinations. Hallucinations were observed in two of the seven FTLD patients with CDR 3 (28.6%) in this study, although the small number of patient means the reliability of this finding may be low. The present study showed a possibility of hallucinations in FTLD patients, particularly in cases of severe dementia. Although hallucinations were not observed in any patients with FTLD in studies by Hirono et al. [19] and Srikanth et al. [20], a recent study reported that 20.6% of 97 FTLD patients had paranoid ideas, and that 17.5% of them had hallucinations and delusions [41]. In addition, psychotic symptoms are predominantly observed in patients with right-sided brain degeneration and the majority of patients with the psychotic symptoms were tau-negative [41]. Hallucinations were associated with high caregiver distress despite low corresponding frequency and severity, possibly because they are unsettling for the family.

### Apathy

Apathy was the most common BPSD in all dementias in this study. The frequency of apathy was at least 50% from CDR 0.5 onwards in all dementias, and increased as the disease progressed. Apathy was associated with atrophy of the medial frontal regions, and increasing severity of the symptom was associated with tissue loss in these regions in degenerative dementia patients, including those with AD and FTLD [42]. Apathy was observed in at least 90% of CDR 3 patients in all four diseases. Proportions of patients with S≥2 and with ACD≥M for apathy were 64.3% or less and 50.0% or less in AD or DLB patients with CDR 3. However, proportions of patients with S≥2 and with ACD≥M for apathy were 85.8% and 71.5% in FTLD patients with CDR 3. This is thought to be due to the high degree of damage in the medial frontal regions in FTLD.

### Limitations

The present study has several limitations. First, sample sizes for the dementias other than AD were small, therefore, the differences of results between AD and the other three dementias may be attributable to differences in patient number rather than disease characteristics. Second, as there were few patients with CDR 3 overall, the reliability of these data is low compared to patients with CDR 0.5, 1, and 2.

## Conclusions

The trajectories of 12 kinds of BPSDs by disease severity were different for each of the four dementias. Eleven BPSDs in AD, seven BPSDs in DLB and four BPSDs in VaD increased with increasing disease severity, while none of the BPSDs in FTLD were affected by disease severity. With the progression of dementia, the trajectories of the frequency of BPSDs were not always similar to the trajectories of the severity and ACD of BPSDs. Four of our results will help caregivers to better understand their patients: 1) The frequencies and severities of many BPSDs at the early stage of dementia were higher in DLB patients than in AD patients. 2) Disinhibition, which causes great caregiver distress, was not frequent in AD or DLB patients. 3) Hallucinations were present in a high proportion in AD and FTLD patients with CDR 3. 4) Apathy was the most common BPSD in all dementias. Charts developed for this study will assist doctors, nurses, and care specialists in explaining preventive measures for each type of BPSD to family caregivers.

## Supporting Information

S1 File Charts for BPSD in Alzheimer’s disease by disease severity. (PDF)

S2 File Charts for BPSD in Dementia with Lewy bodies by disease severity. (PDF)

S3 File Charts for BPSD in Vascular dementia by disease severity. (PDF)

S4 File Charts for BPSD in Frontotemporal lobar degeneration by disease severity. (PDF)

S1 Table Demographic data of patients with four dementias in this study. (DOCX)

S2 Table Factor loadings for BPSDs in patients with Alzheimer’s disease. (DOCX)

S3 Table Neuropsychiatric Inventory scores of individual domains according to dementia severity in patients with Alzheimer’s disease. (DOCX)

S4 Table Percentages of patients of individual domains according to dementia severity in patients with Alzheimer’s disease. (DOCX)

S5 Table Factor loadings for BPSDs in patients with Dementia with Lewy bodies. (DOCX)

S6 Table Neuropsychiatric Inventory scores of individual domains according to dementia severity in patients with Dementia with Lewy bodies. (DOCX)

S7 Table Percentages of patients of individual domains according to dementia severity in patients with Dementia with Lewy bodies. (DOCX)

S8 Table Factor loadings for BPSDs in patients with Vascular dementia. (DOCX)

S9 Table Neuropsychiatric Inventory scores of individual domains according to dementia severity in patients with Vascular dementia. (DOCX)

S10 Table Percentages of patients of individual domains according to dementia severity in patients with Vascular dementia. (DOCX)

S11 Table Factor loadings for BPSDs in patients with Frontotemporal lobar degeneration. (DOCX)

S12 Table Neuropsychiatric Inventory scores of individual domains according to dementia severity in patients with Frontotemporal lobar degeneration. (DOCX)

S13 Table Percentages of patients of individual domains according to dementia severity in patients with Frontotemporal lobar degeneration. (DOCX)

## References

[1] Stern Y, Mayeux R, Sano M, Hauser WA, Bush T. Predictors of disease course in patients with probable Alzheimer's disease. Neurology. 1987;37(10):1649–53. . 3658173 10.1212/wnl.37.10.1649

[2] Allegri RF, Sarasola D, Serrano CM, Taragano FE, Arizaga RL, Butman J, et al Neuropsychiatric symptoms as a predictor of caregiver burden in Alzheimer's disease. Neuropsychiatric disease and treatment. 2006;2(1):105–10. 19412452 PMC2671738

[3] Magni E, Binetti G, Bianchetti A, Trabucchi M. Risk of mortality and institutionalization in demented patients with delusions. Journal of geriatric psychiatry and neurology. 1996;9(3):123–6. . 8873875 10.1177/089198879600900303

[4] Morriss RK, Rovner BW, Folstein MF, German PS. Delusions in newly admitted residents of nursing homes. The American journal of psychiatry. 1990;147(3):299–302. 10.1176/ajp.147.3.299 . 2309945

[5] Steele C, Rovner B, Chase GA, Folstein M. Psychiatric symptoms and nursing home placement of patients with Alzheimer's disease. The American journal of psychiatry. 1990;147(8):1049–51. 10.1176/ajp.147.8.1049 . 2375439

[6] Kales HC, Gitlin LN, Lyketsos CG, Detroit Expert Panel on A, Management of Neuropsychiatric Symptoms of D. Management of neuropsychiatric symptoms of dementia in clinical settings: recommendations from a multidisciplinary expert panel. Journal of the American Geriatrics Society. 2014;62(4):762–9. 10.1111/jgs.12730 24635665 PMC4146407

[7] Livingston G, Kelly L, Lewis-Holmes E, Baio G, Morris S, Patel N, et al Non-pharmacological interventions for agitation in dementia: systematic review of randomised controlled trials. The British journal of psychiatry: the journal of mental science. 2014;205(6):436–42. 10.1192/bjp.bp.113.141119 . 25452601

[8] Leung P, Orrell M, Orgeta V. Social support group interventions in people with dementia and mild cognitive impairment: a systematic review of the literature. International journal of geriatric psychiatry. 2015;30(1):1–9. 10.1002/gps.4166 . 24990344

[9] Sarkamo T, Tervaniemi M, Laitinen S, Numminen A, Kurki M, Johnson JK, et al Cognitive, emotional, and social benefits of regular musical activities in early dementia: randomized controlled study. The Gerontologist. 2014;54(4):634–50. 10.1093/geront/gnt100 . 24009169

[10] Raglio A, Bellelli G, Mazzola P, Bellandi D, Giovagnoli AR, Farina E, et al Music, music therapy and dementia: a review of literature and the recommendations of the Italian Psychogeriatric Association. Maturitas. 2012;72(4):305–10. 10.1016/j.maturitas.2012.05.016 . 22743206

[11] Stanley MA, Calleo J, Bush AL, Wilson N, Snow AL, Kraus-Schuman C, et al The peaceful mind program: a pilot test of a cognitive-behavioral therapy-based intervention for anxious patients with dementia. The American journal of geriatric psychiatry: official journal of the American Association for Geriatric Psychiatry. 2013;21(7):696–708. 10.1016/j.jagp.2013.01.007 23567399 PMC3411894

[12] Feldman H, Gauthier S, Hecker J, Vellas B, Subbiah P, Whalen E, et al A 24-week, randomized, double-blind study of donepezil in moderate to severe Alzheimer's disease. Neurology. 2001;57(4):613–20. . 11524468 10.1212/wnl.57.4.613

[13] Herrmann N, Rabheru K, Wang J, Binder C. Galantamine treatment of problematic behavior in Alzheimer disease: post-hoc analysis of pooled data from three large trials. The American journal of geriatric psychiatry: official journal of the American Association for Geriatric Psychiatry. 2005;13(6):527–34. 10.1176/appi.ajgp.13.6.527 . 15956273

[14] Bullock R, Bergman H, Touchon J, Gambina G, He Y, Nagel J, et al Effect of age on response to rivastigmine or donepezil in patients with Alzheimer's disease. Current medical research and opinion. 2006;22(3):483–94. 10.1185/030079906X89685 . 16574032

[15] Gauthier S, Loft H, Cummings J. Improvement in behavioural symptoms in patients with moderate to severe Alzheimer's disease by memantine: a pooled data analysis. International journal of geriatric psychiatry. 2008;23(5):537–45. 10.1002/gps.1949 . 18058838

[16] McKeith I, Del Ser T, Spano P, Emre M, Wesnes K, Anand R, et al Efficacy of rivastigmine in dementia with Lewy bodies: a randomised, double-blind, placebo-controlled international study. Lancet. 2000;356(9247):2031–6. 10.1016/S0140-6736(00)03399-7 . 11145488

[17] Mori E, Ikeda M, Kosaka K, Donepezil DLBSI. Donepezil for dementia with Lewy bodies: a randomized, placebo-controlled trial. Annals of neurology. 2012;72(1):41–52. 10.1002/ana.23557 22829268 PMC3504981

[18] American Psychiatric Association Work Group on Alzheimer's disease and other dementias. American Psychiatric Association practice guideline for the treatment of patients with Alzheimer's disease and other dementias. Second edition. The American journal of psychiatry. 2007;164(12 Suppl):5–56. . 18340692

[19] Hirono N, Mori E, Tanimukai S, Kazui H, Hashimoto M, Hanihara T, et al Distinctive neurobehavioral features among neurodegenerative dementias. The Journal of neuropsychiatry and clinical neurosciences. 1999;11(4):498–503. 10.1176/jnp.11.4.498 . 10570764

[20] Srikanth S, Nagaraja AV, Ratnavalli E. Neuropsychiatric symptoms in dementia-frequency, relationship to dementia severity and comparison in Alzheimer's disease, vascular dementia and frontotemporal dementia. Journal of the neurological sciences. 2005;236(1–2):43–8. 10.1016/j.jns.2005.04.014 . 15964021

[21] Morris JC. The Clinical Dementia Rating (CDR): current version and scoring rules. Neurology. 1993;43(11):2412–4. . 8232972 10.1212/wnl.43.11.2412-a

[22] Cummings JL, Mega M, Gray K, Rosenberg-Thompson S, Carusi DA, Gornbein J. The Neuropsychiatric Inventory: comprehensive assessment of psychopathology in dementia. Neurology. 1994;44(12):2308–14. . 7991117 10.1212/wnl.44.12.2308

[23] Shigenobu K, Hirono N, Tabushi K, Ikeda M. [Validity and reliability of the Japanese Version of the Neuropsychiatric Inventory-Nursing Home Version (NPI-NH)]. Brain and nerve = Shinkei kenkyu no shinpo. 2008;60(12):1463–9. . 19110758

[24] Mori S, Mori E, Iseki E, Kosaka K. Efficacy and safety of donepezil in patients with dementia with Lewy bodies: preliminary findings from an open-label study. Psychiatry and clinical neurosciences. 2006;60(2):190–5. 10.1111/j.1440-1819.2006.01485.x . 16594943

[25] Kaufer DI, Cummings JL, Christine D, Bray T, Castellon S, Masterman D, et al Assessing the impact of neuropsychiatric symptoms in Alzheimer's disease: the Neuropsychiatric Inventory Caregiver Distress Scale. Journal of the American Geriatrics Society. 1998;46(2):210–5. . 9475452 10.1111/j.1532-5415.1998.tb02542.x

[26] American, Psychiatric, Association. Diagnostic and Statistical Manual on Mental Disorders, ed 3, revised (DSMIII-R). Washington: American Psychiatric Association; 2008.

[27] McKhann G, Drachman D, Folstein M, Katzman R, Price D, Stadlan EM. Clinical diagnosis of Alzheimer's disease: report of the NINCDS-ADRDA Work Group under the auspices of Department of Health and Human Services Task Force on Alzheimer's Disease. Neurology. 1984;34(7):939–44. . 6610841 10.1212/wnl.34.7.939

[28] Roman GC, Tatemichi TK, Erkinjuntti T, Cummings JL, Masdeu JC, Garcia JH, et al Vascular dementia: diagnostic criteria for research studies. Report of the NINDS-AIREN International Workshop. Neurology. 1993;43(2):250–60. . 8094895 10.1212/wnl.43.2.250

[29] Chui HC, Victoroff JI, Margolin D, Jagust W, Shankle R, Katzman R. Criteria for the diagnosis of ischemic vascular dementia proposed by the State of California Alzheimer's Disease Diagnostic and Treatment Centers. Neurology. 1992;42(3 Pt 1):473–80. . 1549205 10.1212/wnl.42.3.473

[30] McKeith IG, Dickson DW, Lowe J, Emre M, O'Brien JT, Feldman H, et al Diagnosis and management of dementia with Lewy bodies: third report of the DLB Consortium. Neurology. 2005;65(12):1863–72. 10.1212/01.wnl.0000187889.17253.b1 . 16237129

[31] Neary D, Snowden JS, Gustafson L, Passant U, Stuss D, Black S, et al Frontotemporal lobar degeneration: a consensus on clinical diagnostic criteria. Neurology. 1998;51(6):1546–54. . 9855500 10.1212/wnl.51.6.1546

[32] Johnson DK, Watts AS, Chapin BA, Anderson R, Burns JM. Neuropsychiatric profiles in dementia. Alzheimer disease and associated disorders. 2011;25(4):326–32. 10.1097/WAD.0b013e31820d89b6 22086220 PMC3218373

[33] Hashimoto M, Yatabe Y, Ishikawa T, Fukuhara R, Kaneda K, Honda K, et al Relationship between Dementia Severity and Behavioral and Psychological Symptoms of Dementia in Dementia with Lewy Bodies and Alzheimer's Disease Patients. Dementia and geriatric cognitive disorders extra. 2015;5(2):244–52. 10.1159/000381800 26195980 PMC4483492

[34] Chow TW, Fridhandler JD, Binns MA, Lee A, Merrilees J, Rosen HJ, et al Trajectories of behavioral disturbance in dementia. J Alzheimers Dis. 2012;31(1):143–9. 10.3233/JAD-2012-111916 22531424 PMC4309273

[35] Ikejima C, Hisanaga A, Meguro K, Yamada T, Ouma S, Kawamuro Y, et al Multicentre population-based dementia prevalence survey in Japan: a preliminary report. Psychogeriatrics: the official journal of the Japanese Psychogeriatric Society. 2012;12(2):120–3. 10.1111/j.1479-8301.2012.00415.x . 22712646

[36] Vetter PH, Krauss S, Steiner O, Kropp P, Moller WD, Moises HW, et al Vascular dementia versus dementia of Alzheimer's type: do they have differential effects on caregivers' burden? J Gerontol B Psychol Sci Soc Sci. 1999;54(2):S93–8. . 10097779 10.1093/geronb/54b.2.s93

[37] Matsui Y, Tanizaki Y, Arima H, Yonemoto K, Doi Y, Ninomiya T, et al Incidence and survival of dementia in a general population of Japanese elderly: the Hisayama study. Journal of neurology, neurosurgery, and psychiatry. 2009;80(4):366–70. 10.1136/jnnp.2008.155481 . 18977814

[38] Piguet O. Eating disturbance in behavioural-variant frontotemporal dementia. Journal of molecular neuroscience: MN. 2011;45(3):589–93. 10.1007/s12031-011-9547-x . 21584651

[39] Lerner AJ, Koss E, Patterson MB, Ownby RL, Hedera P, Friedland RP, et al Concomitants of visual hallucinations in Alzheimer's disease. Neurology. 1994;44(3 Pt 1):523–7. . 8145925 10.1212/wnl.44.3_part_1.523

[40] Quaranta D, Vita MG, Bizzarro A, Masullo C, Piccininni C, Gainotti G, et al Cognitive and behavioral determinants of psychotic symptoms in Alzheimer's disease. Dementia and geriatric cognitive disorders. 2015;39(3–4):194–206. 10.1159/000369161 . 25572669

[41] Landqvist Waldo M, Gustafson L, Passant U, Englund E. Psychotic symptoms in frontotemporal dementia: a diagnostic dilemma? International psychogeriatrics / IPA. 2015;27(4):531–9. 10.1017/S1041610214002580 25486967 PMC4413855

[42] Rosen HJ, Allison SC, Schauer GF, Gorno-Tempini ML, Weiner MW, Miller BL. Neuroanatomical correlates of behavioural disorders in dementia. Brain: a journal of neurology. 2005;128(Pt 11):2612–25. 10.1093/brain/awh628 16195246 PMC1820861

