# Supplementary material for: Differences of Behavioral and Psychological Symptoms of Dementia in Disease Severity in Four Major Dementias
Source: PLoS One. 2016 Aug 18;11(8):e0161092. doi: 10.1371/journal.pone.0161092 (PMC4990196; doi:10.1371/journal.pone.0161092)

S1 File.

Charts for BPSD in Alzheimer’s disease by disease severity

Delusions

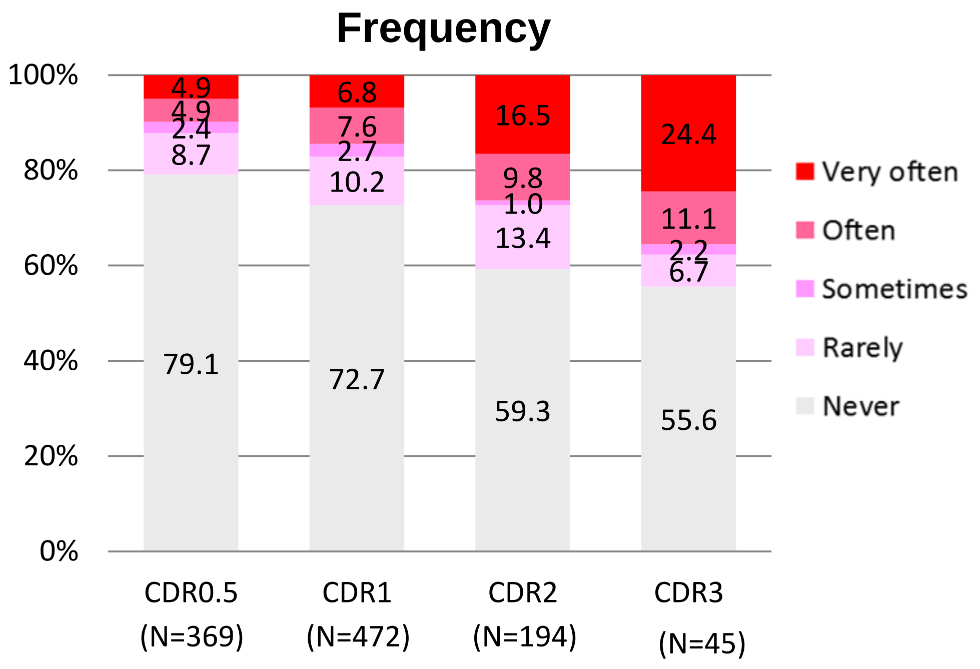
Frequency
100%
80%
60%
40%
20%
0%
4.9
4.9
2.4
8.7
79.1
6.8
7.6
2.7
10.2
72.7
16.5
9.8
1.0
13.4
59.3
24.4
11.1
2.2
6.7
55.6
CDR0.5 (N=369)
CDR1 (N=472)
CDR2 (N=194)
CDR3 (N=45)
Very often
Often
Sometimes
Rarely
Never

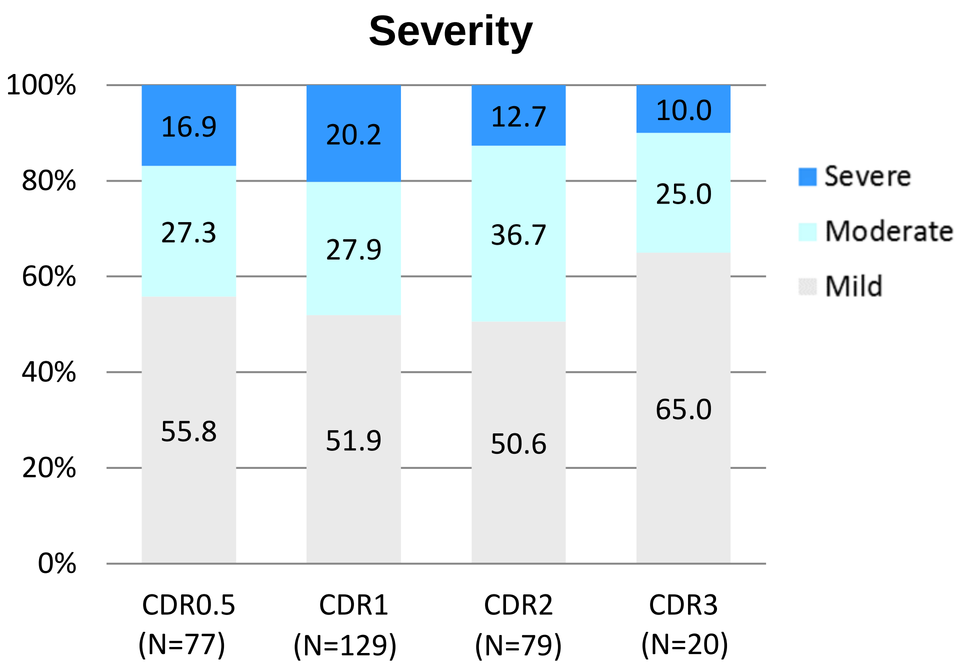
Severity
100%
80%
60%
40%
20%
0%
16.9
27.3
55.8
20.2
27.9
51.9
12.7
36.7
50.6
10.0
25.0
65.0
CDR0.5 (N=77)
CDR1 (N=129)
CDR2 (N=79)
CDR3 (N=20)
Severe
Moderate
Mild

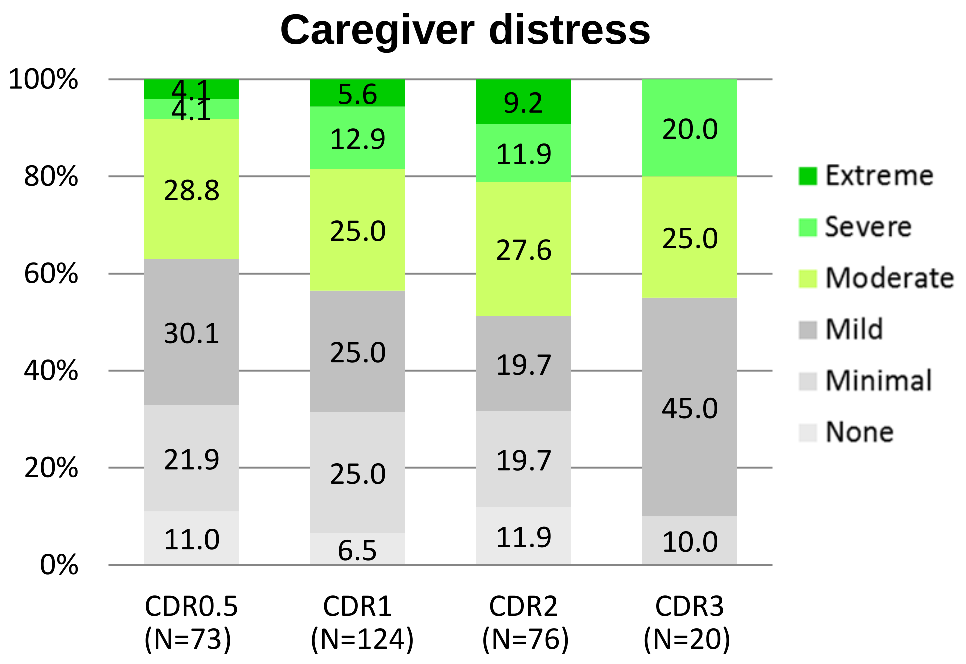
Caregiver distress
100%
80%
60%
40%
20%
0%
4.1
4.1
28.8
30.1
21.9
11.0
5.6
12.9
25.0
25.0
25.0
6.5
9.2
11.9
27.6
19.7
19.7
11.9
20.0
25.0
45.0
10.0
CDR0.5 (N=73)
CDR1 (N=124)
CDR2 (N=76)
CDR3 (N=20)
Extreme
Severe
Moderate
Mild
Minimal
None

Hallucinations

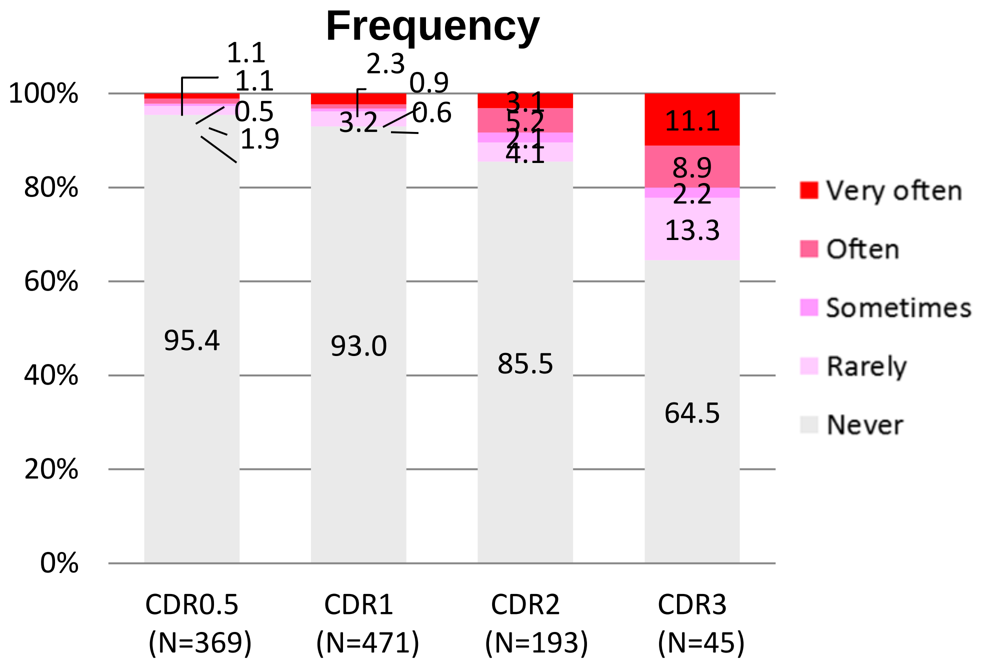

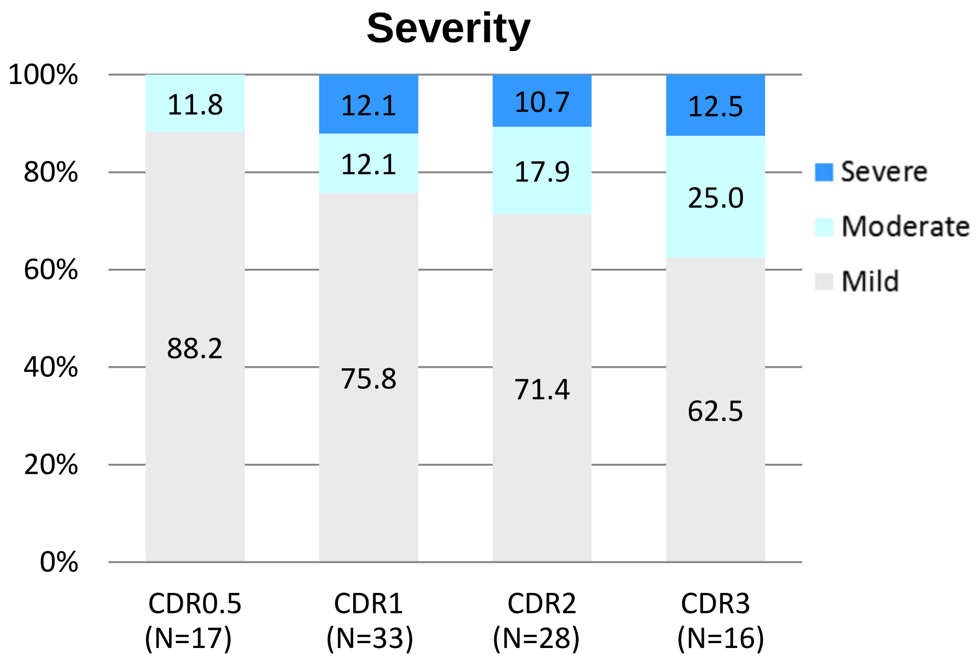

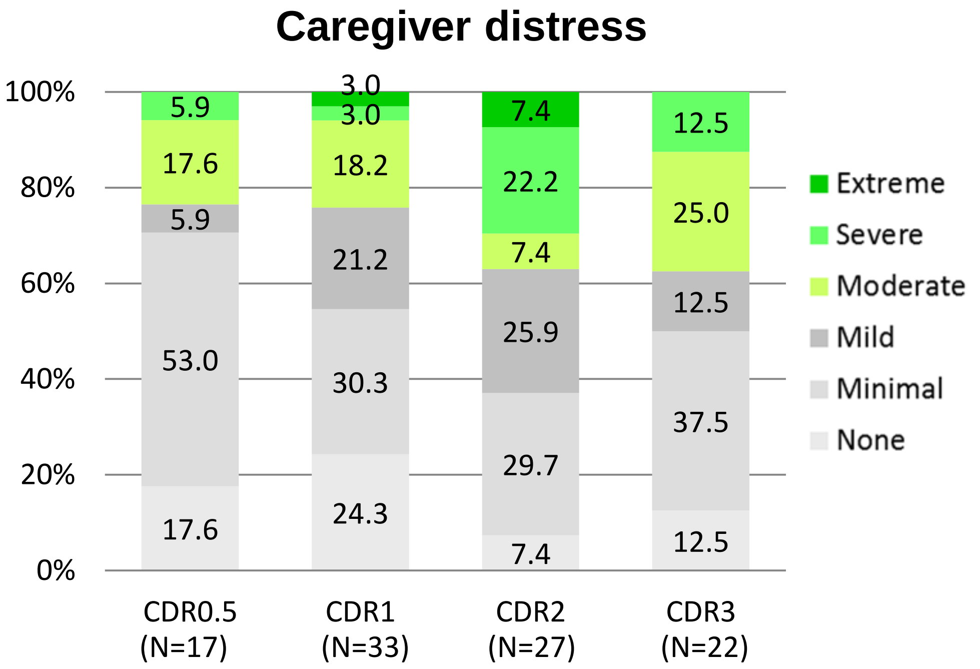

Agitation

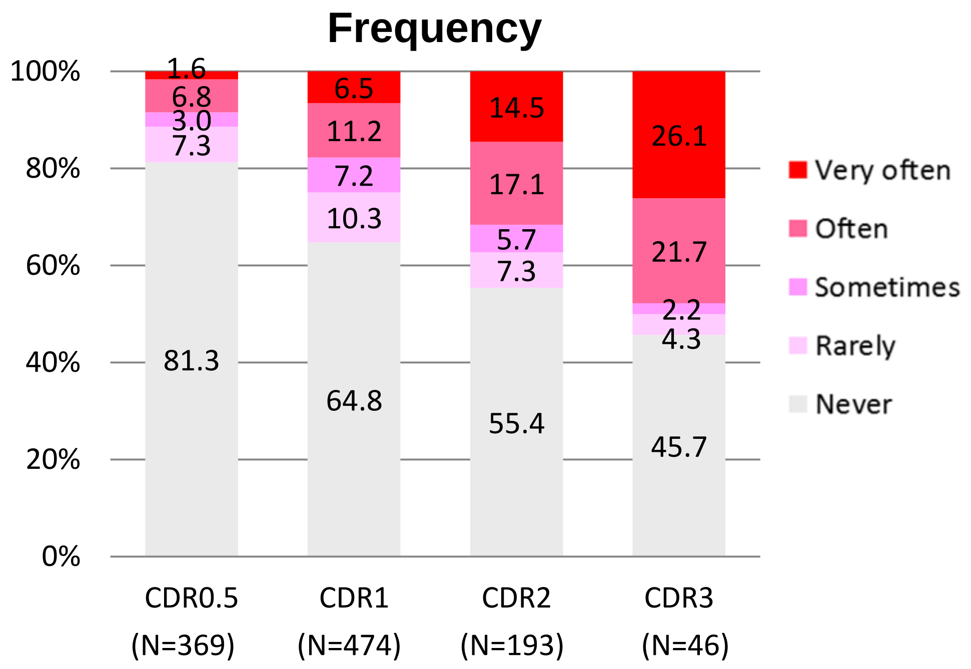
Frequency
100%
80%
60%
40%
20%
0%
1.6
6.8
3.0
7.3
81.3
6.5
11.2
7.2
10.3
64.8
14.5
17.1
5.7
7.3
55.4
26.1
21.7
2.2
4.3
45.7
CDR0.5 (N=369)
CDR1 (N=474)
CDR2 (N=193)
CDR3 (N=46)
Very often
Often
Sometimes
Rarely
Never

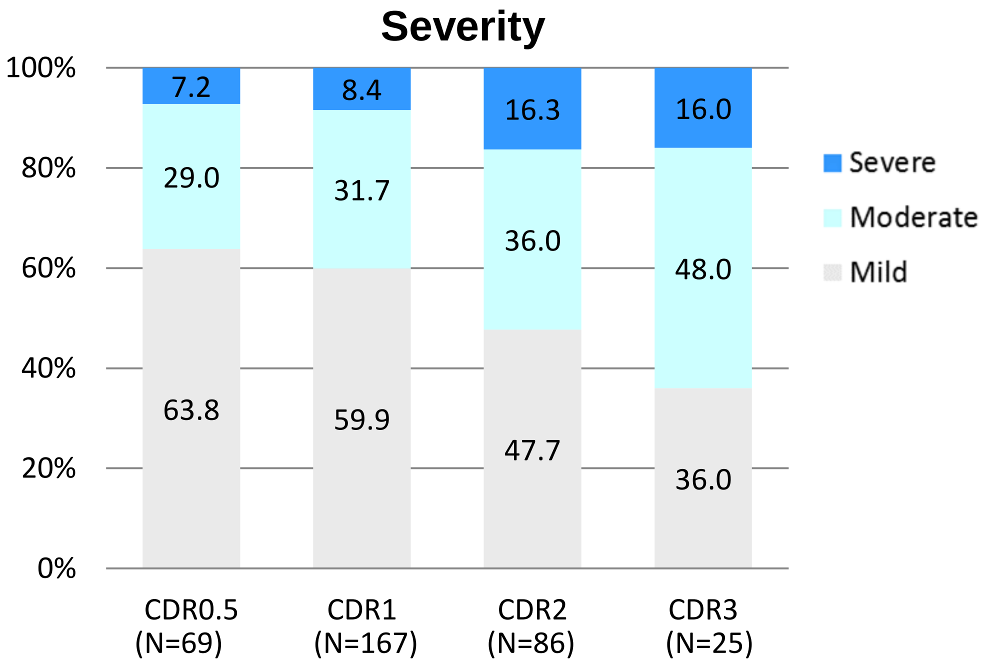
Severity
100%
80%
60%
40%
20%
0%
7.2
29.0
63.8
8.4
31.7
59.9
16.3
36.0
47.7
16.0
48.0
36.0
CDR0.5 (N=69)
CDR1 (N=167)
CDR2 (N=86)
CDR3 (N=25)
Severe
Moderate
Mild

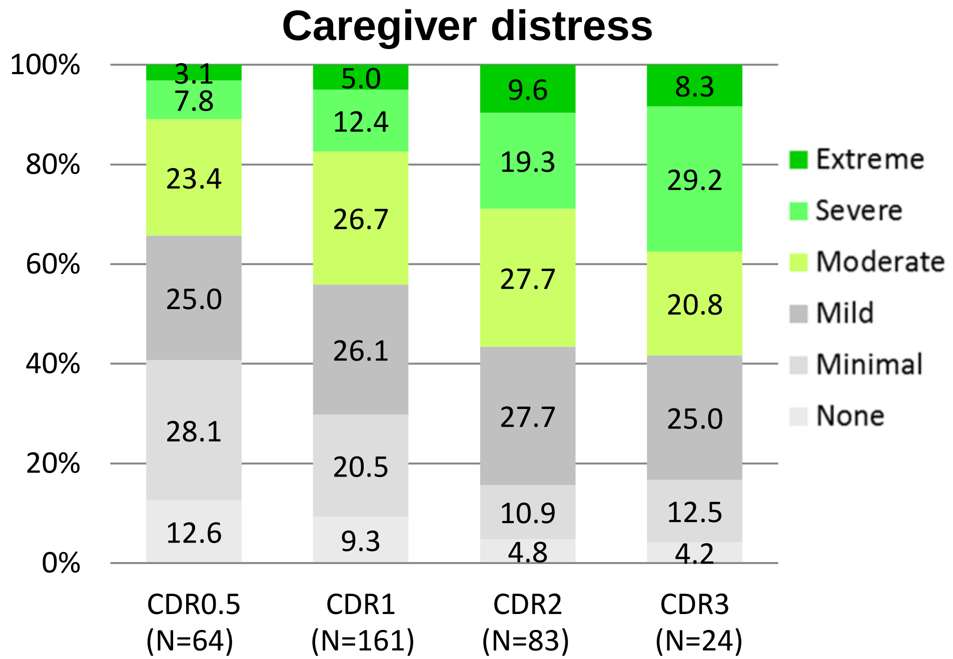
Caregiver distress
100%
80%
60%
40%
20%
0%
3.1
7.8
23.4
25.0
28.1
12.6
5.0
12.4
26.7
26.1
20.5
9.3
9.6
19.3
27.7
27.7
10.9
4.8
8.3
29.2
20.8
25.0
12.5
4.2
CDR0.5 (N=64)
CDR1 (N=161)
CDR2 (N=83)
CDR3 (N=24)
Extreme
Severe
Moderate
Mild
Minimal
None

Depression

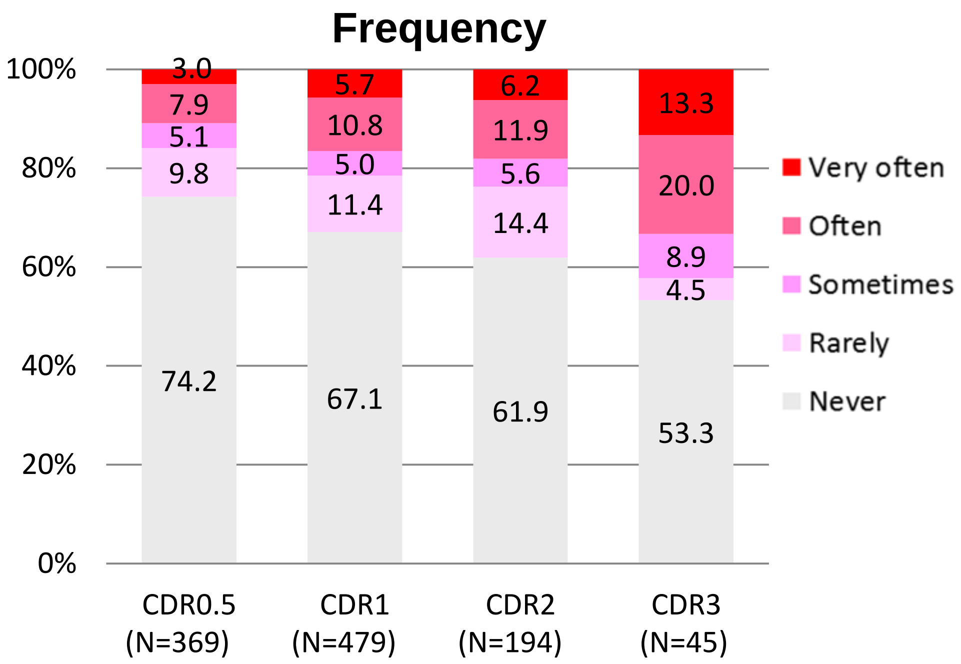
Frequency
100%
80%
60%
40%
20%
0%
3.0
7.9
5.1
9.8
74.2
5.7
10.8
5.0
11.4
67.1
6.2
11.9
5.6
14.4
61.9
13.3
20.0
8.9
4.5
53.3
CDR0.5 (N=369)
CDR1 (N=479)
CDR2 (N=194)
CDR3 (N=45)
Very often
Often
Sometimes
Rarely
Never

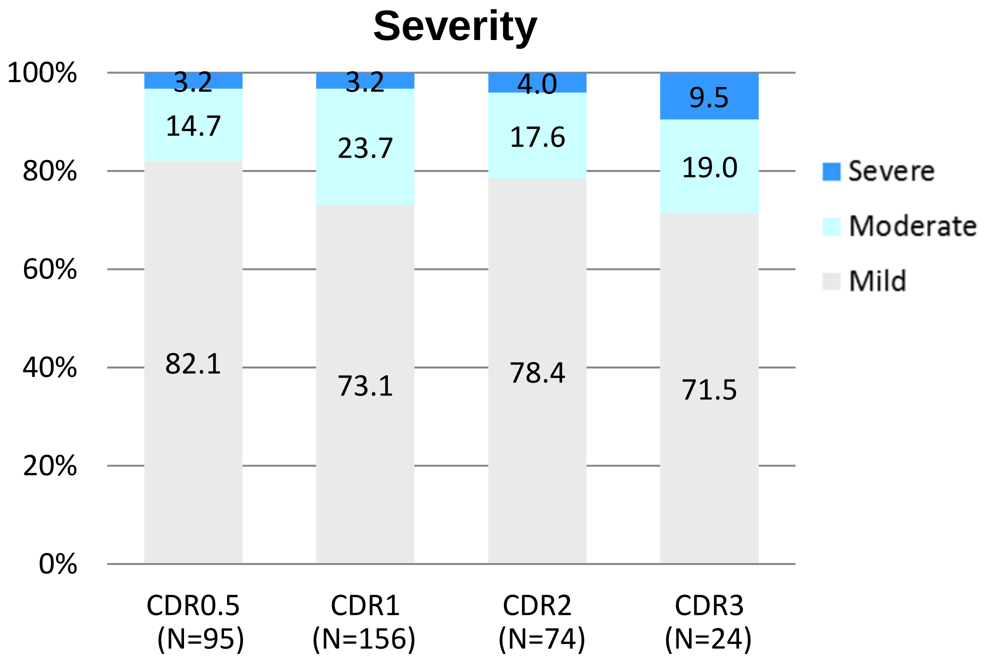
Severity
100%
80%
60%
40%
20%
0%
3.2
14.7
82.1
3.2
23.7
73.1
4.0
17.6
78.4
9.5
19.0
71.5
CDR0.5 (N=95)
CDR1 (N=156)
CDR2 (N=74)
CDR3 (N=24)
Severe
Moderate
Mild

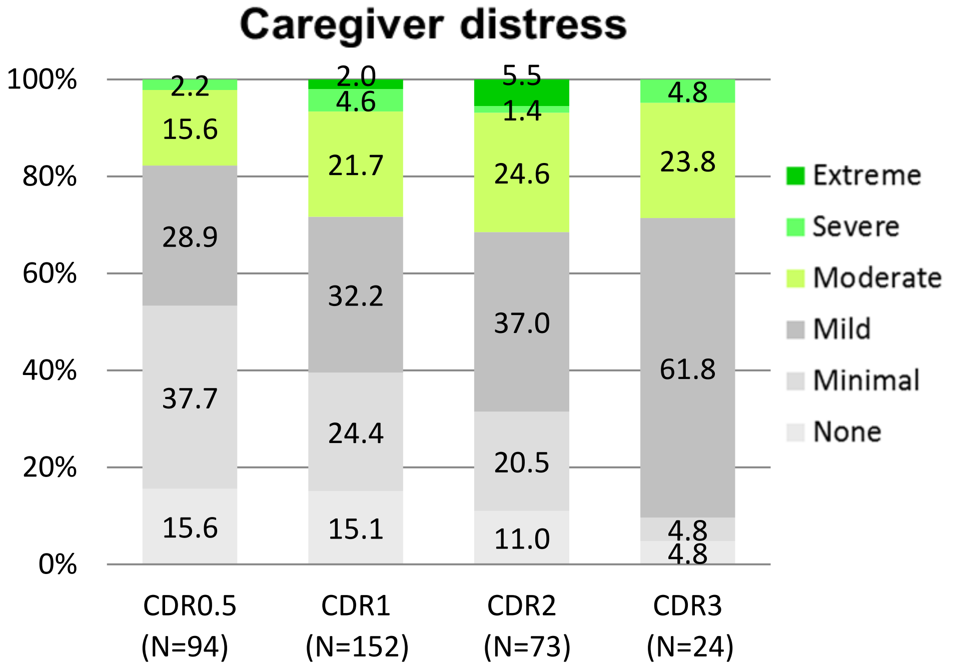
Caregiver distress
100%
80%
60%
40%
20%
0%
2.2
15.6
28.9
37.7
15.6
2.0
4.6
21.7
32.2
24.4
15.1
5.5
1.4
24.6
37.0
20.5
11.0
4.8
23.8
61.8
4.8
4.8
CDR0.5 (N=94)
CDR1 (N=152)
CDR2 (N=73)
CDR3 (N=24)
Extreme
Severe
Moderate
Mild
Minimal
None

Anxiety

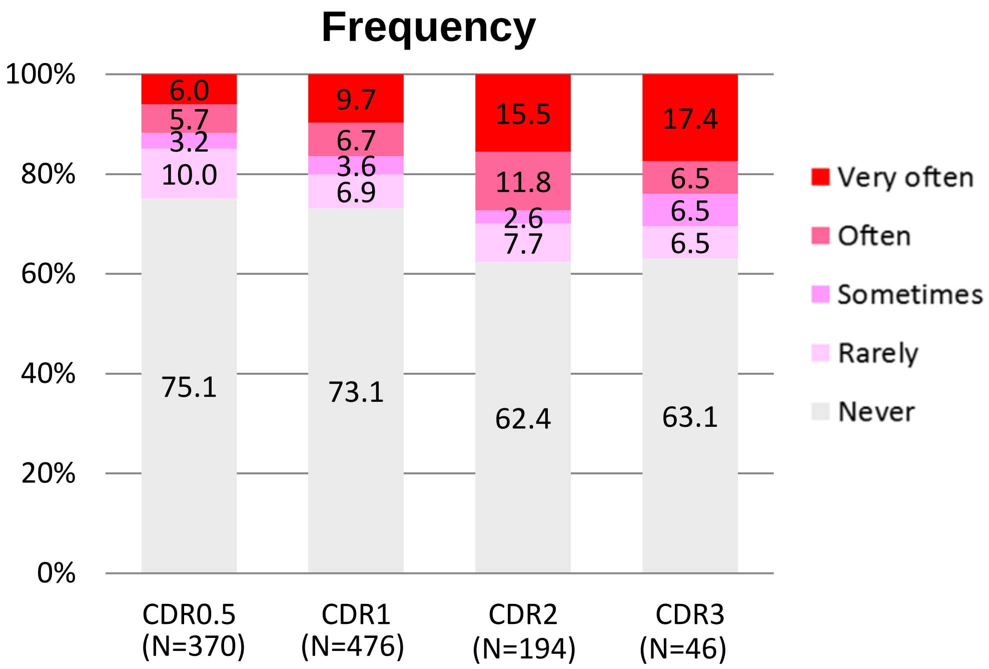
Frequency
100%
80%
60%
40%
20%
0%
6.0
5.7
3.2
10.0
75.1
9.7
6.7
3.6
6.9
73.1
15.5
11.8
2.6
7.7
62.4
17.4
6.5
6.5
6.5
63.1
CDR0.5 (N=370)
CDR1 (N=476)
CDR2 (N=194)
CDR3 (N=46)
Very often
Often
Sometimes
Rarely
Never

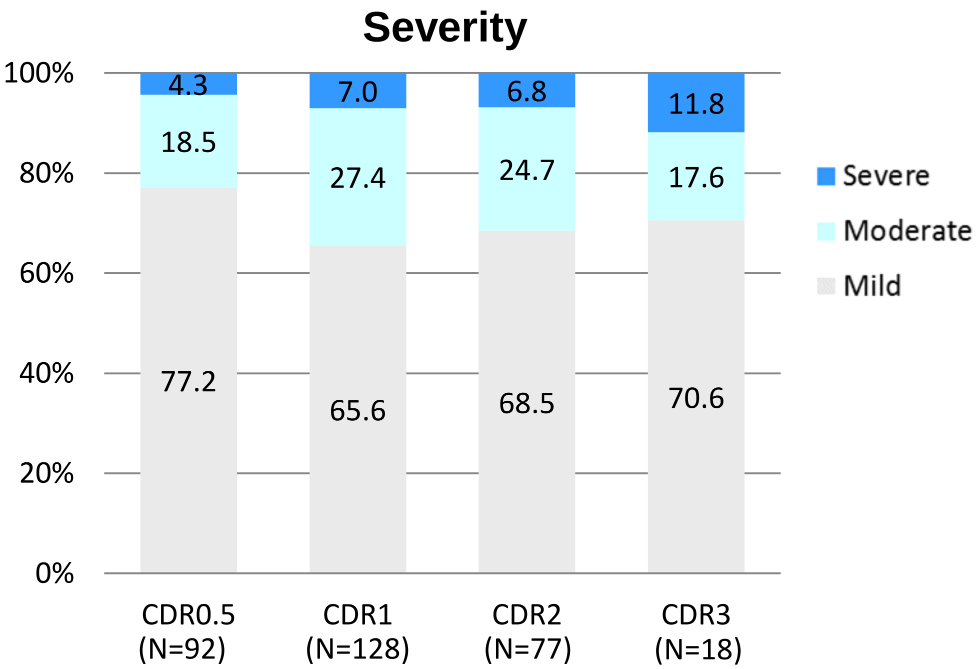
Severity
100%
80%
60%
40%
20%
0%
4.3
18.5
77.2
7.0
27.4
65.6
6.8
24.7
68.5
11.8
17.6
70.6
CDR0.5 (N=92)
CDR1 (N=128)
CDR2 (N=77)
CDR3 (N=18)
Severe
Moderate
Mild

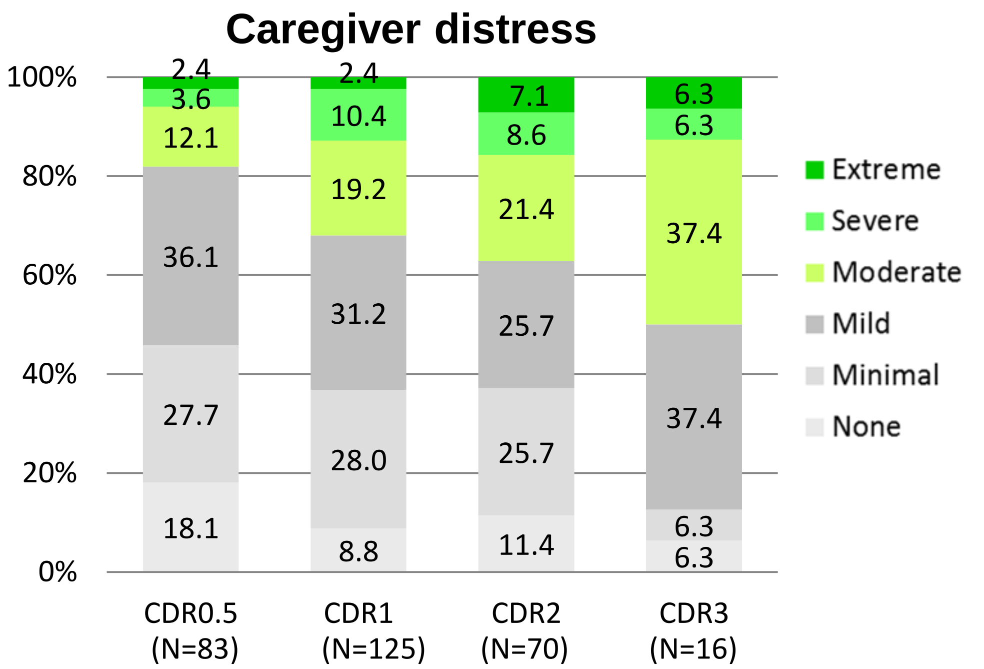
Caregiver distress
100%
80%
60%
40%
20%
0%
2.4
3.6
12.1
36.1
27.7
18.1
2.4
10.4
19.2
31.2
28.0
8.8
7.1
8.6
21.4
25.7
25.7
11.4
6.3
6.3
37.4
37.4
6.3
6.3
CDR0.5 (N=83)
CDR1 (N=125)
CDR2 (N=70)
CDR3 (N=16)
Extreme
Severe
Moderate
Mild
Minimal
None

Euphoria

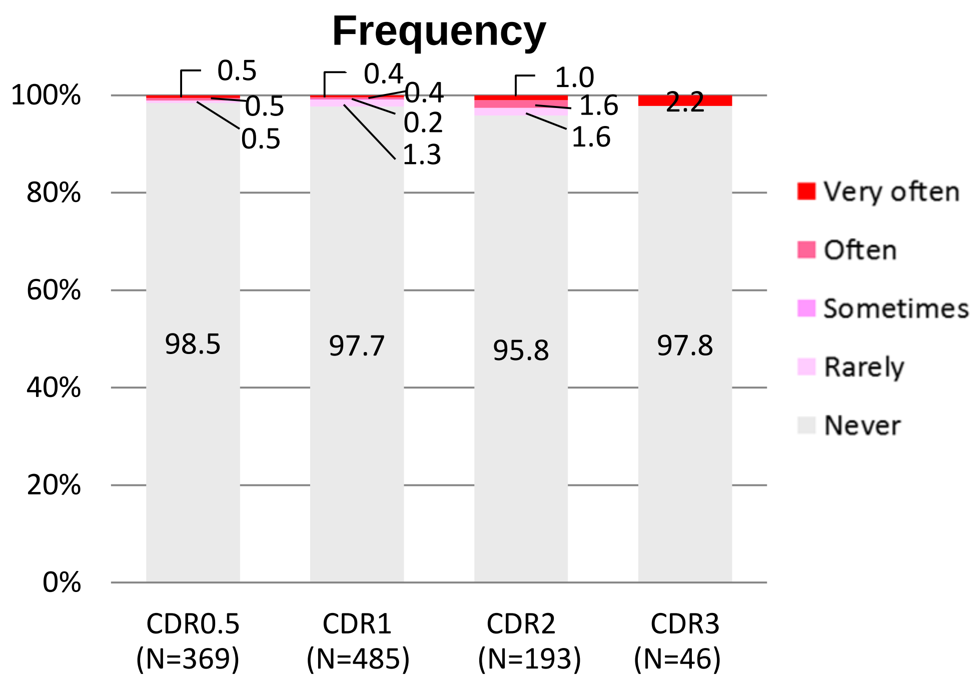

Frequency
100%
80%
60%
40%
20%
0%
0.5
0.5
0.5
98.5
0.4
0.4
0.2
1.3
97.7
1.0
1.6
1.6
95.8
2.2
97.8
CDR0.5 (N=369)
CDR1 (N=485)
CDR2 (N=193)
CDR3 (N=46)
Very often
Often
Sometimes
Rarely
Never

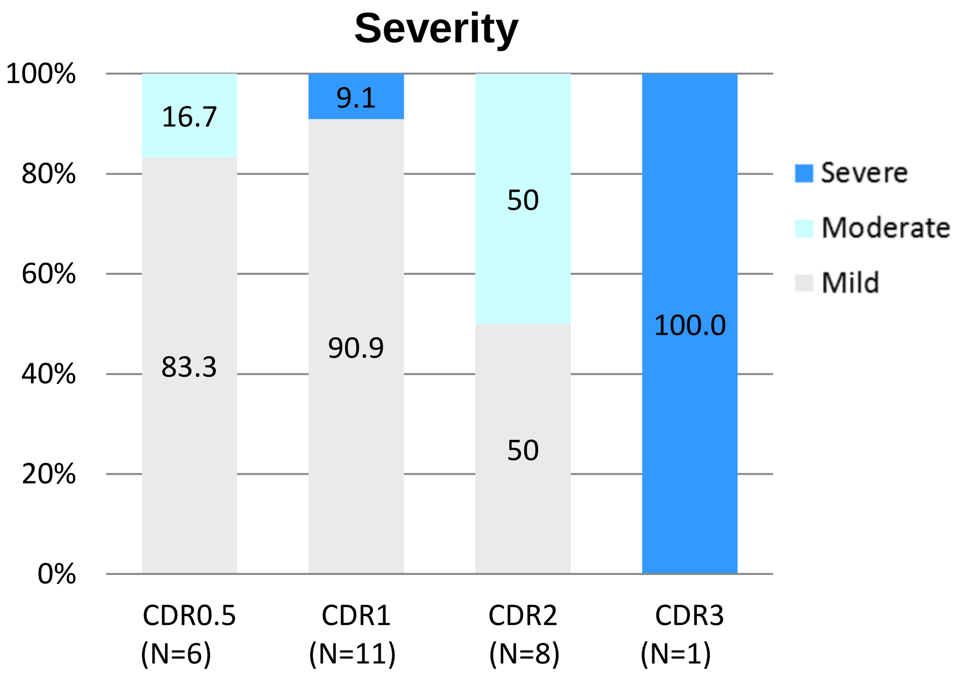

Severity
100%
80%
60%
40%
20%
0%
16.7
83.3
9.1
90.9
50
50
100.0
CDR0.5 (N=6)
CDR1 (N=11)
CDR2 (N=8)
CDR3 (N=1)
Severe
Moderate
Mild

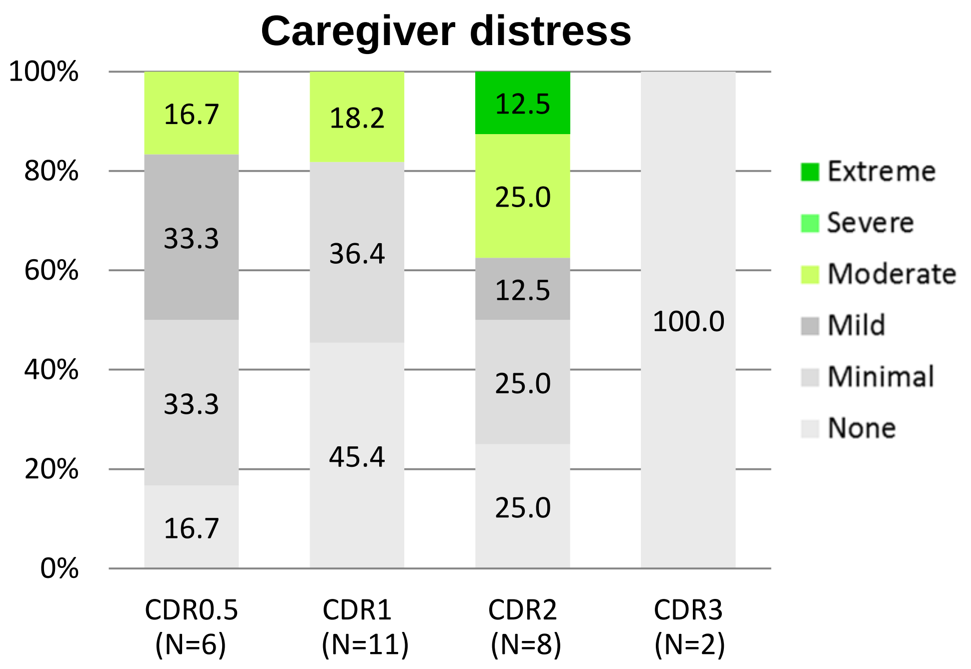

Caregiver distress
100%
80%
60%
40%
20%
0%
16.7
33.3
33.3
16.7
18.2
36.4
45.4
12.5
25.0
12.5
25.0
25.0
100.0
CDR0.5 (N=6)
CDR1 (N=11)
CDR2 (N=8)
CDR3 (N=2)
Extreme
Severe
Moderate
Mild
Minimal
None

Apathy

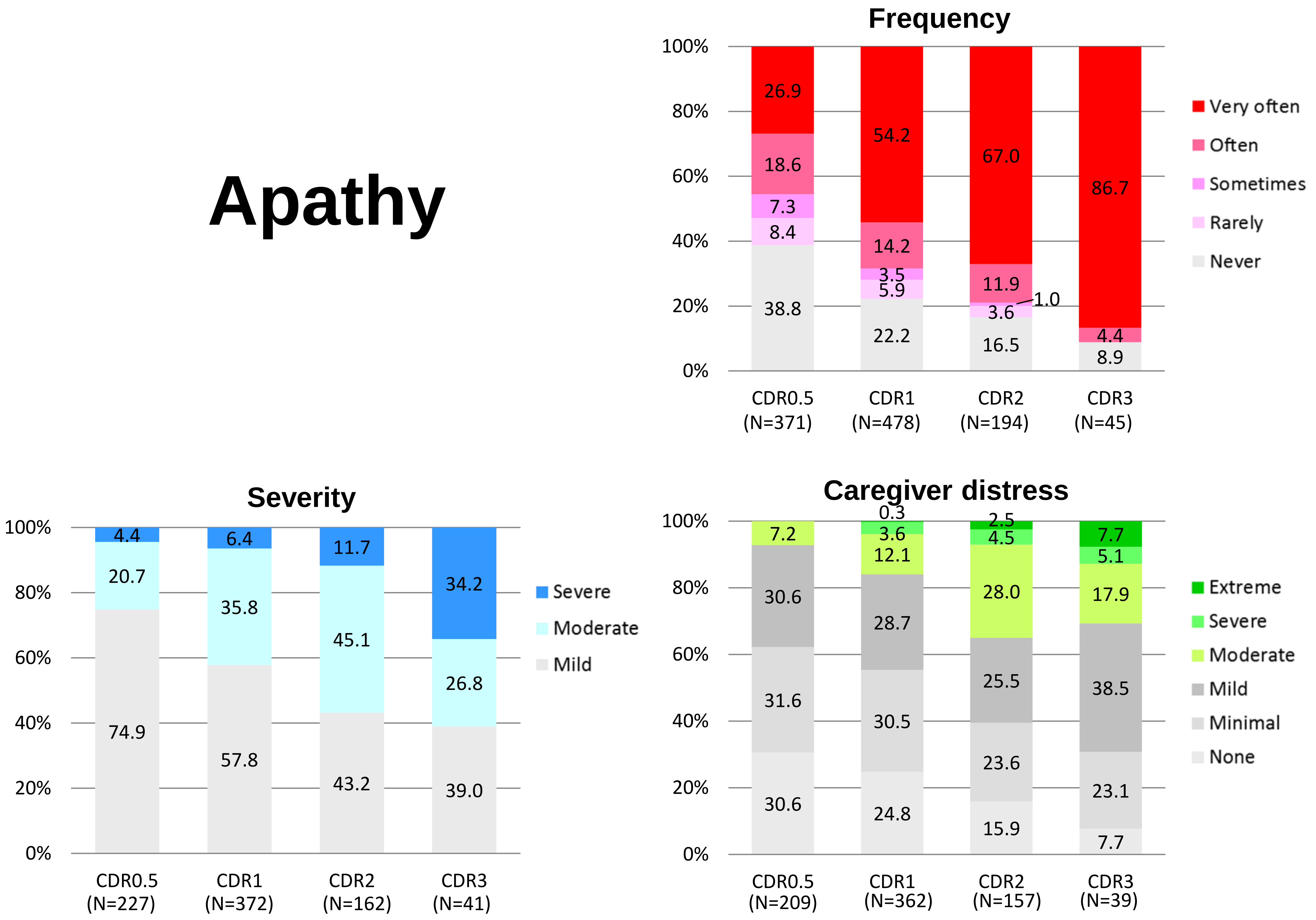
Frequency
Very often
Often
Sometimes
Rarely
Never
26.9
18.6
7.3
8.4
38.8
54.2
14.2
3.5
5.9
22.2
67.0
11.9
1.0
3.6
16.5
86.7
4.4
8.9
CDR0.5 (N=371)
CDR1 (N=478)
CDR2 (N=194)
CDR3 (N=45)
Severity
Severe
Moderate
Mild
4.4
20.7
74.9
6.4
35.8
57.8
11.7
45.1
43.2
34.2
26.8
39.0
CDR0.5 (N=227)
CDR1 (N=372)
CDR2 (N=162)
CDR3 (N=41)
Caregiver distress
Extreme
Severe
Moderate
Mild
Minimal
None
7.2
30.6
31.6
30.6
0.3
3.6
12.1
28.7
30.5
24.8
2.5
4.5
28.0
25.5
23.6
15.9
7.7
5.1
17.9
38.5
23.1
7.7
CDR0.5 (N=209)
CDR1 (N=362)
CDR2 (N=157)
CDR3 (N=39)
0%
20%
40%
60%
80%
100%

Disinhibition

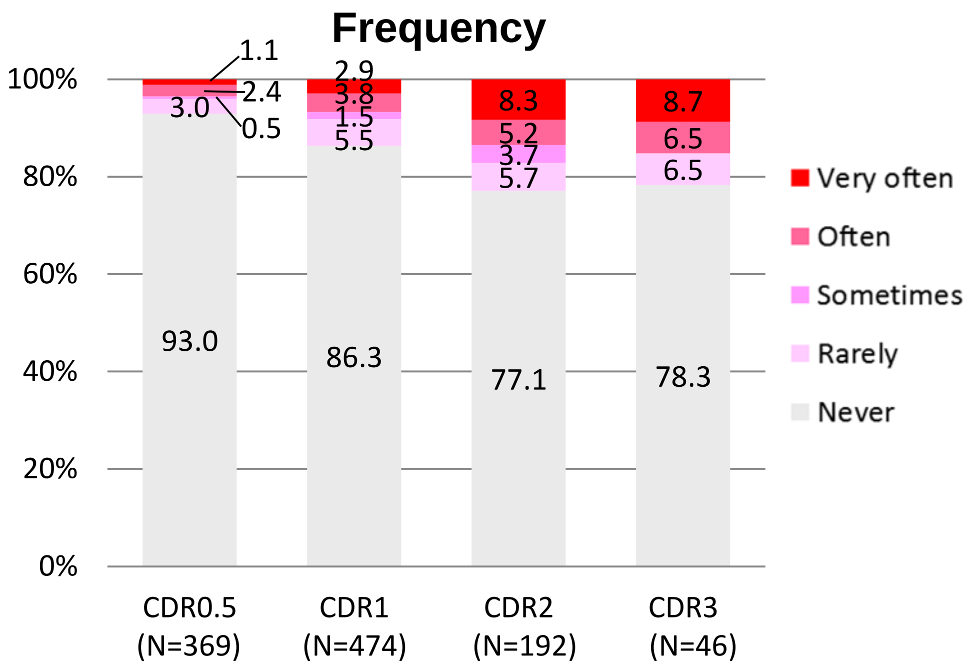

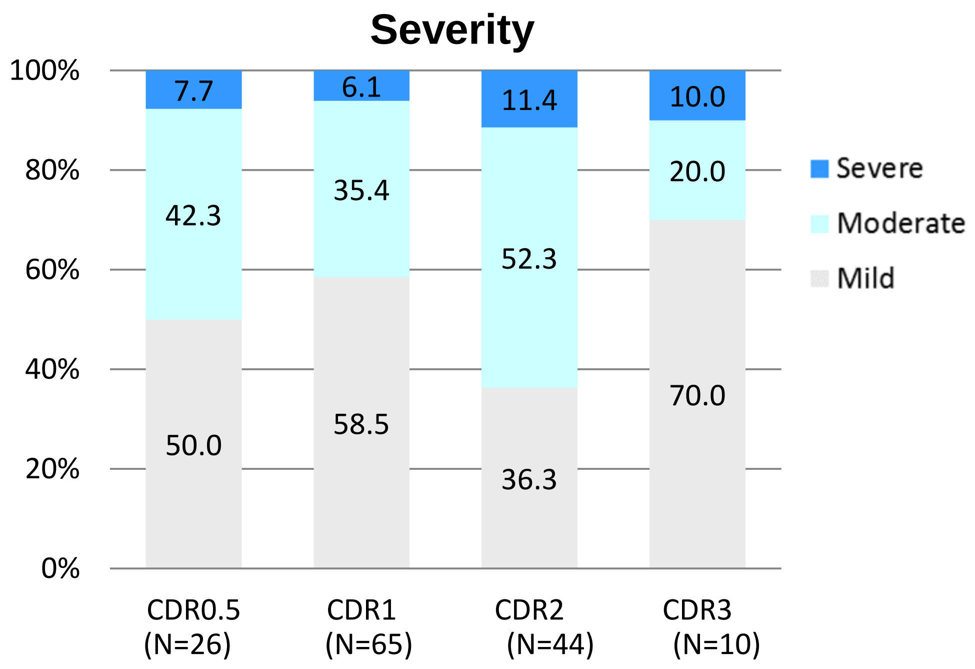

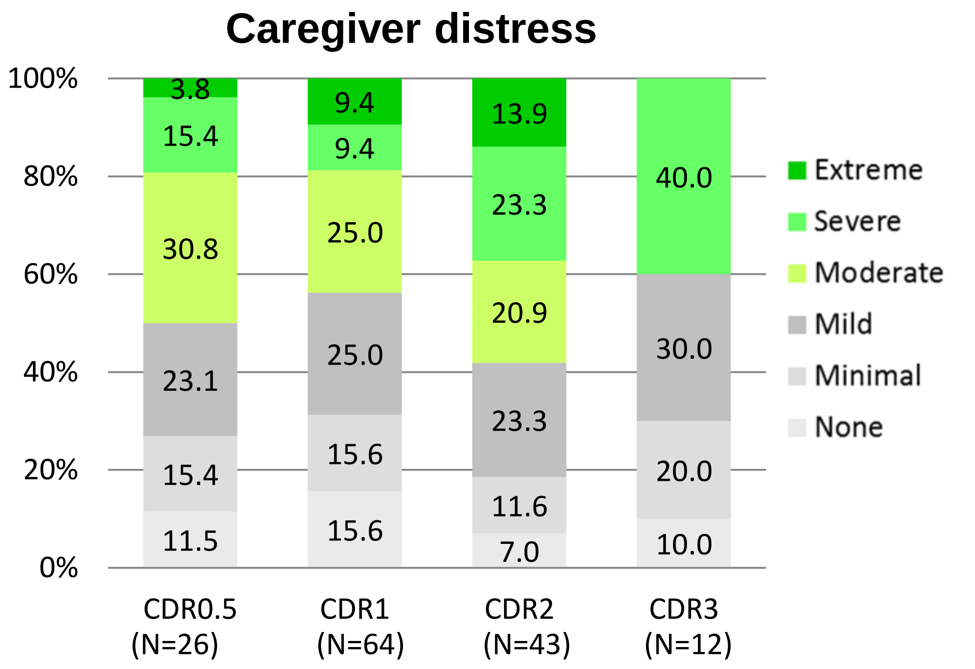

Irritability

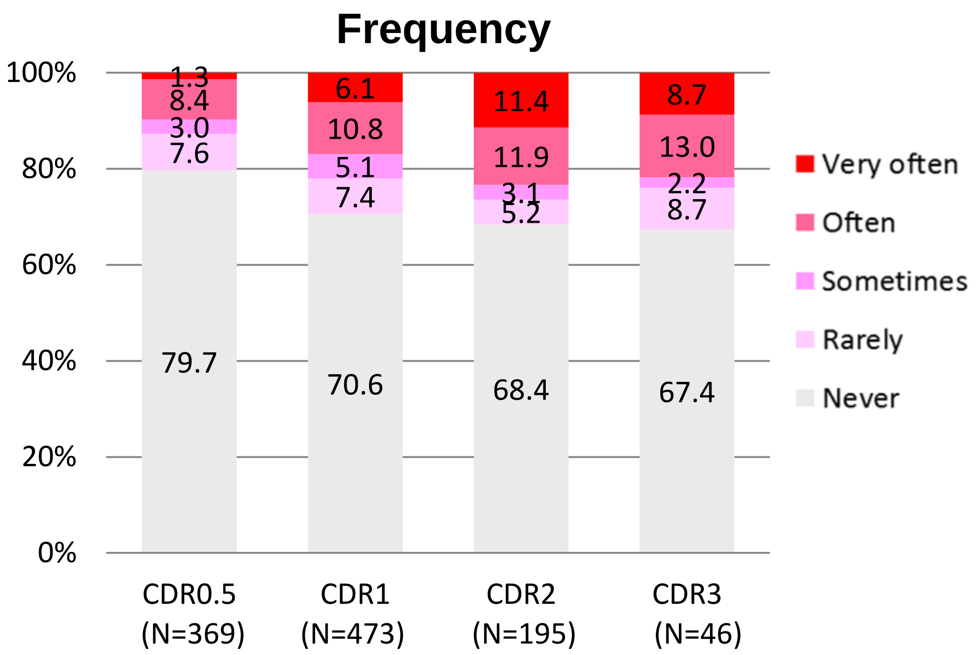

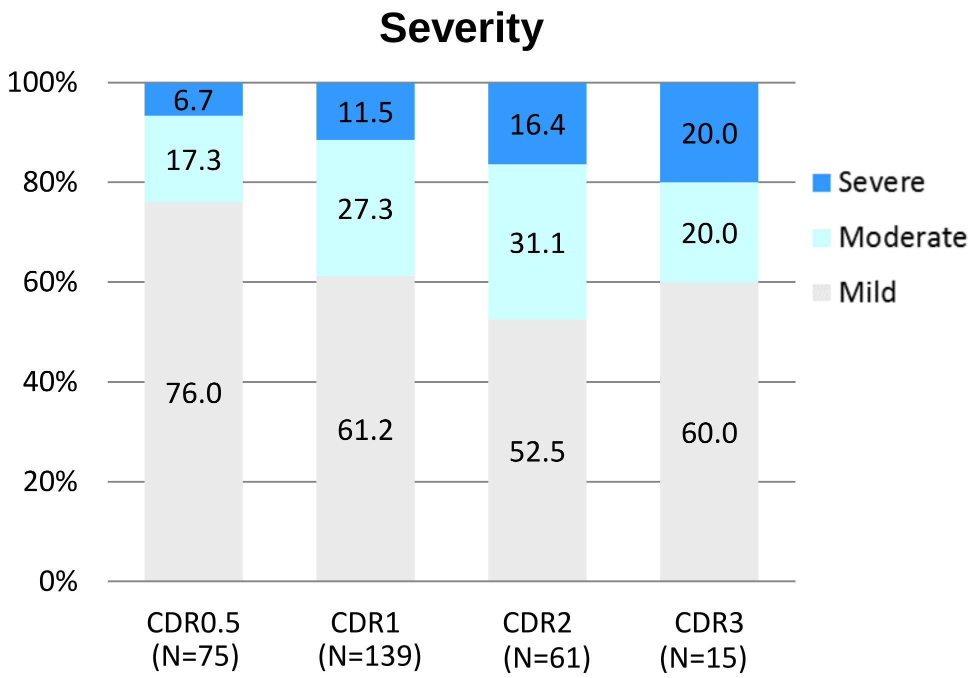

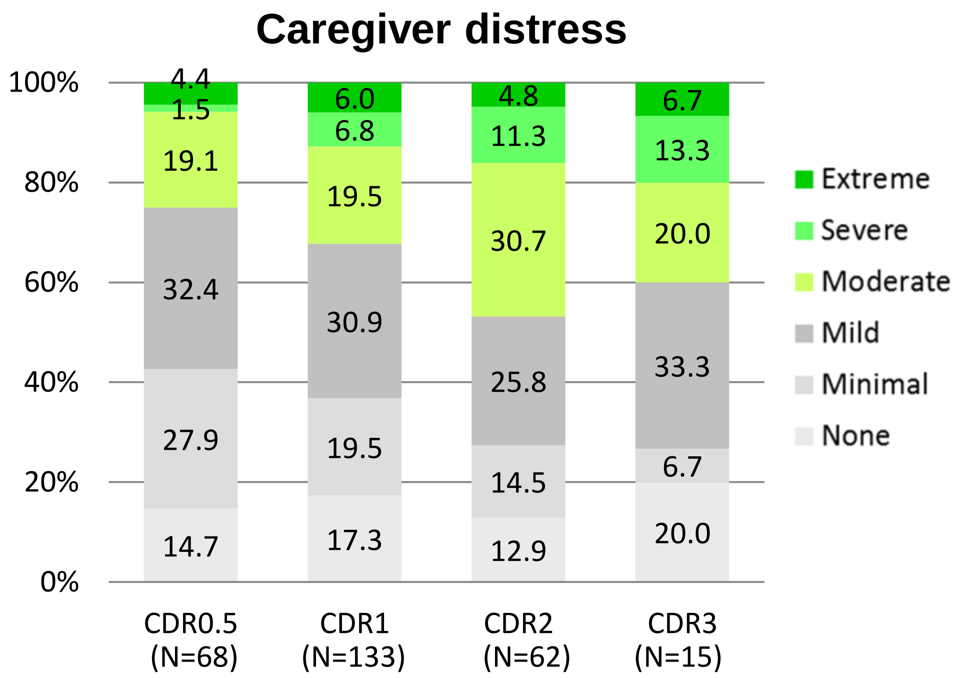

Aberrant motor behavior

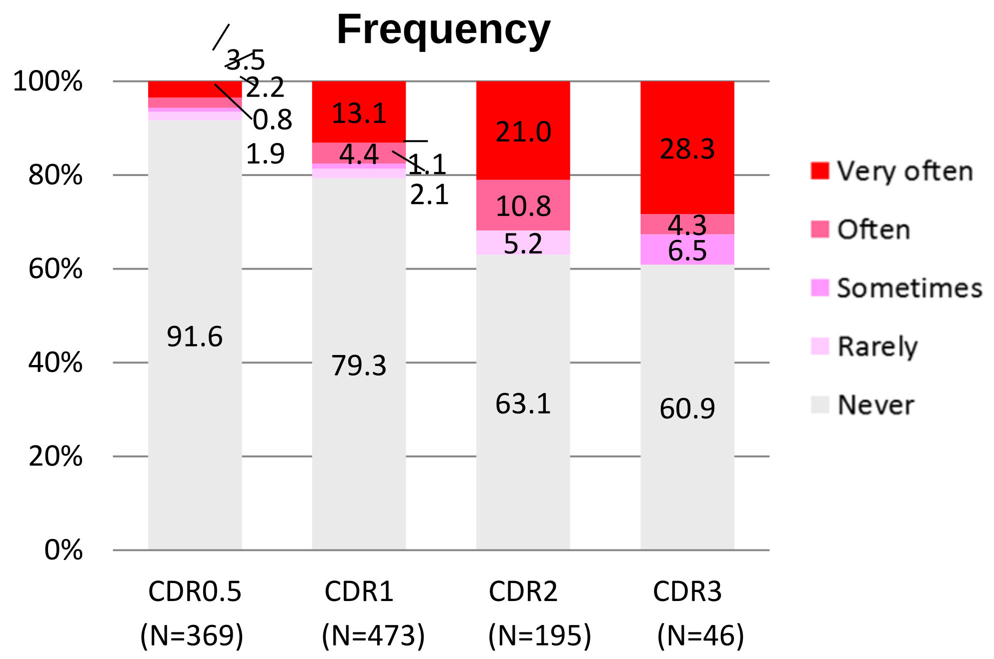

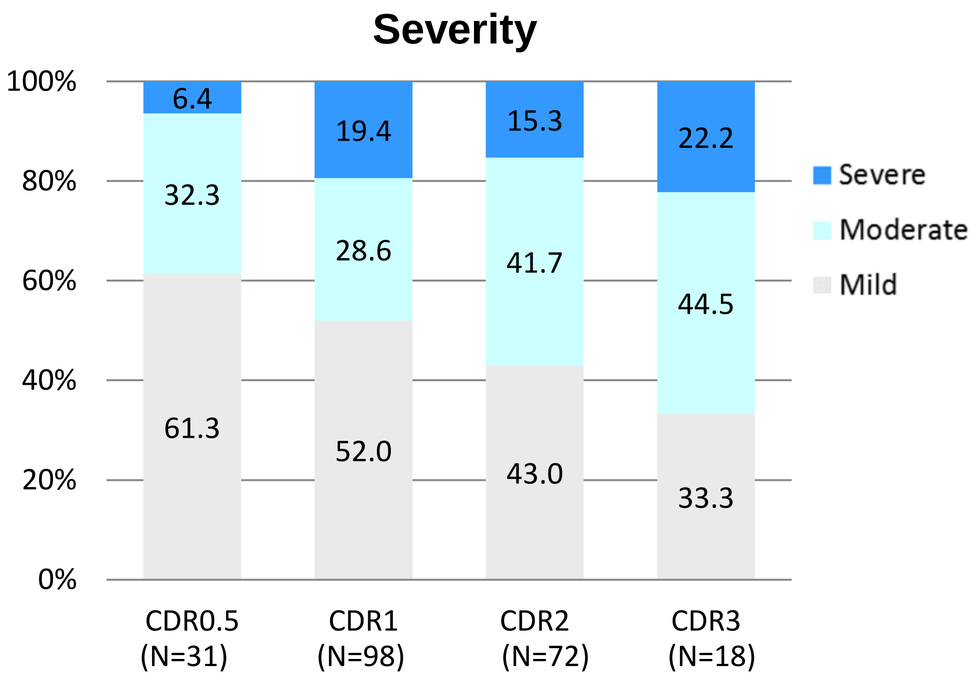

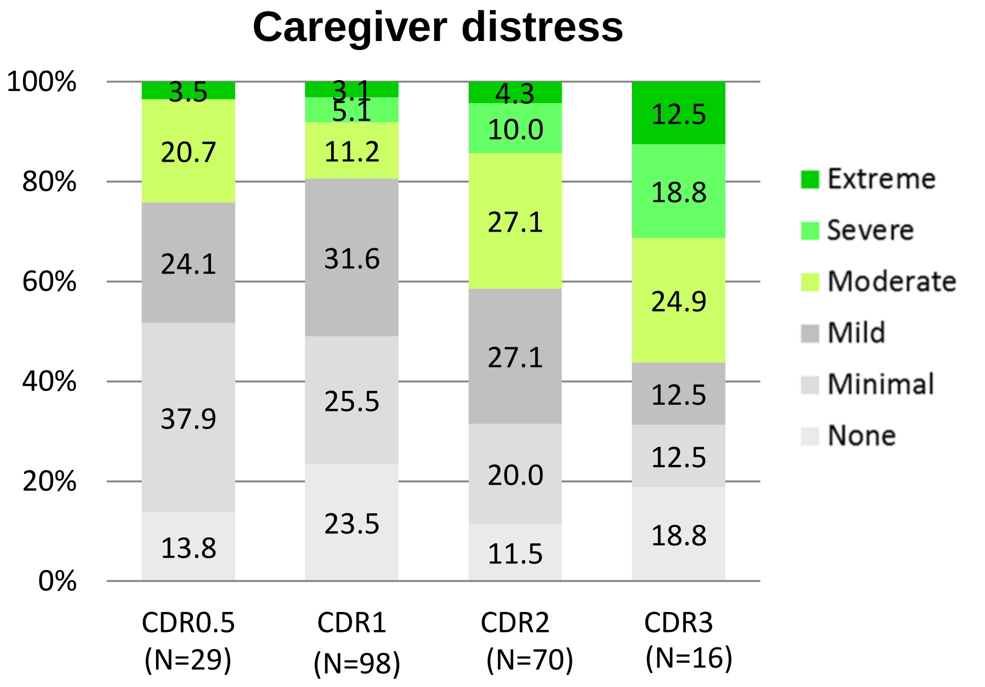

Sleep disturbances

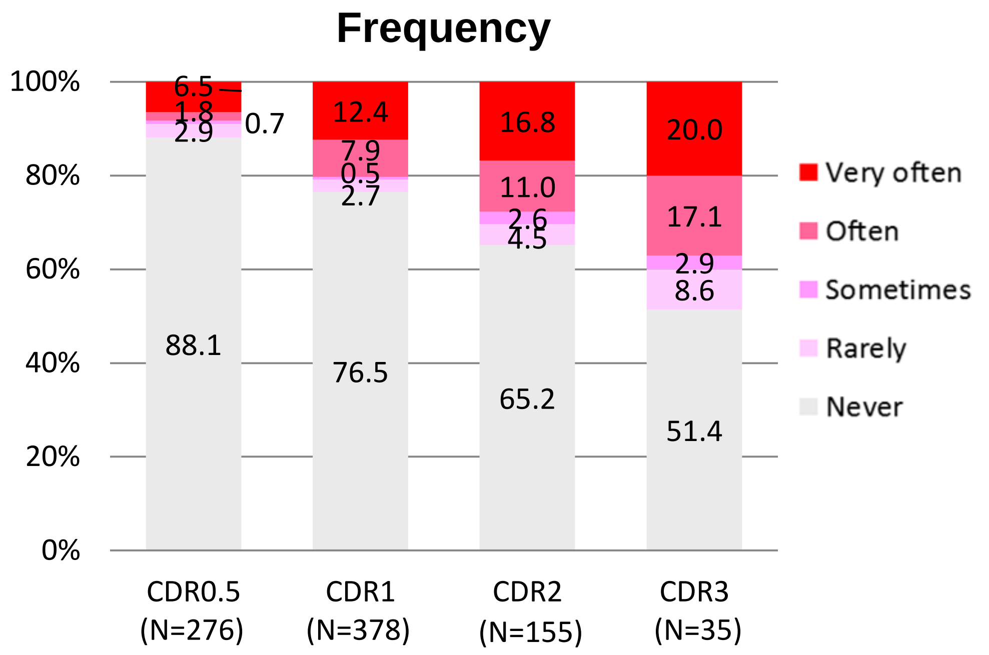

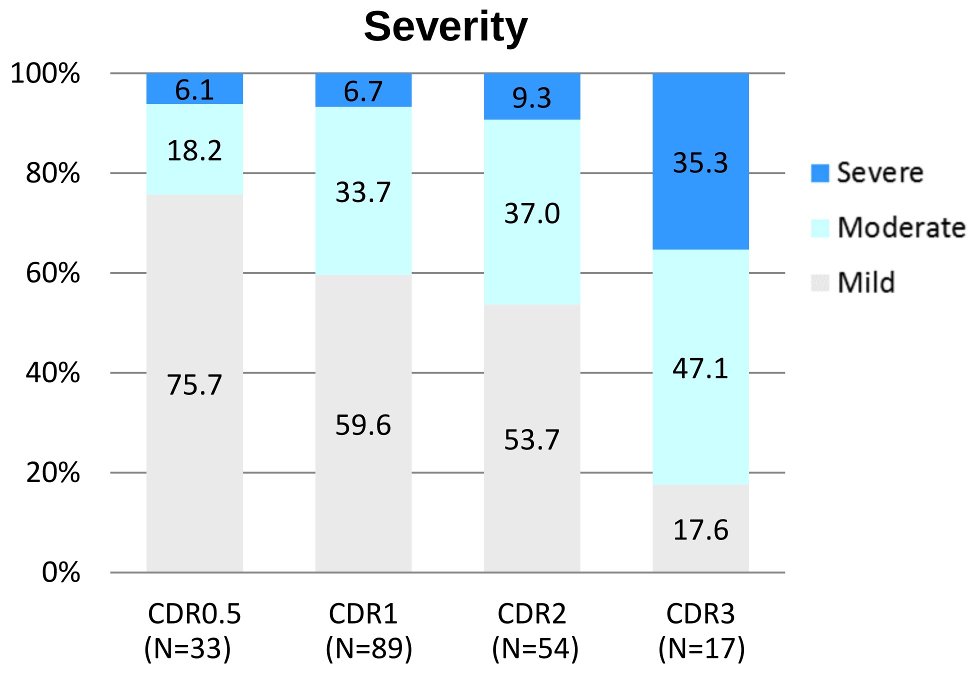

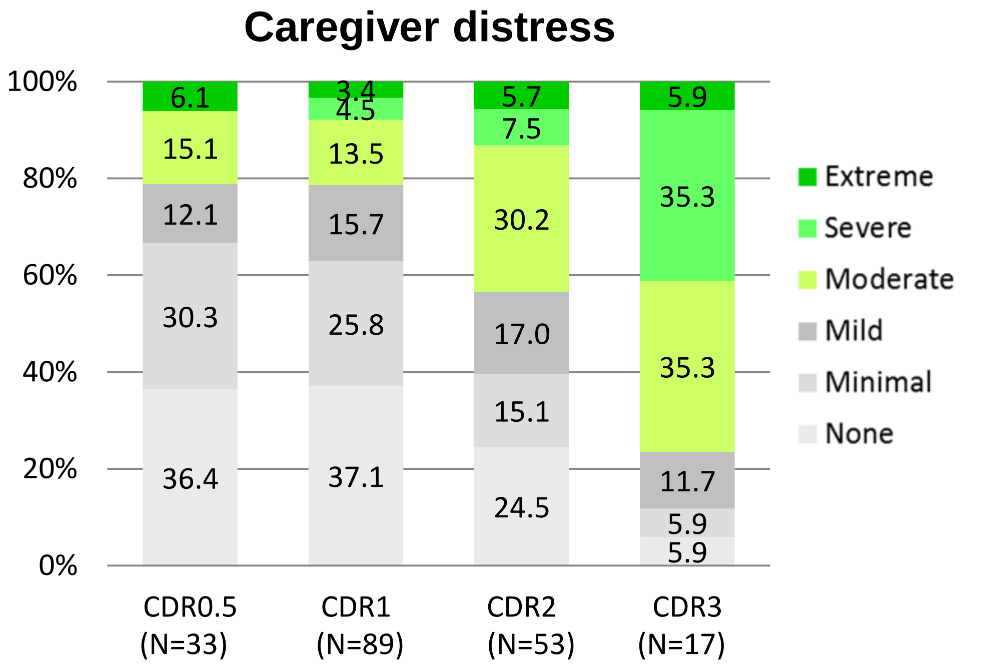

Eating abnormalities

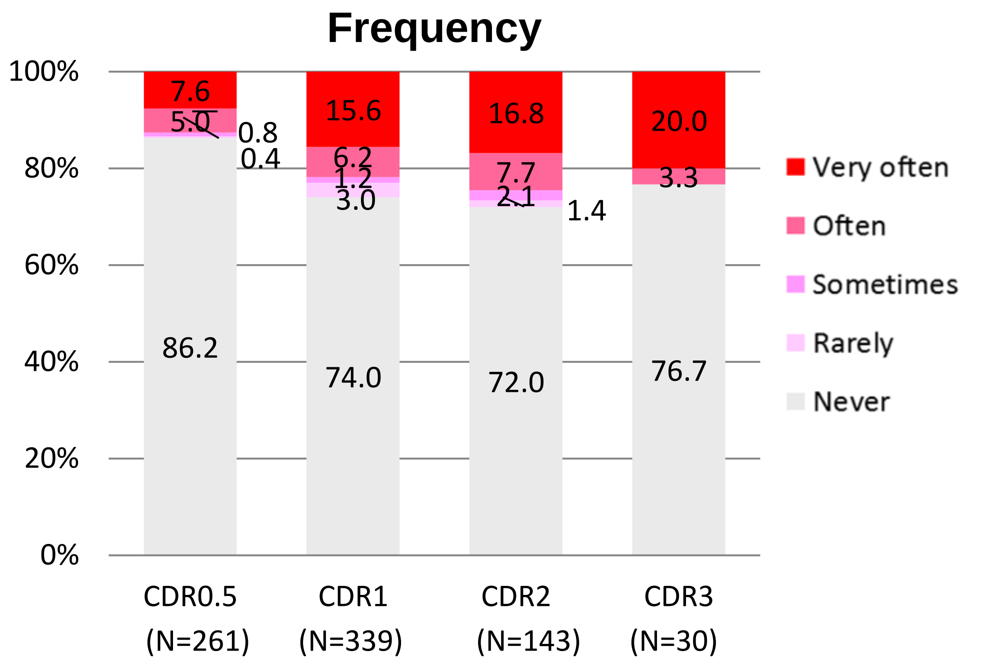

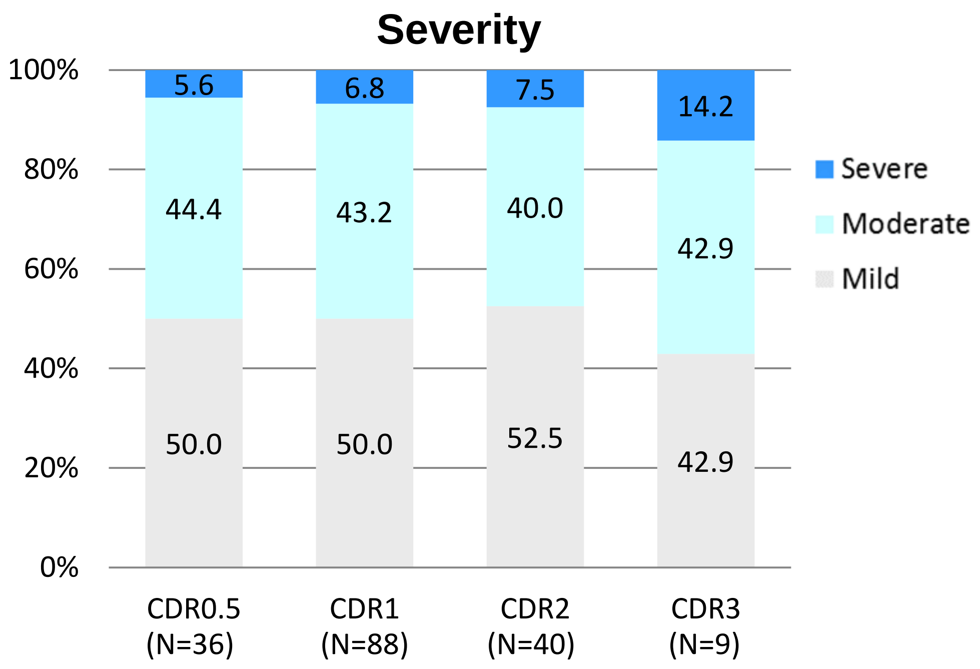

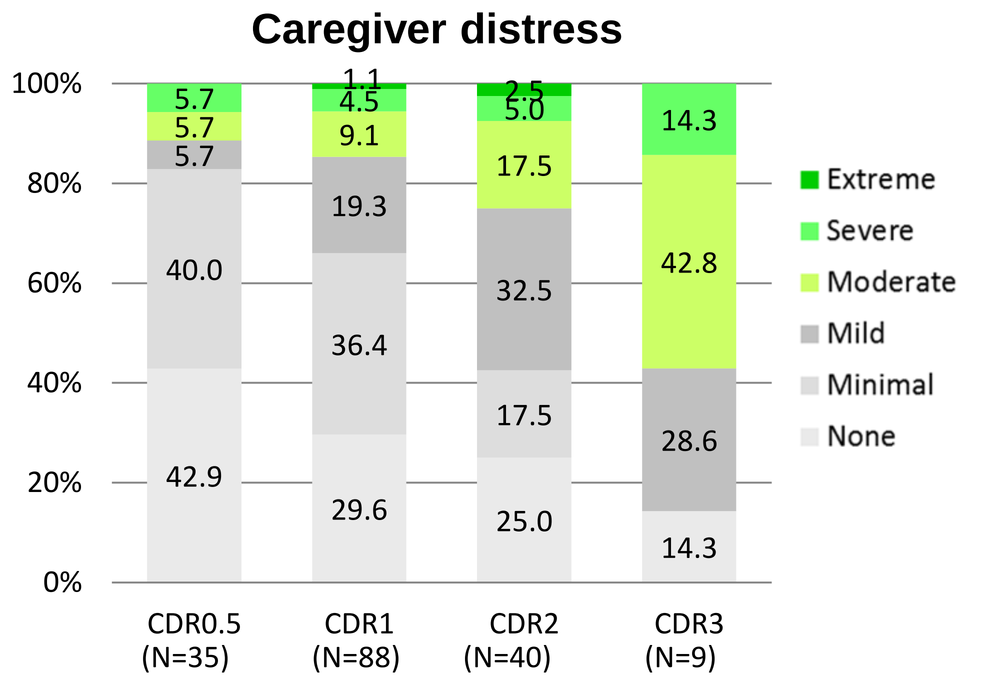

Supplement: S1 File — (PDF) [file pone.0161092.s001.pdf]
