# Supplementary material for: Differences of Behavioral and Psychological Symptoms of Dementia in Disease Severity in Four Major Dementias
Source: PLoS One. 2016 Aug 18;11(8):e0161092. doi: 10.1371/journal.pone.0161092 (PMC4990196; doi:10.1371/journal.pone.0161092)

S2 File.

Charts for BPSD in Dementia with Lewy bodies
by disease severity

Delusions

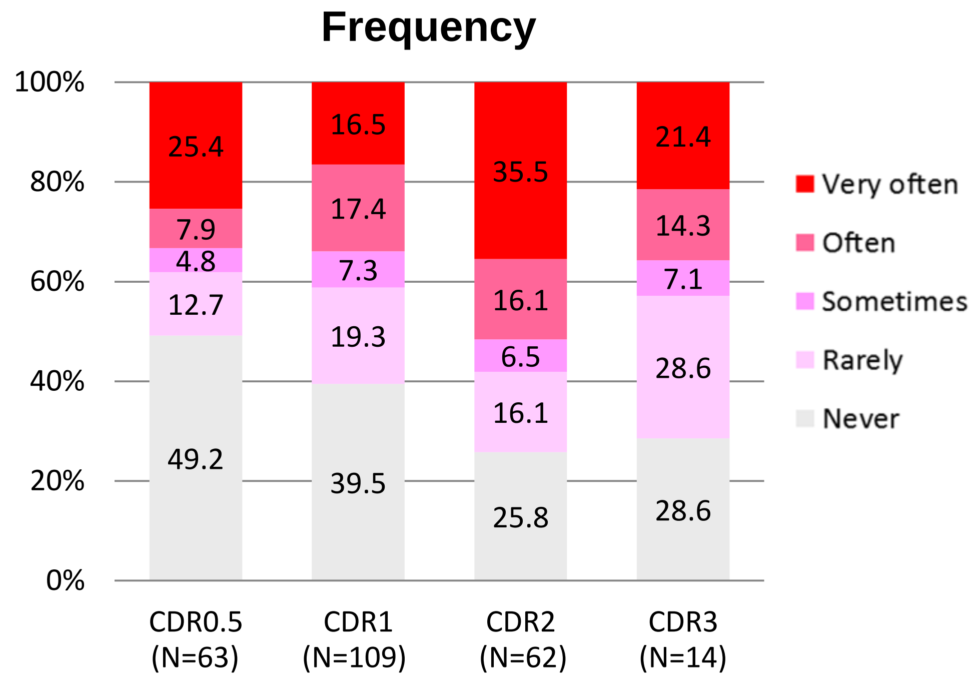
Frequency
100%
80%
60%
40%
20%
0%
25.4
7.9
4.8
12.7
49.2
16.5
17.4
7.3
19.3
39.5
35.5
16.1
6.5
16.1
25.8
21.4
14.3
7.1
28.6
28.6
CDR0.5 (N=63)
CDR1 (N=109)
CDR2 (N=62)
CDR3 (N=14)
Very often
Often
Sometimes
Rarely
Never

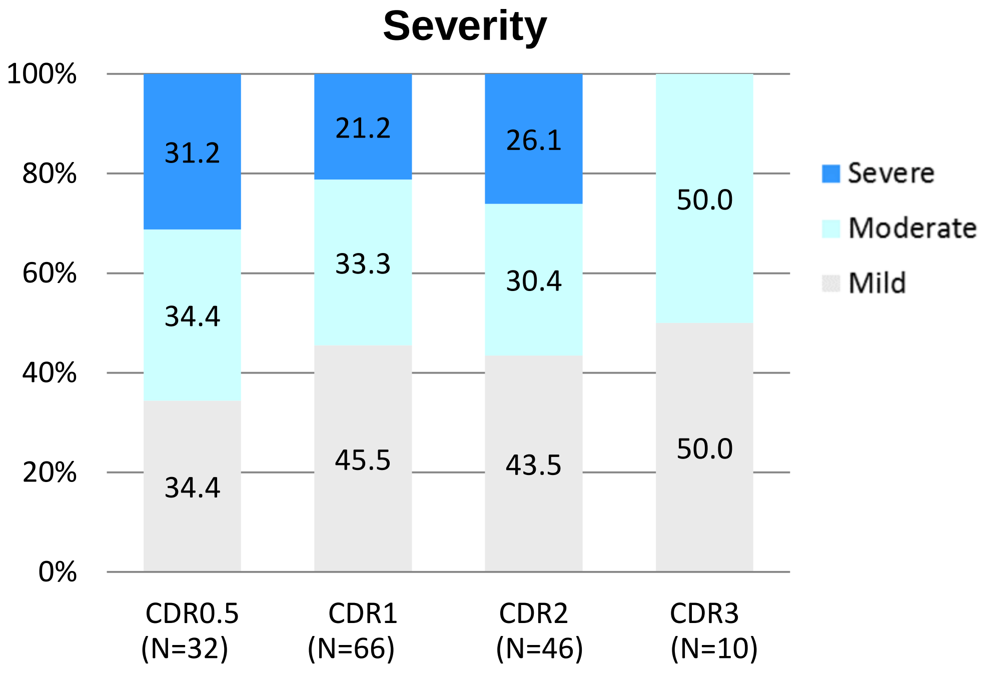
Severity
100%
80%
60%
40%
20%
0%
31.2
34.4
34.4
21.2
33.3
45.5
26.1
30.4
43.5
50.0
50.0
CDR0.5 (N=32)
CDR1 (N=66)
CDR2 (N=46)
CDR3 (N=10)
Severe
Moderate
Mild

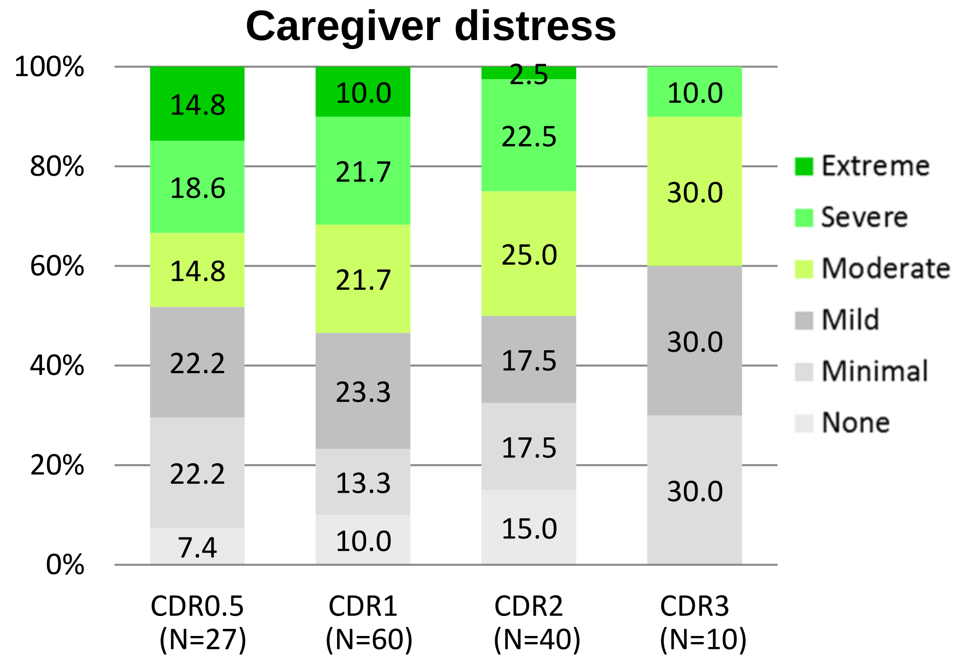
Caregiver distress
100%
80%
60%
40%
20%
0%
14.8
18.6
14.8
22.2
22.2
7.4
10.0
21.7
21.7
23.3
13.3
10.0
2.5
22.5
25.0
17.5
17.5
15.0
10.0
30.0
30.0
30.0
CDR0.5 (N=27)
CDR1 (N=60)
CDR2 (N=40)
CDR3 (N=10)
Extreme
Severe
Moderate
Mild
Minimal
None

Hallucinations

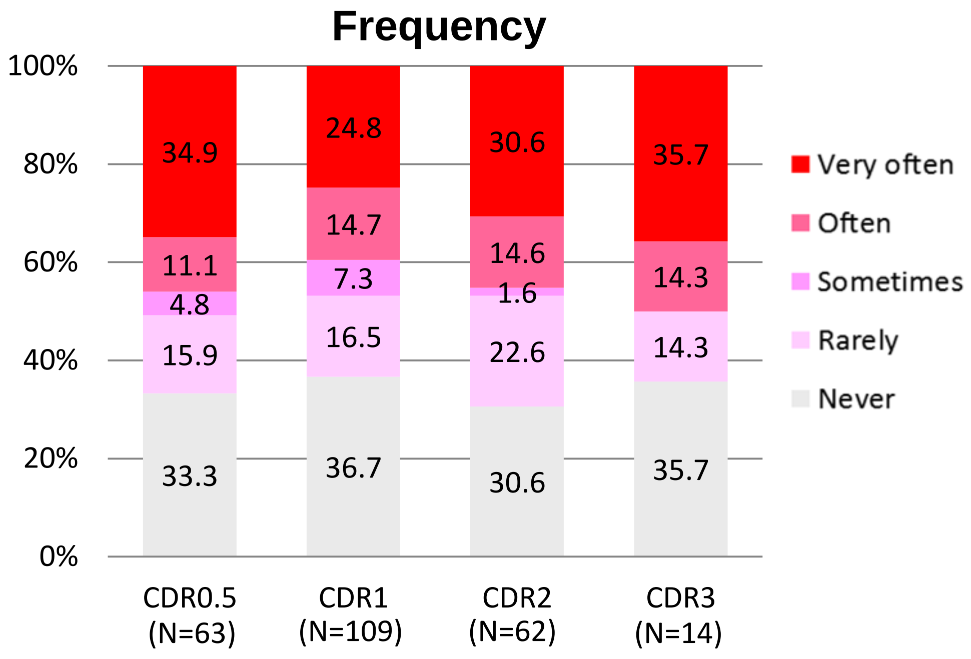

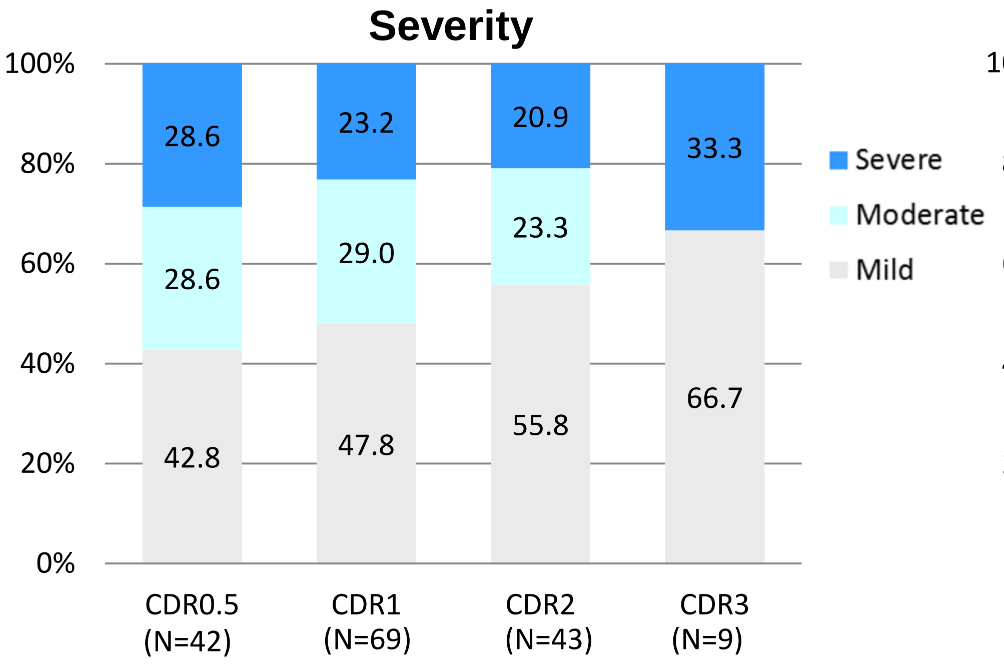

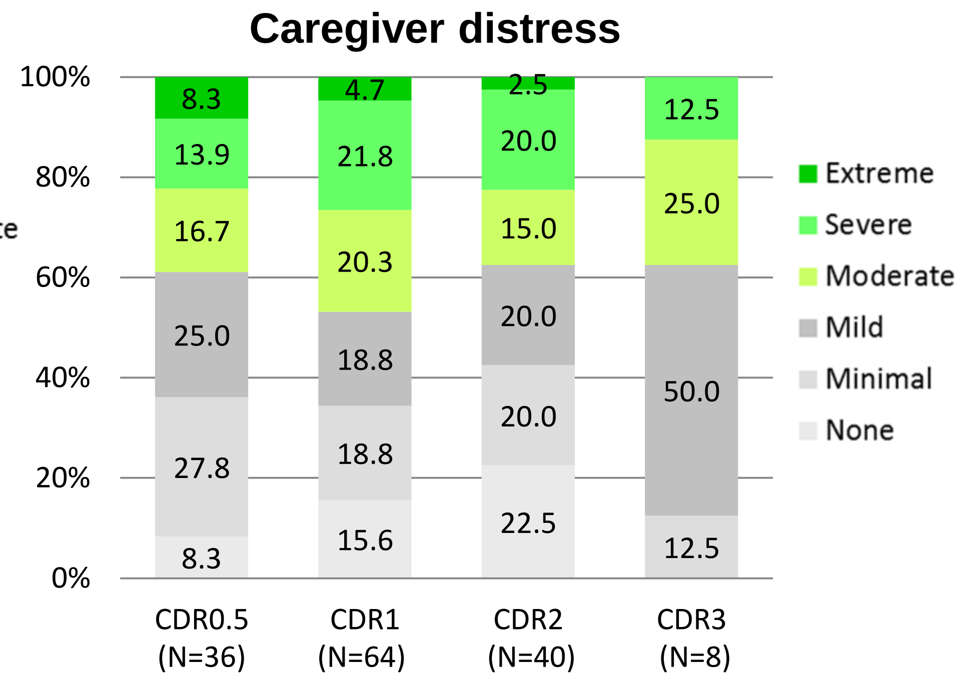

Agitation

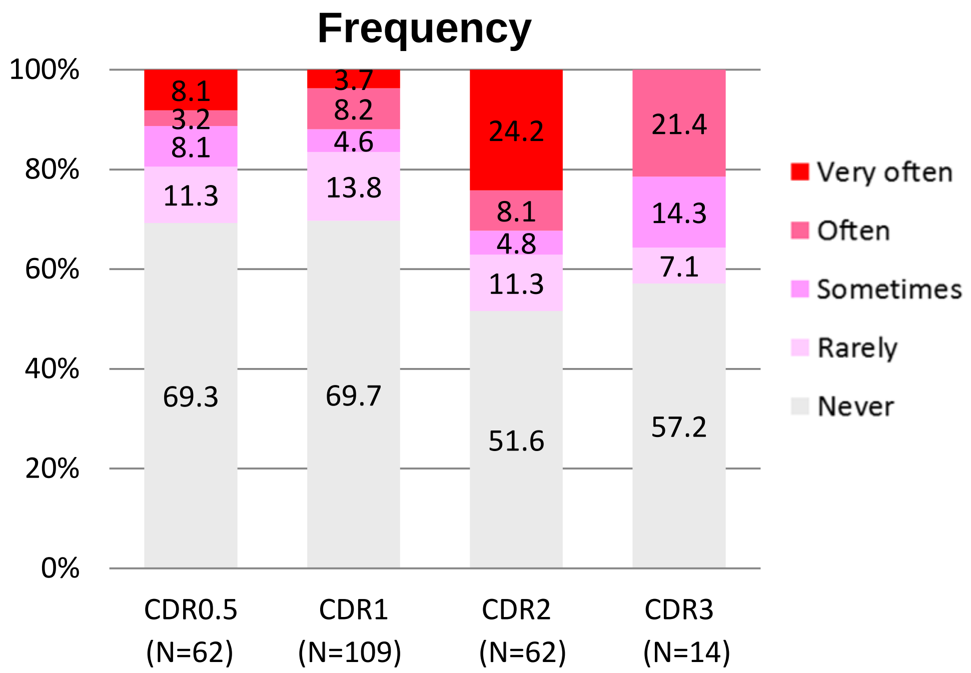
Frequency
100%
80%
60%
40%
20%
0%
8.1
3.2
8.1
11.3
69.3
3.7
8.2
4.6
13.8
69.7
24.2
8.1
4.8
11.3
51.6
21.4
14.3
7.1
57.2
Very often
Often
Sometimes
Rarely
Never
CDR0.5 (N=62)
CDR1 (N=109)
CDR2 (N=62)
CDR3 (N=14)

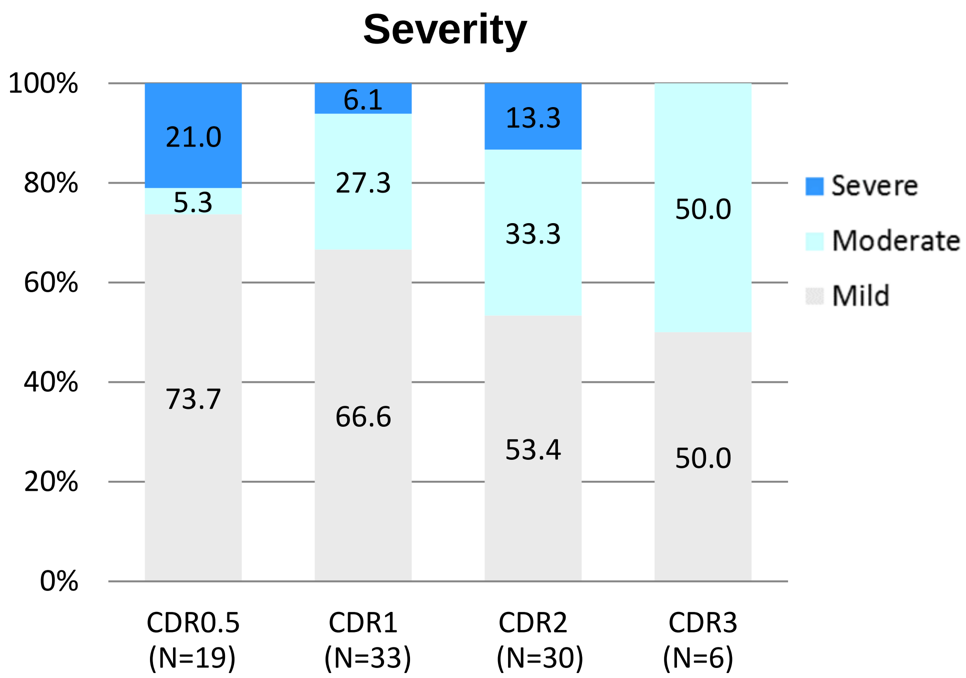
Severity
100%
80%
60%
40%
20%
0%
21.0
5.3
73.7
6.1
27.3
66.6
13.3
33.3
53.4
50.0
50.0
Severe
Moderate
Mild
CDR0.5 (N=19)
CDR1 (N=33)
CDR2 (N=30)
CDR3 (N=6)

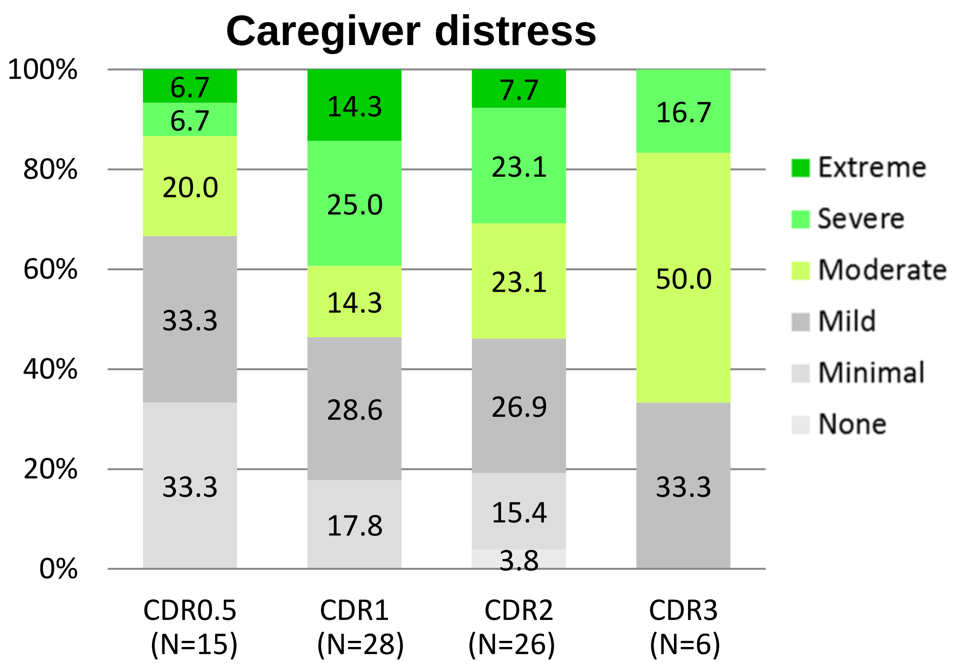
Caregiver distress
100%
80%
60%
40%
20%
0%
6.7
6.7
20.0
33.3
33.3
14.3
25.0
14.3
28.6
17.8
7.7
23.1
23.1
26.9
15.4
3.8
16.7
50.0
33.3
Extreme
Severe
Moderate
Mild
Minimal
None
CDR0.5 (N=15)
CDR1 (N=28)
CDR2 (N=26)
CDR3 (N=6)

Depression

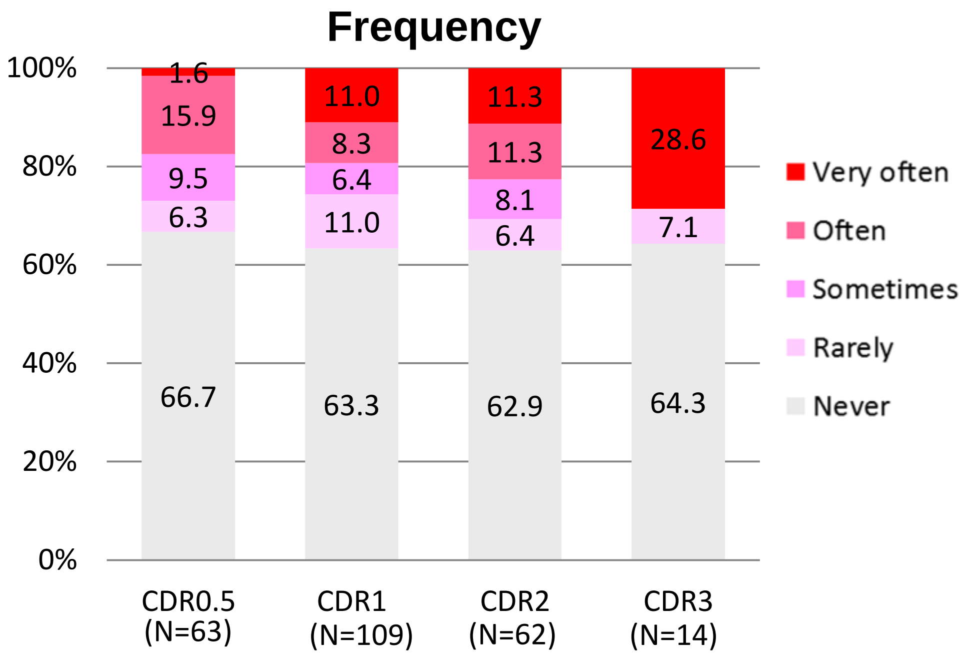
Frequency
100%
80%
60%
40%
20%
0%
1.6
15.9
9.5
6.3
66.7
11.0
8.3
6.4
11.0
63.3
11.3
11.3
8.1
6.4
62.9
28.6
7.1
64.3
CDR0.5 (N=63)
CDR1 (N=109)
CDR2 (N=62)
CDR3 (N=14)
Very often
Often
Sometimes
Rarely
Never

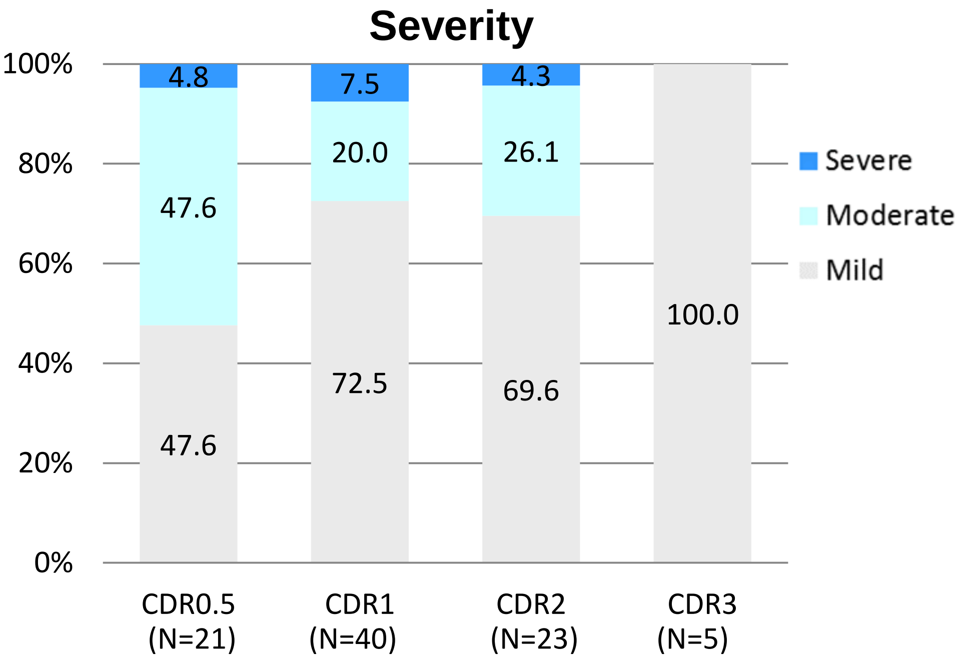
Severity
100%
80%
60%
40%
20%
0%
4.8
47.6
47.6
7.5
20.0
72.5
4.3
26.1
69.6
100.0
CDR0.5 (N=21)
CDR1 (N=40)
CDR2 (N=23)
CDR3 (N=5)
Severe
Moderate
Mild

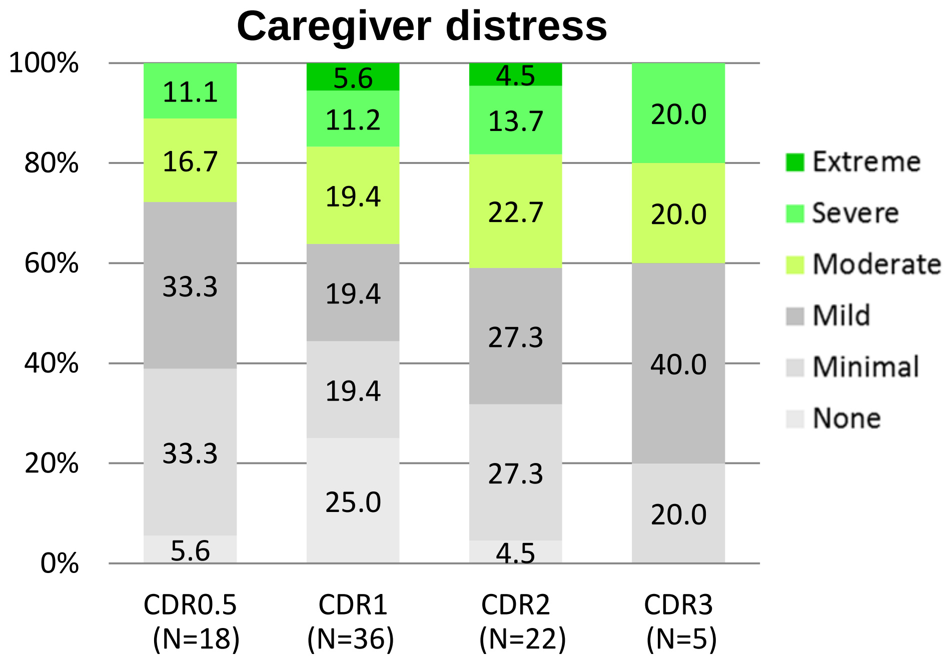
Caregiver distress
100%
80%
60%
40%
20%
0%
11.1
16.7
33.3
33.3
5.6
5.6
11.2
19.4
19.4
19.4
25.0
4.5
13.7
22.7
27.3
27.3
4.5
20.0
20.0
40.0
20.0
CDR0.5 (N=18)
CDR1 (N=36)
CDR2 (N=22)
CDR3 (N=5)
Extreme
Severe
Moderate
Mild
Minimal
None

Anxiety

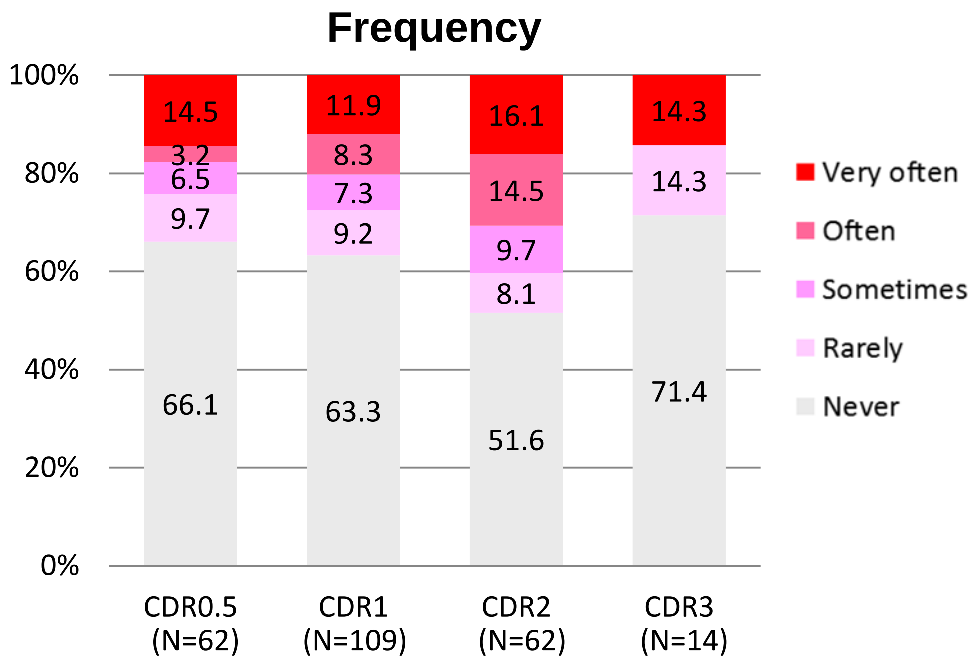
Frequency
100%
80%
60%
40%
20%
0%
14.5
3.2
6.5
9.7
66.1
11.9
8.3
7.3
9.2
63.3
16.1
14.5
9.7
8.1
51.6
14.3
14.3
71.4
CDR0.5 (N=62)
CDR1 (N=109)
CDR2 (N=62)
CDR3 (N=14)
Very often
Often
Sometimes
Rarely
Never

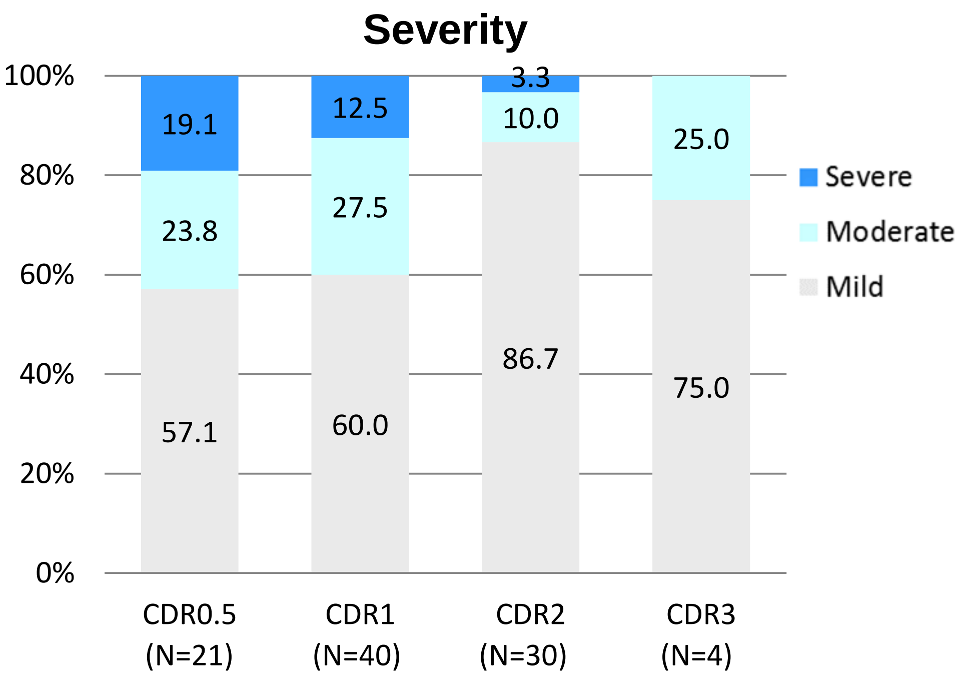
Severity
100%
80%
60%
40%
20%
0%
19.1
23.8
57.1
12.5
27.5
60.0
3.3
10.0
86.7
25.0
75.0
CDR0.5 (N=21)
CDR1 (N=40)
CDR2 (N=30)
CDR3 (N=4)
Severe
Moderate
Mild

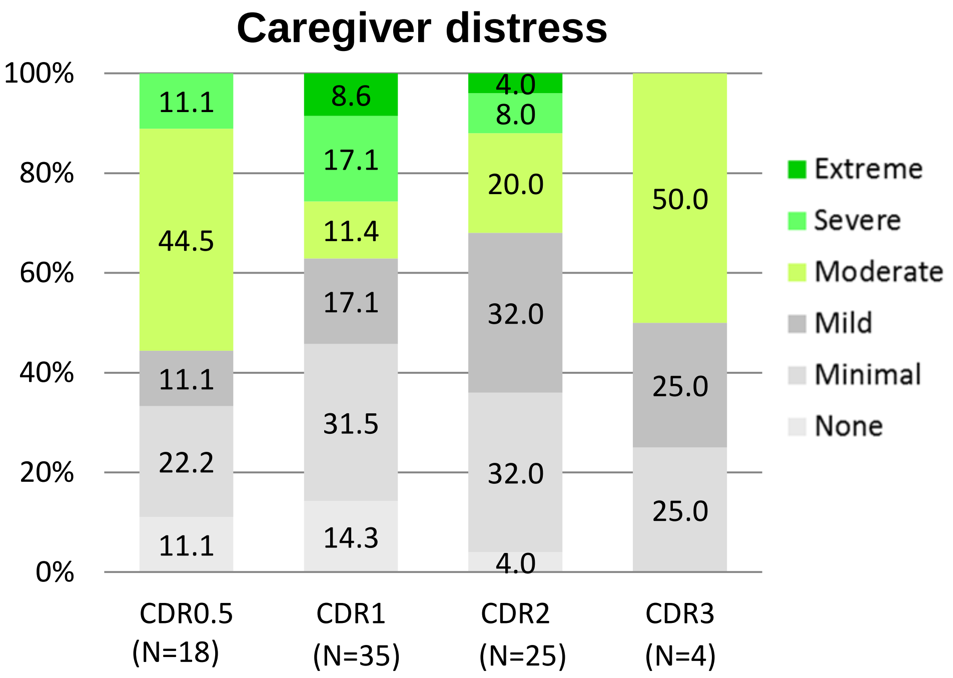
Caregiver distress
100%
80%
60%
40%
20%
0%
11.1
44.5
11.1
22.2
11.1
8.6
17.1
11.4
17.1
31.5
14.3
4.0
8.0
20.0
32.0
32.0
4.0
50.0
25.0
25.0
CDR0.5 (N=18)
CDR1 (N=35)
CDR2 (N=25)
CDR3 (N=4)
Extreme
Severe
Moderate
Mild
Minimal
None

Euphoria

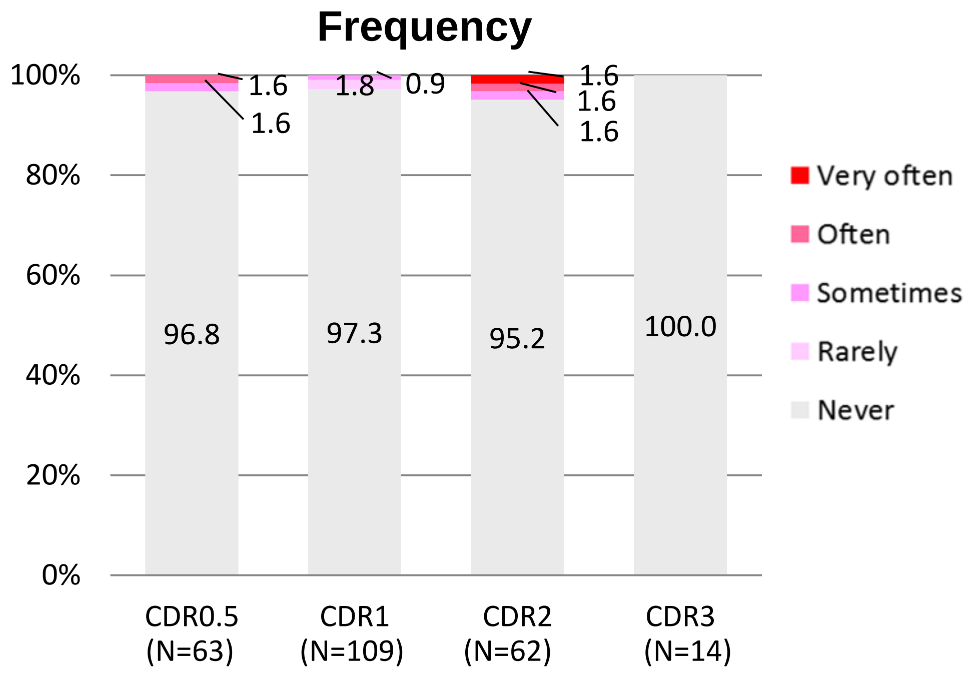

Frequency
100%
80%
60%
40%
20%
0%
1.6
1.6
96.8
1.8
0.9
97.3
1.6
1.6
1.6
95.2
100.0
CDR0.5 (N=63)
CDR1 (N=109)
CDR2 (N=62)
CDR3 (N=14)
Very often
Often
Sometimes
Rarely
Never

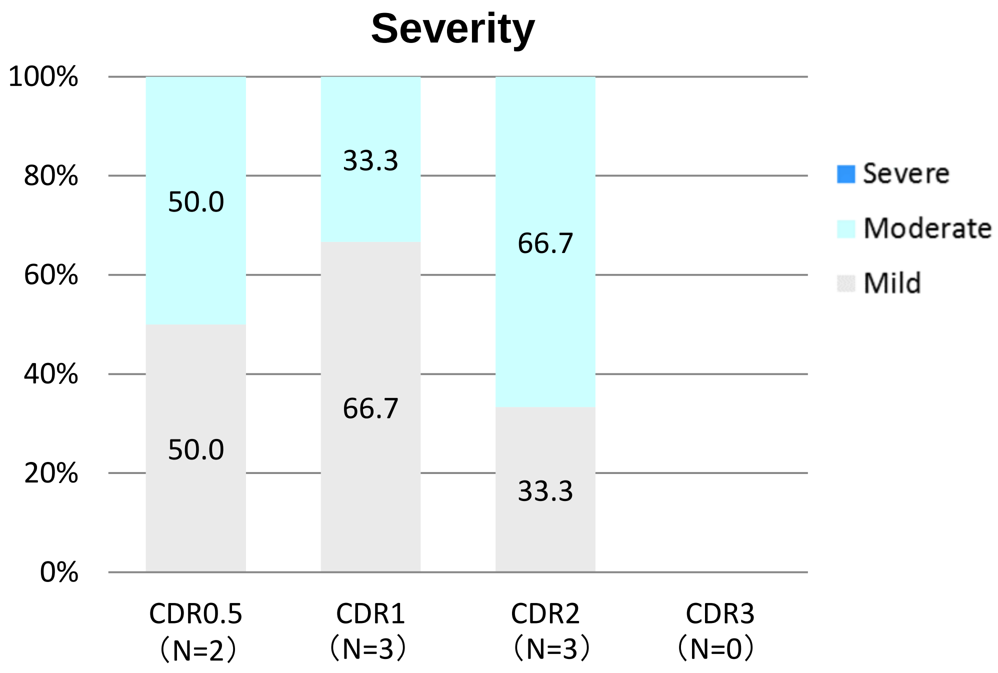

Severity
100%
80%
60%
40%
20%
0%
50.0
50.0
33.3
66.7
66.7
33.3
CDR0.5 (N=2)
CDR1 (N=3)
CDR2 (N=3)
CDR3 (N=0)
Severe
Moderate
Mild

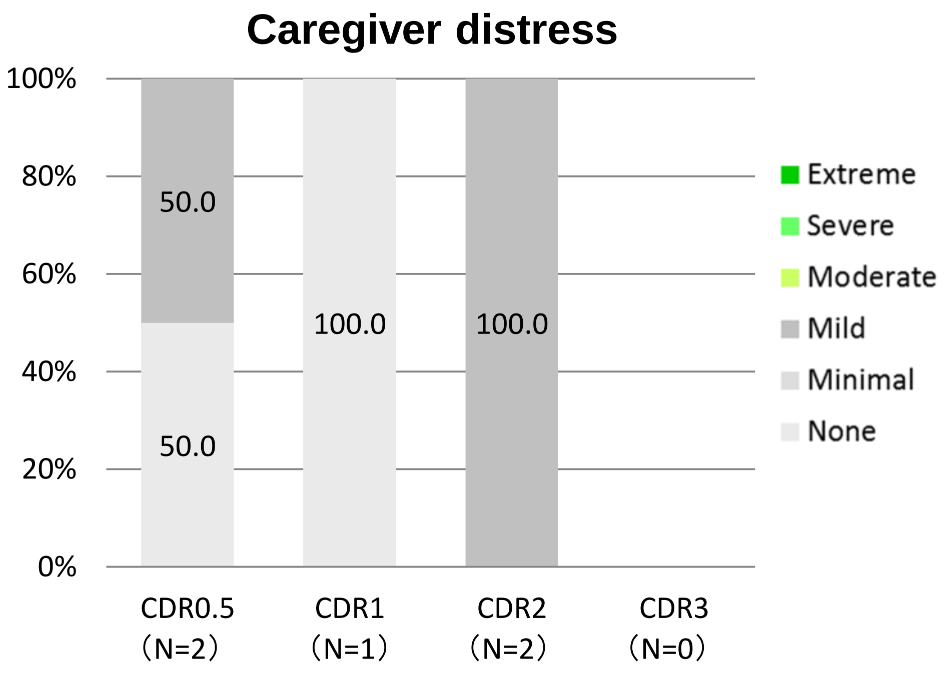

Caregiver distress
100%
80%
60%
40%
20%
0%
50.0
50.0
100.0
100.0
CDR0.5 (N=2)
CDR1 (N=1)
CDR2 (N=2)
CDR3 (N=0)
Extreme
Severe
Moderate
Mild
Minimal
None

Apathy

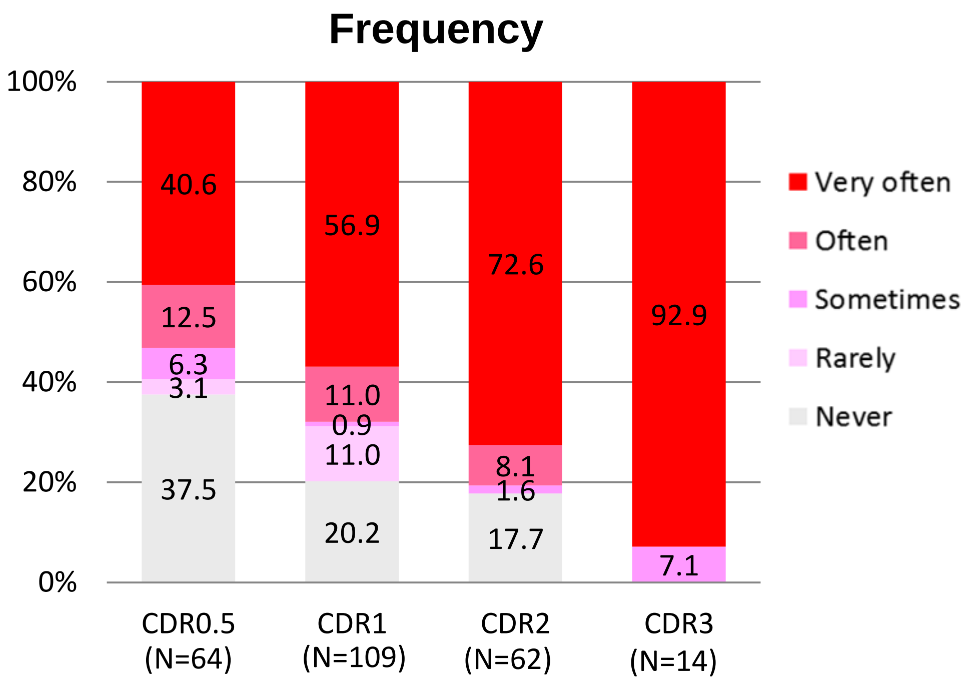
Frequency
100%
80%
60%
40%
20%
0%
40.6
12.5
6.3
3.1
37.5
56.9
11.0
0.9
11.0
20.2
72.6
8.1
1.6
17.7
92.9
7.1
CDR0.5 (N=64)
CDR1 (N=109)
CDR2 (N=62)
CDR3 (N=14)
Very often
Often
Sometimes
Rarely
Never

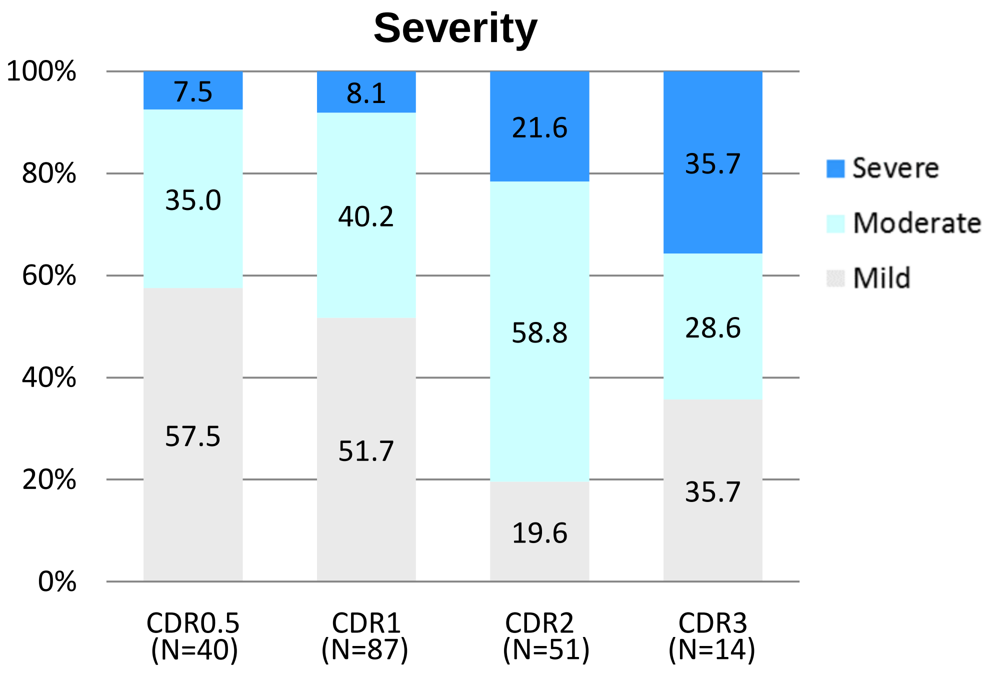
Severity
100%
80%
60%
40%
20%
0%
7.5
35.0
57.5
8.1
40.2
51.7
21.6
58.8
19.6
35.7
28.6
35.7
CDR0.5 (N=40)
CDR1 (N=87)
CDR2 (N=51)
CDR3 (N=14)
Severe
Moderate
Mild

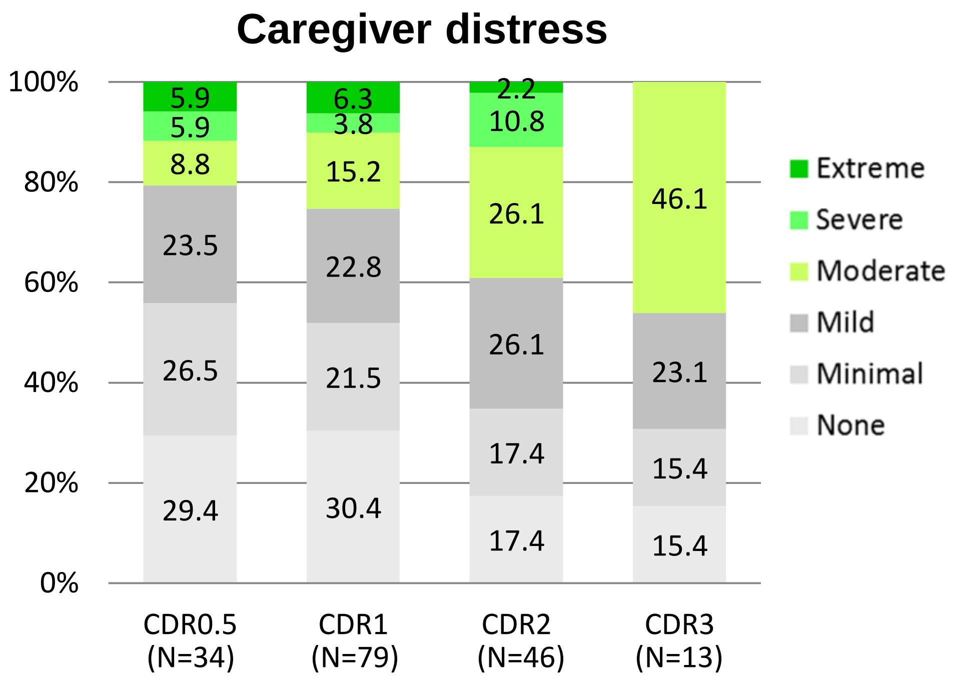
Caregiver distress
100%
80%
60%
40%
20%
0%
5.9
5.9
8.8
23.5
26.5
29.4
6.3
3.8
15.2
22.8
21.5
30.4
2.2
10.8
26.1
26.1
17.4
17.4
46.1
23.1
15.4
15.4
CDR0.5 (N=34)
CDR1 (N=79)
CDR2 (N=46)
CDR3 (N=13)
Extreme
Severe
Moderate
Mild
Minimal
None

Disinhibition

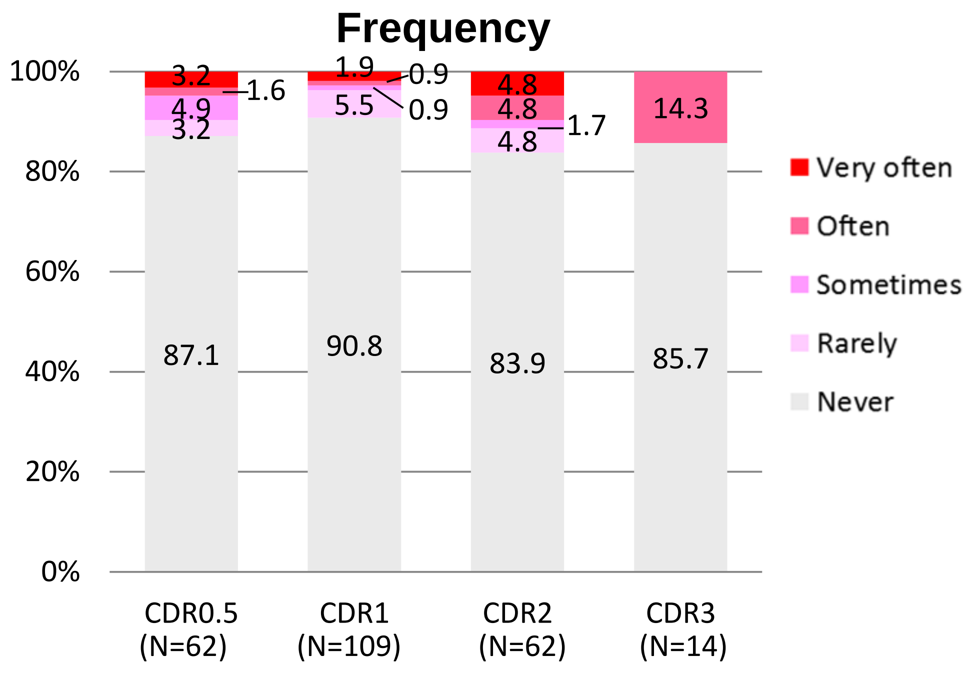

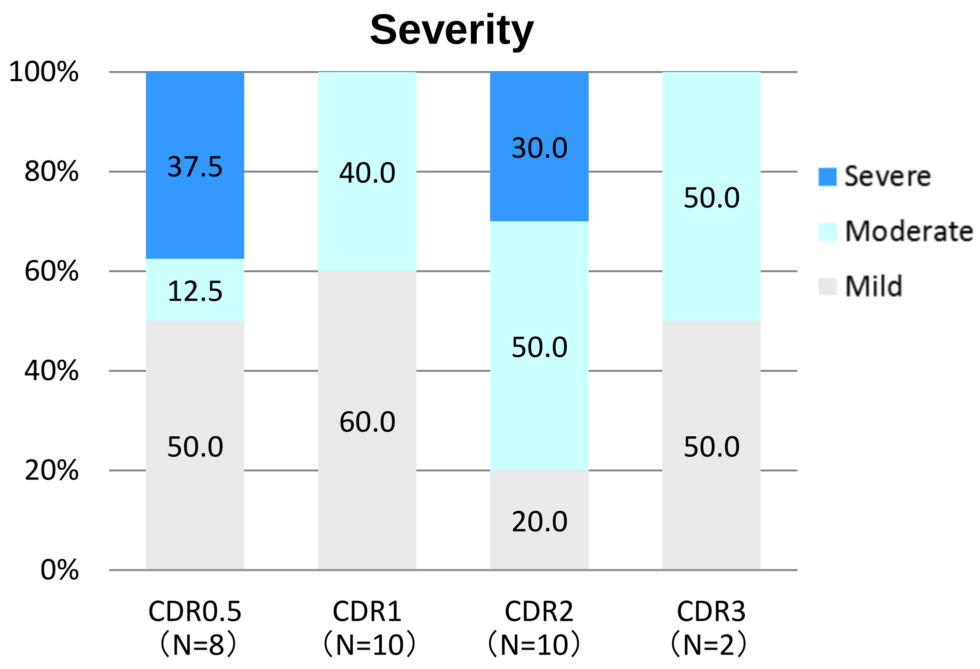

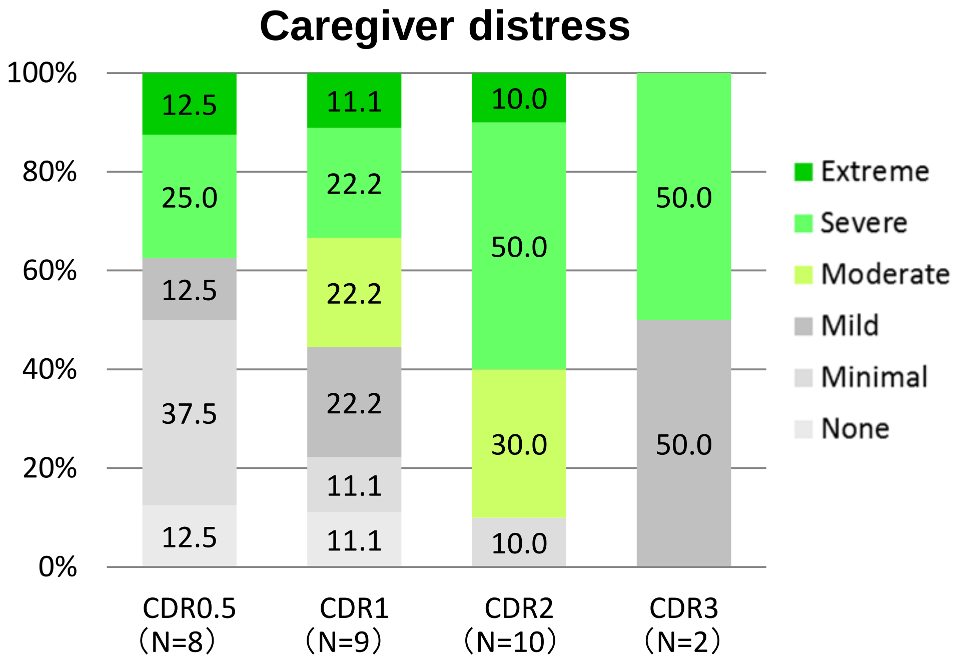

Irritability

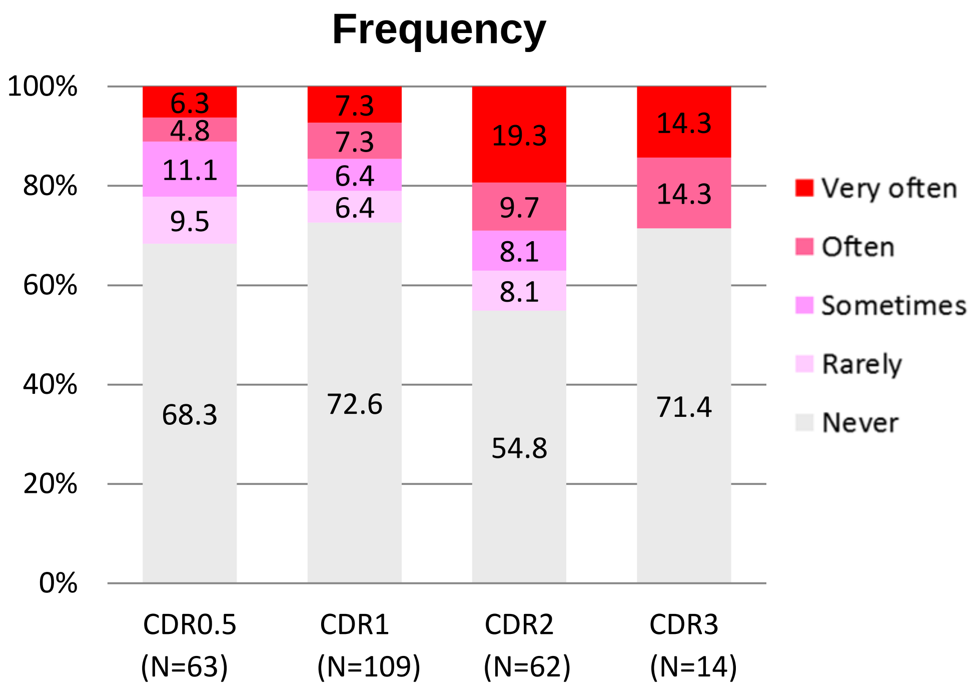

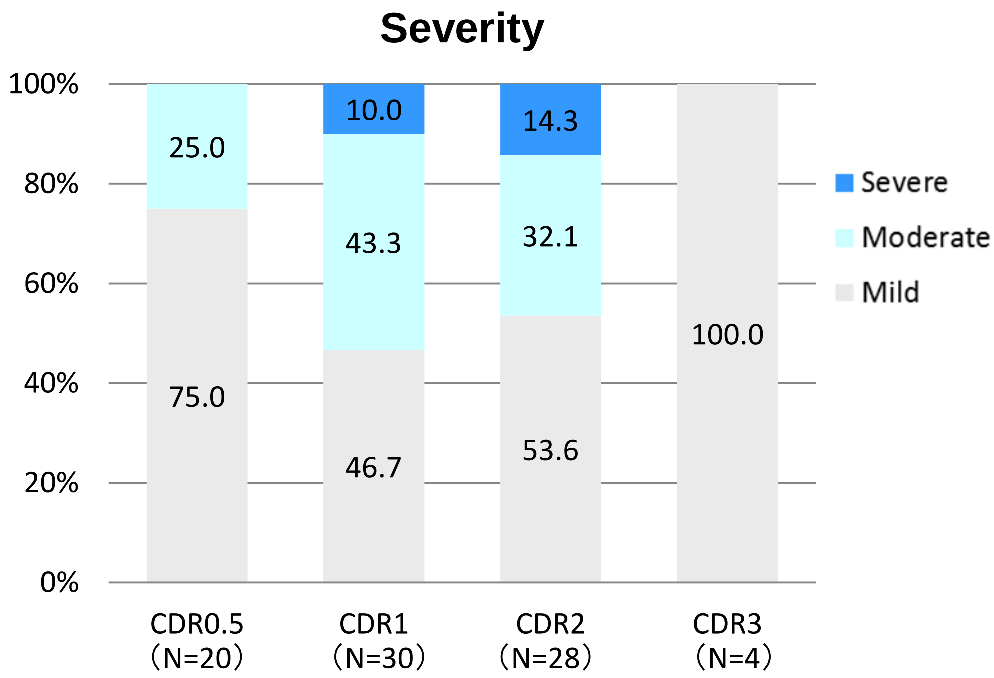

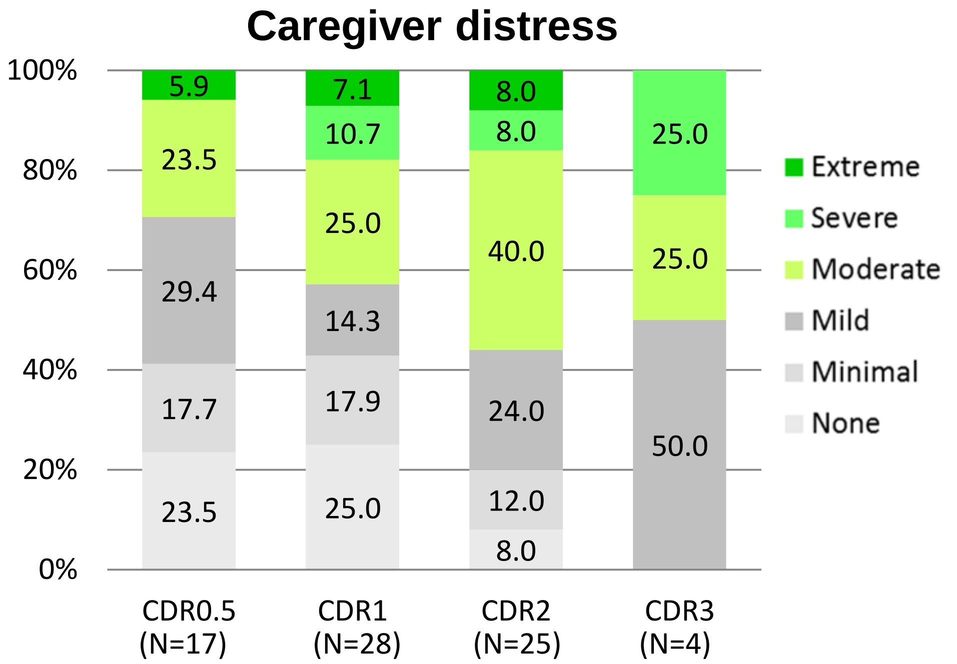

Aberrant motor behavior

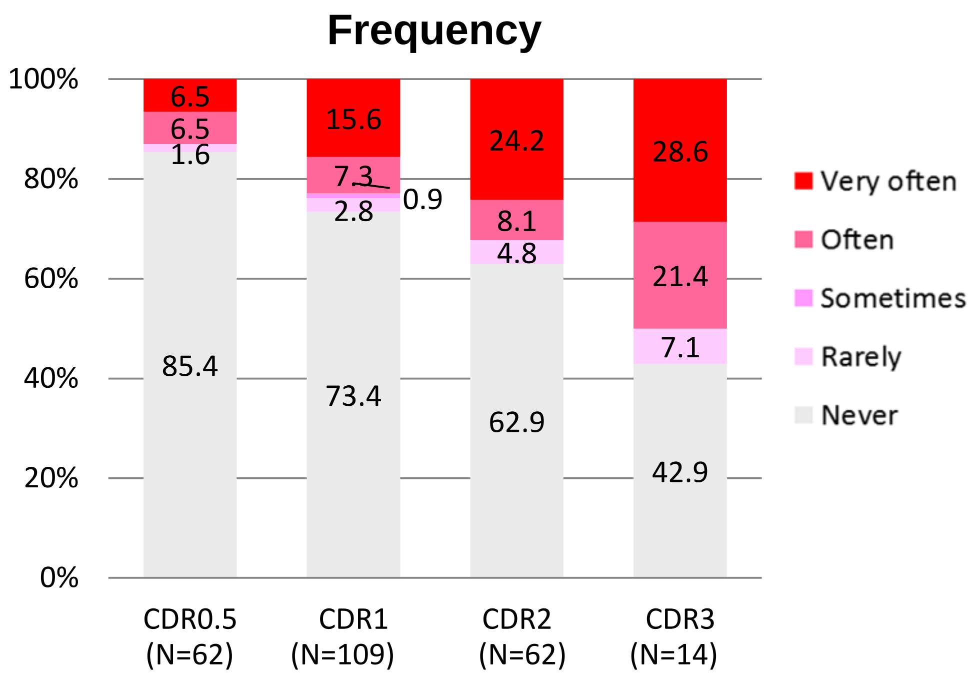

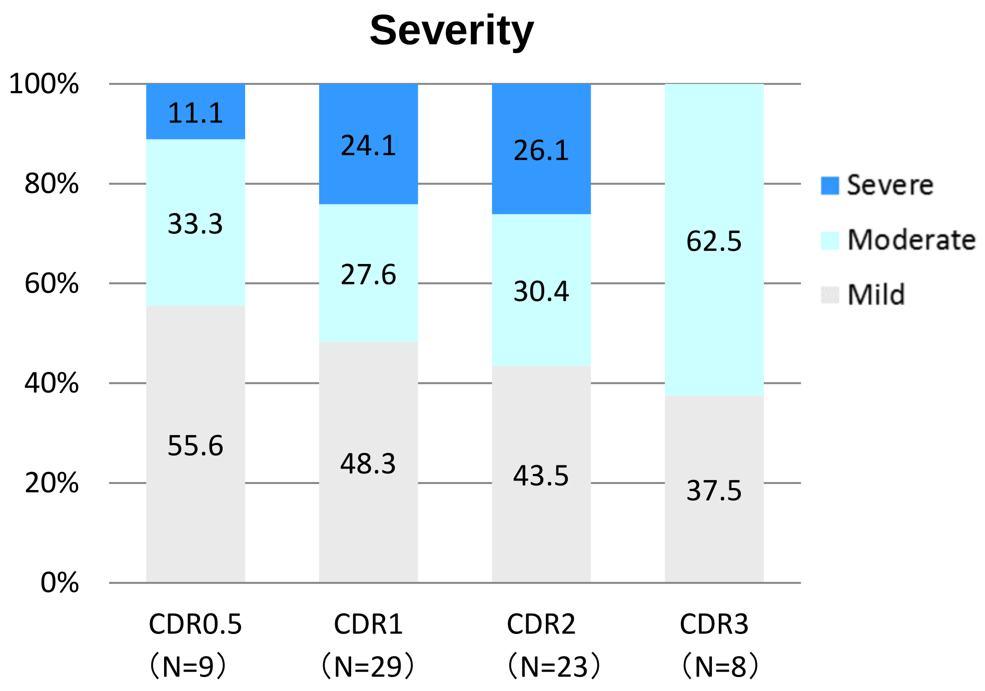

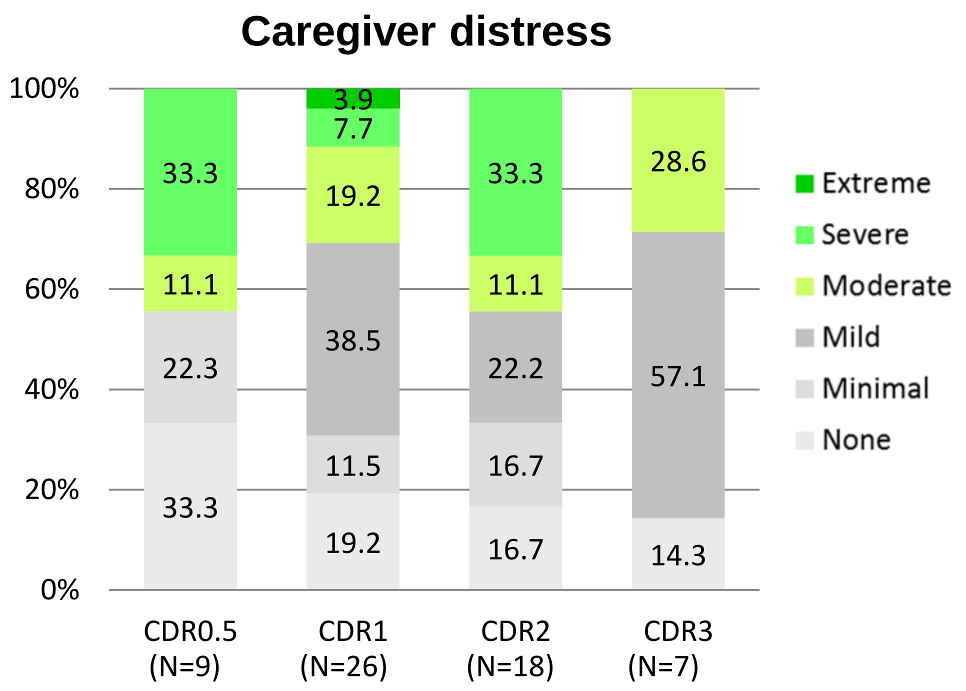

Sleep disturbances

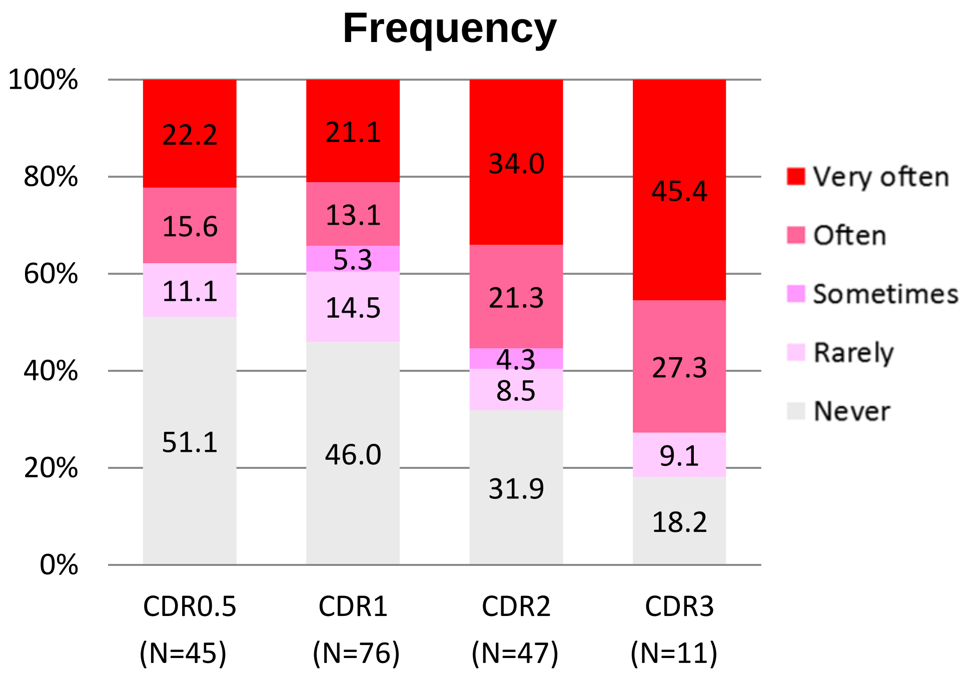

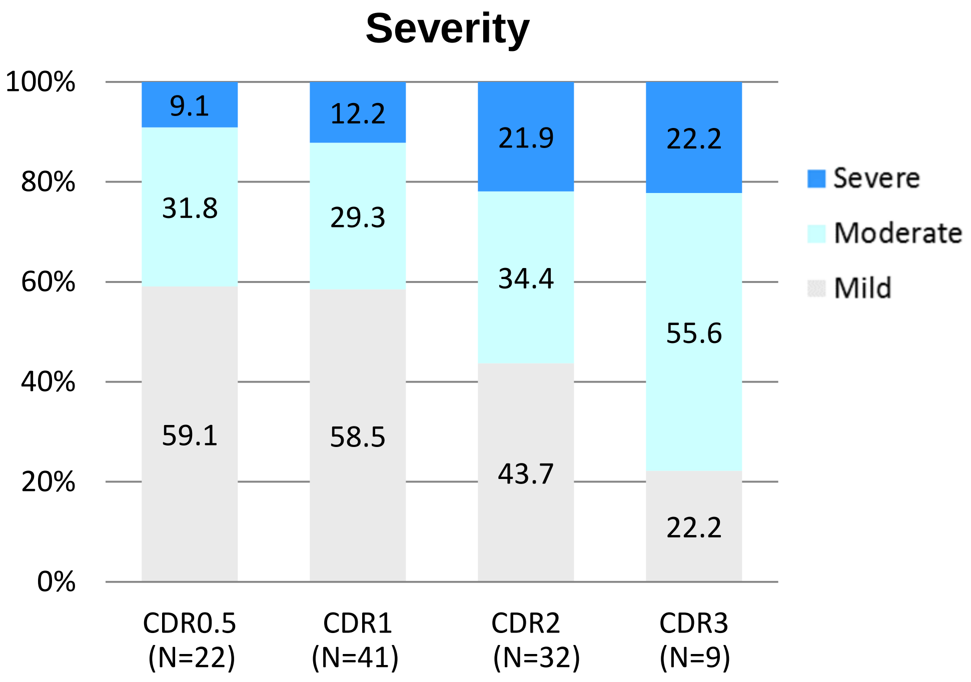

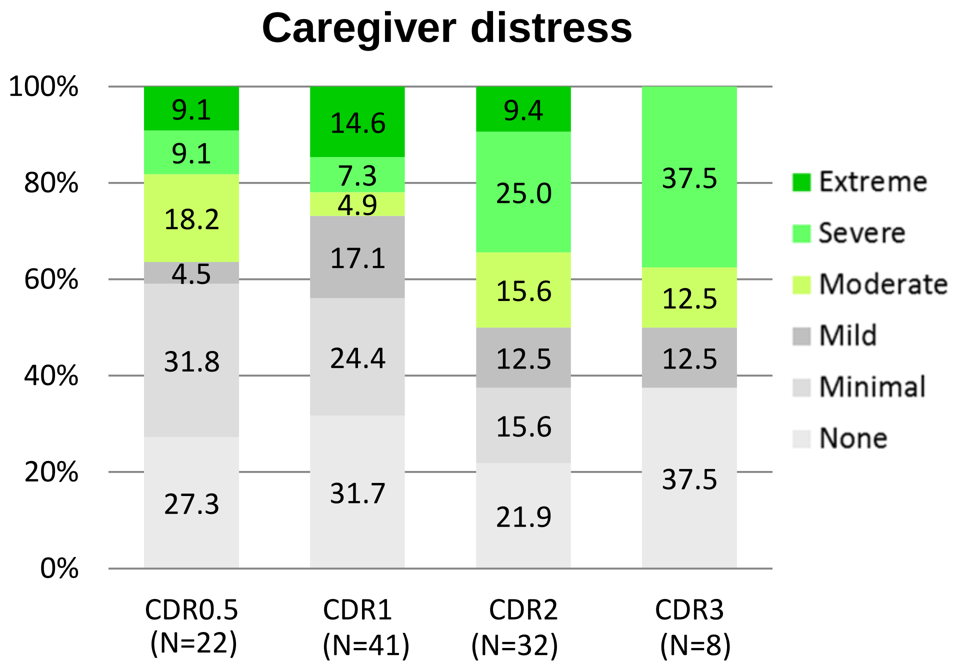

Eating abnormalities

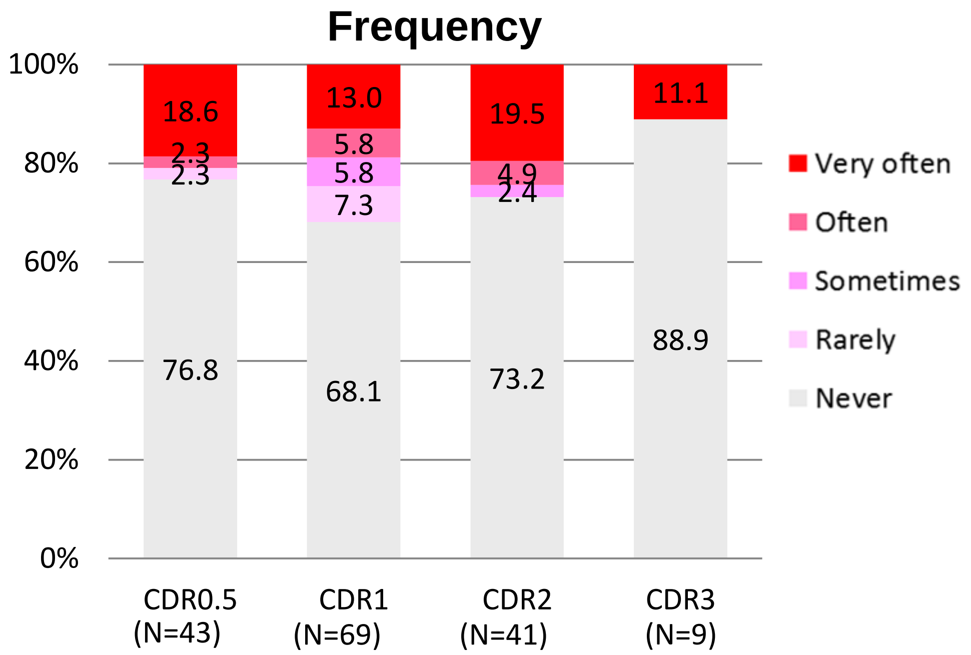

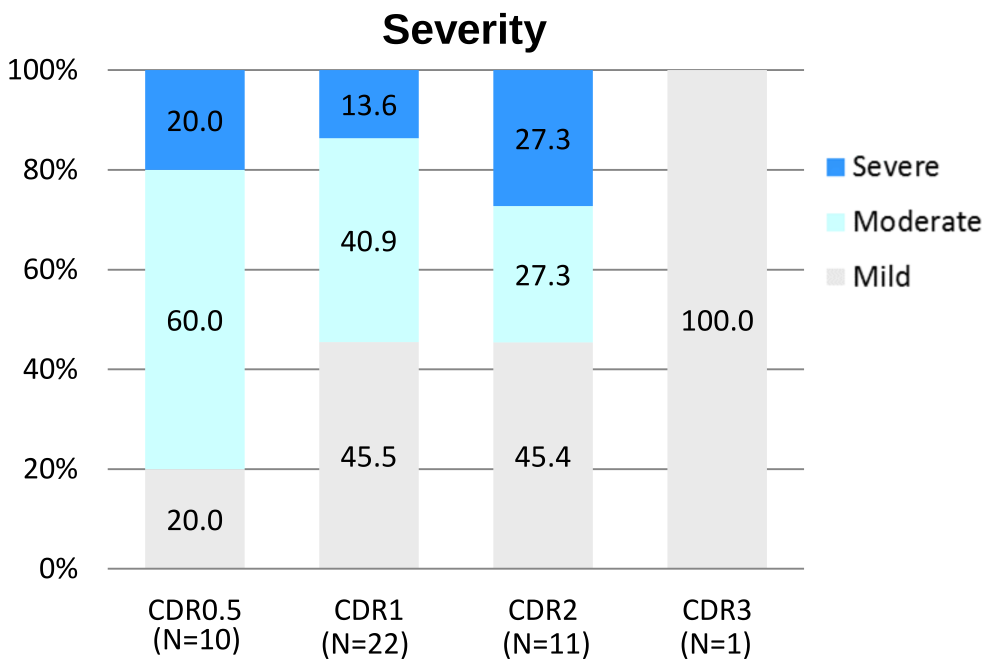

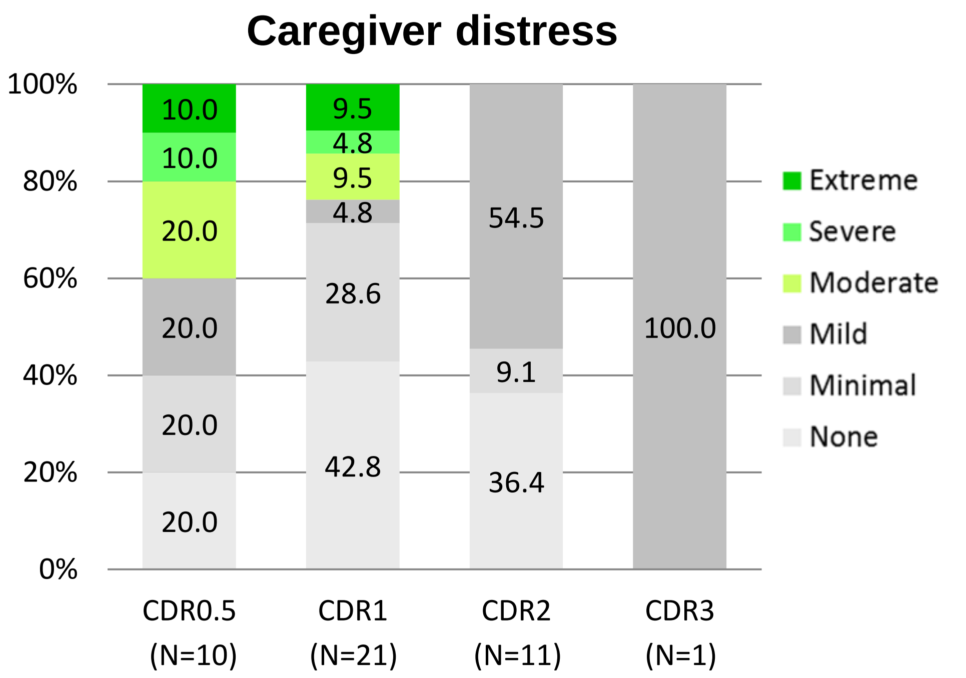

Supplement: S2 File — (PDF) [file pone.0161092.s002.pdf]
