# Supplementary material for: Differences of Behavioral and Psychological Symptoms of Dementia in Disease Severity in Four Major Dementias
Source: PLoS One. 2016 Aug 18;11(8):e0161092. doi: 10.1371/journal.pone.0161092 (PMC4990196; doi:10.1371/journal.pone.0161092)

S3 File.

Charts for BPSD in Vascular dementia
by disease severity

Delusions

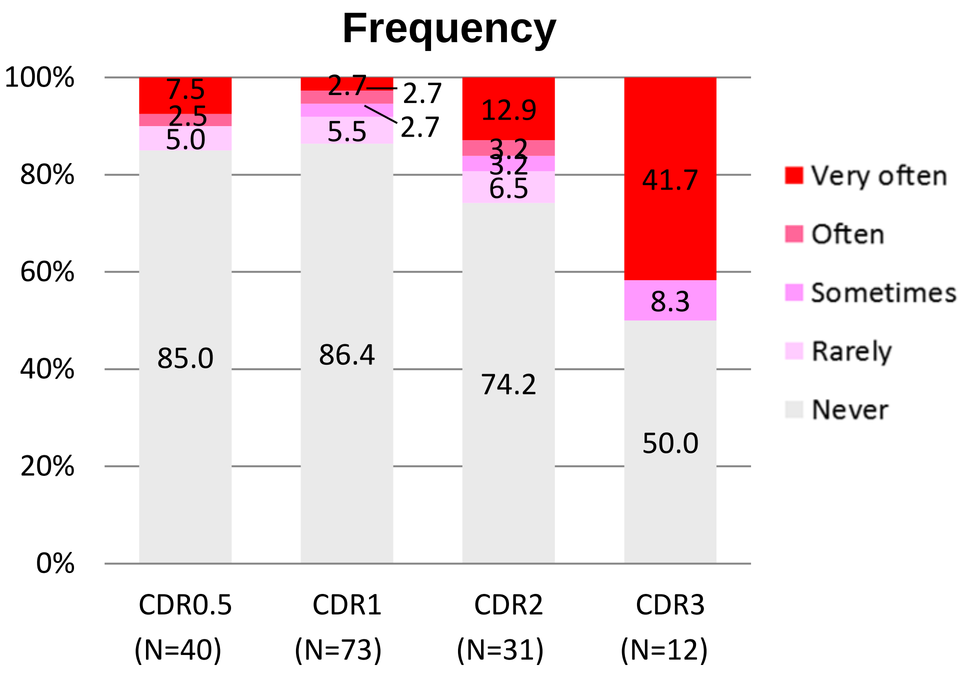

Frequency
100%
80%
60%
40%
20%
0%
7.5
2.5
5.0
85.0
2.7
2.7
2.7
5.5
86.4
12.9
3.2
3.2
6.5
74.2
41.7
8.3
50.0
CDR0.5 (N=40)
CDR1 (N=73)
CDR2 (N=31)
CDR3 (N=12)
Very often
Often
Sometimes
Rarely
Never

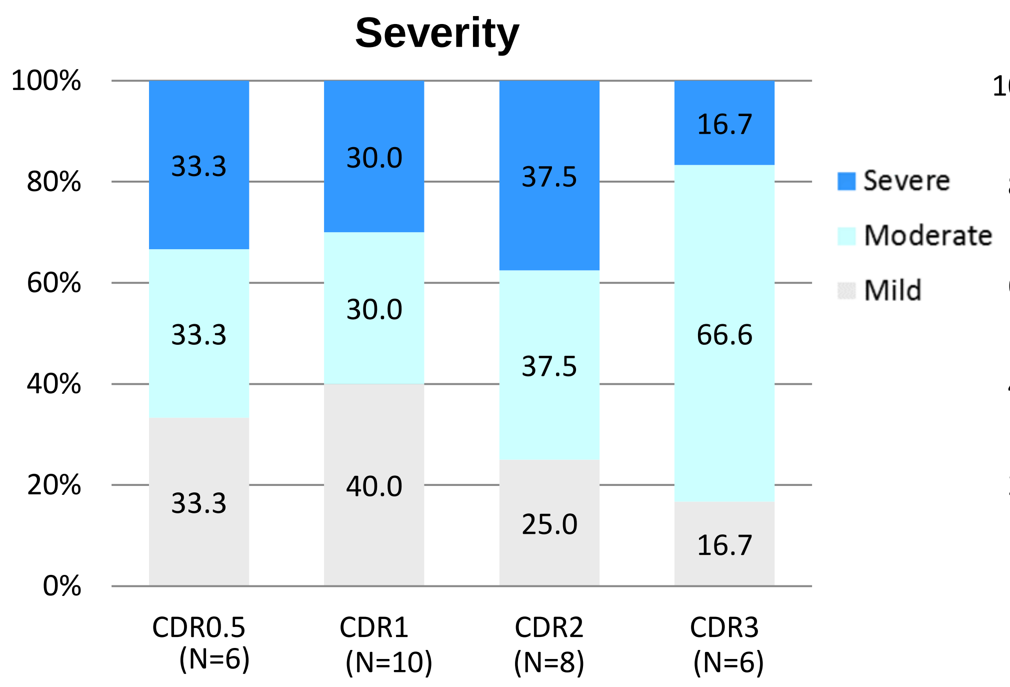

Severity
100%
80%
60%
40%
20%
0%
33.3
33.3
33.3
30.0
30.0
40.0
37.5
37.5
25.0
16.7
66.6
16.7
CDR0.5 (N=6)
CDR1 (N=10)
CDR2 (N=8)
CDR3 (N=6)
Severe
Moderate
Mild

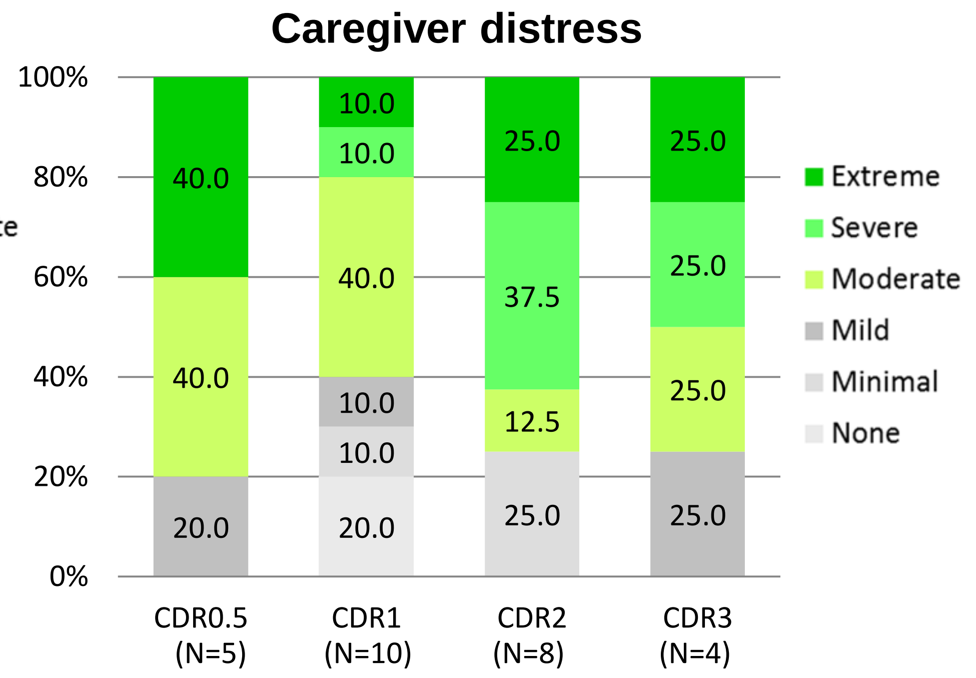

Caregiver distress
100%
80%
60%
40%
20%
0%
40.0
40.0
20.0
10.0
10.0
40.0
10.0
10.0
20.0
25.0
37.5
12.5
25.0
25.0
25.0
25.0
25.0
CDR0.5 (N=5)
CDR1 (N=10)
CDR2 (N=8)
CDR3 (N=4)
Extreme
Severe
Moderate
Mild
Minimal
None

Hallucinations

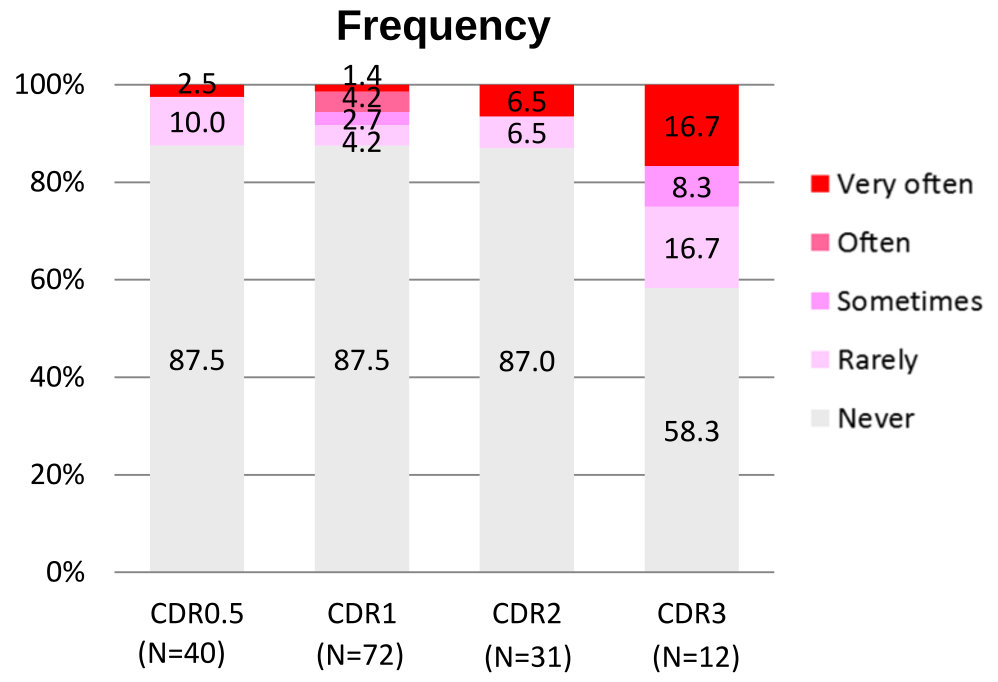

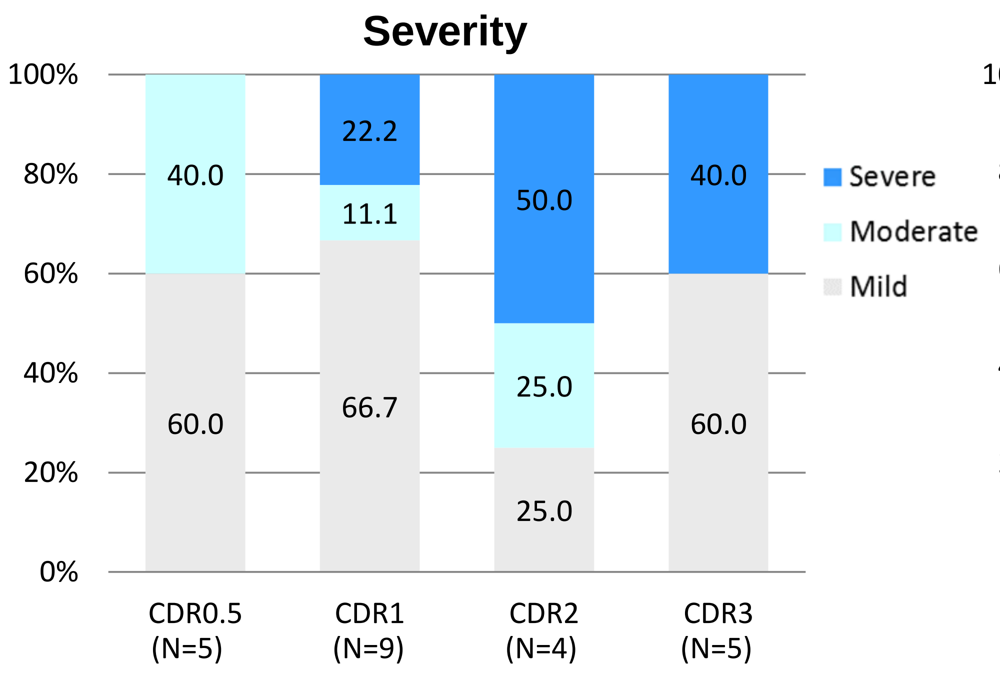

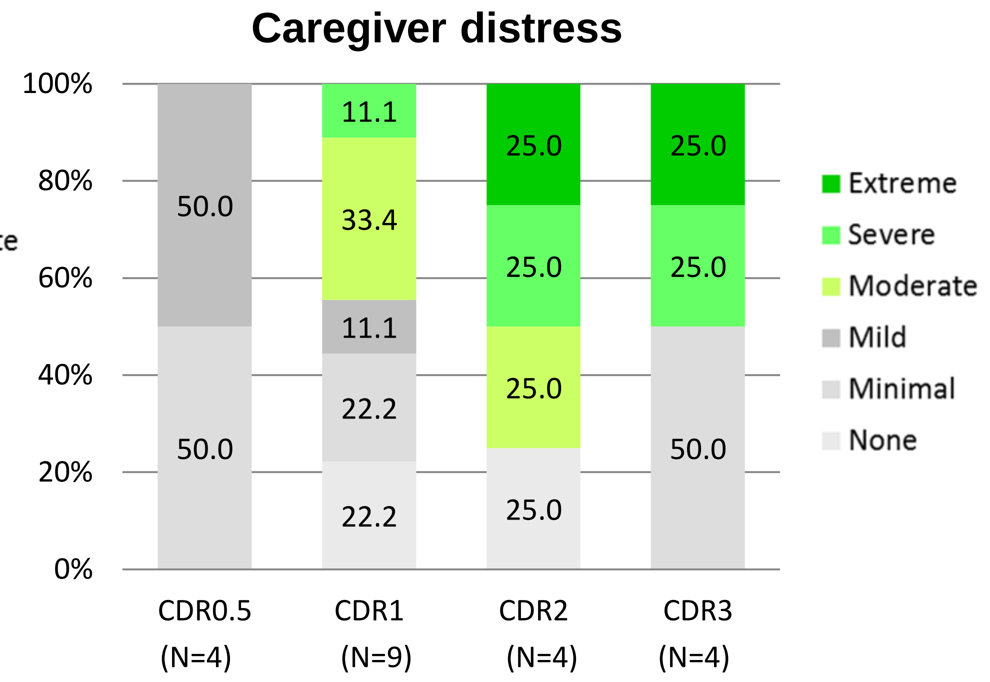

Agitation

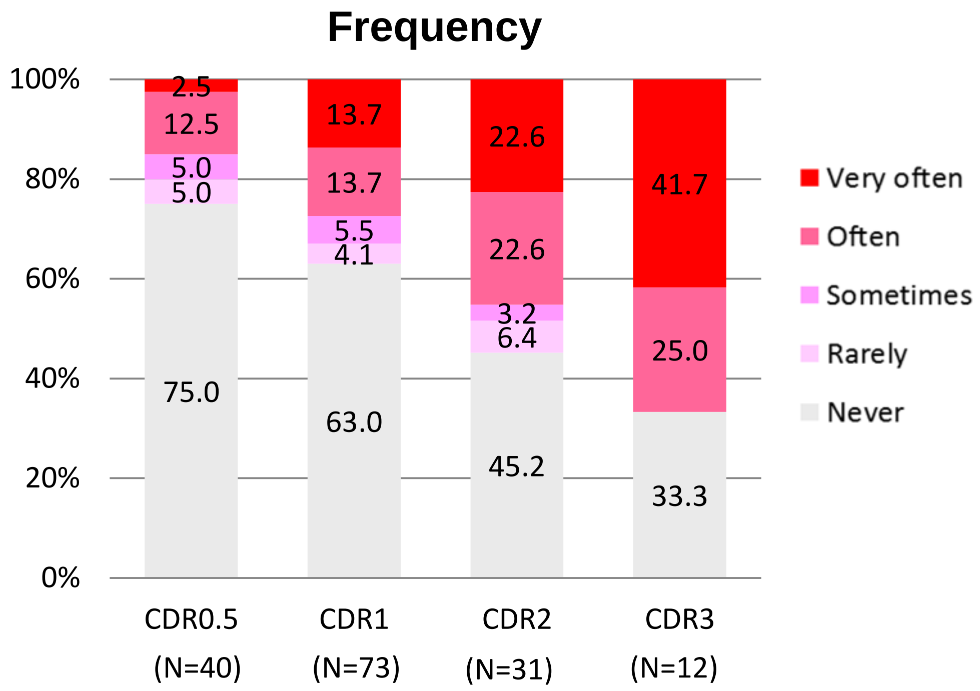

Frequency
100%
80%
60%
40%
20%
0%
2.5
12.5
5.0
5.0
75.0
13.7
13.7
5.5
4.1
63.0
22.6
22.6
3.2
6.4
45.2
41.7
25.0
33.3
CDR0.5 (N=40)
CDR1 (N=73)
CDR2 (N=31)
CDR3 (N=12)
Very often
Often
Sometimes
Rarely
Never

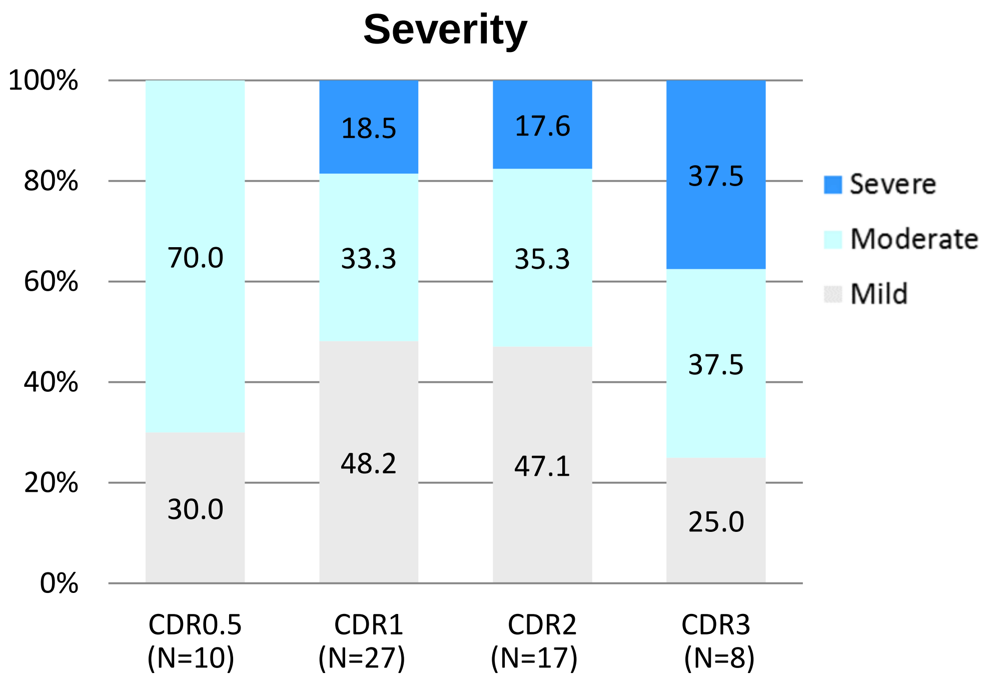

Severity
100%
80%
60%
40%
20%
0%
70.0
30.0
18.5
33.3
48.2
17.6
35.3
47.1
37.5
37.5
25.0
CDR0.5 (N=10)
CDR1 (N=27)
CDR2 (N=17)
CDR3 (N=8)
Severe
Moderate
Mild

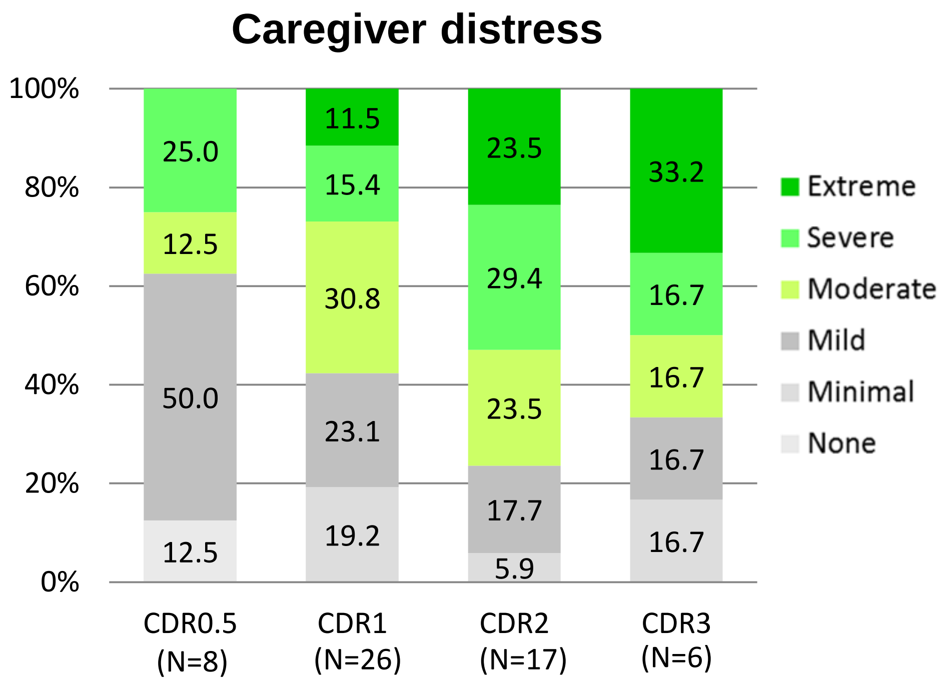

Caregiver distress
100%
80%
60%
40%
20%
0%
25.0
12.5
50.0
12.5
11.5
15.4
30.8
23.1
19.2
23.5
29.4
23.5
17.7
5.9
33.2
16.7
16.7
16.7
16.7
CDR0.5 (N=8)
CDR1 (N=26)
CDR2 (N=17)
CDR3 (N=6)
Extreme
Severe
Moderate
Mild
Minimal
None

Depression

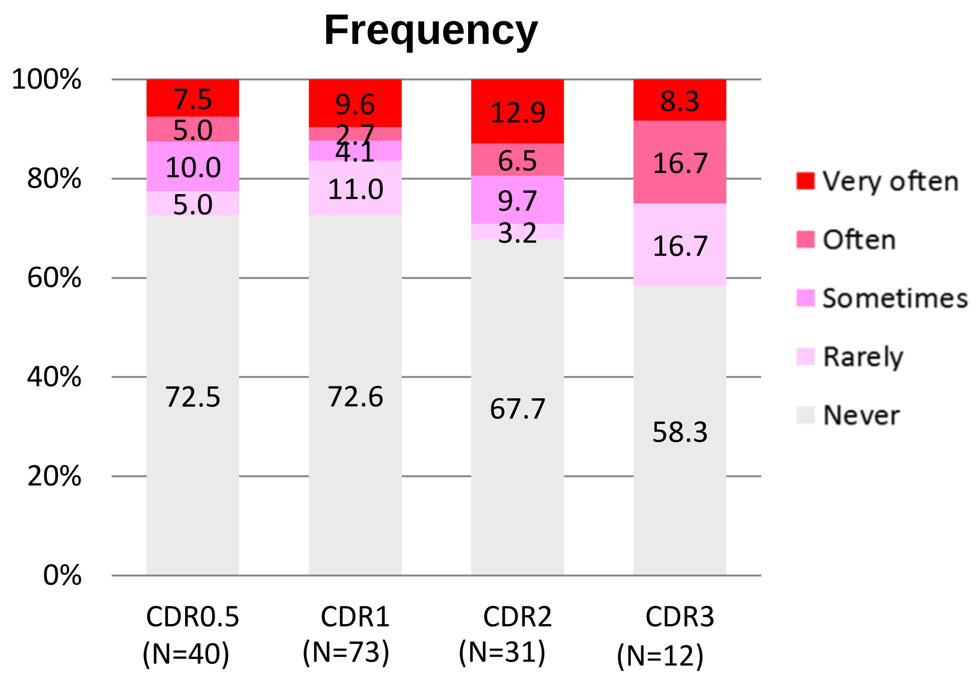

Frequency
100%
80%
60%
40%
20%
0%
7.5
5.0
10.0
5.0
72.5
9.6
2.7
4.1
11.0
72.6
12.9
6.5
9.7
3.2
67.7
8.3
16.7
16.7
58.3
CDR0.5 (N=40)
CDR1 (N=73)
CDR2 (N=31)
CDR3 (N=12)
Very often
Often
Sometimes
Rarely
Never

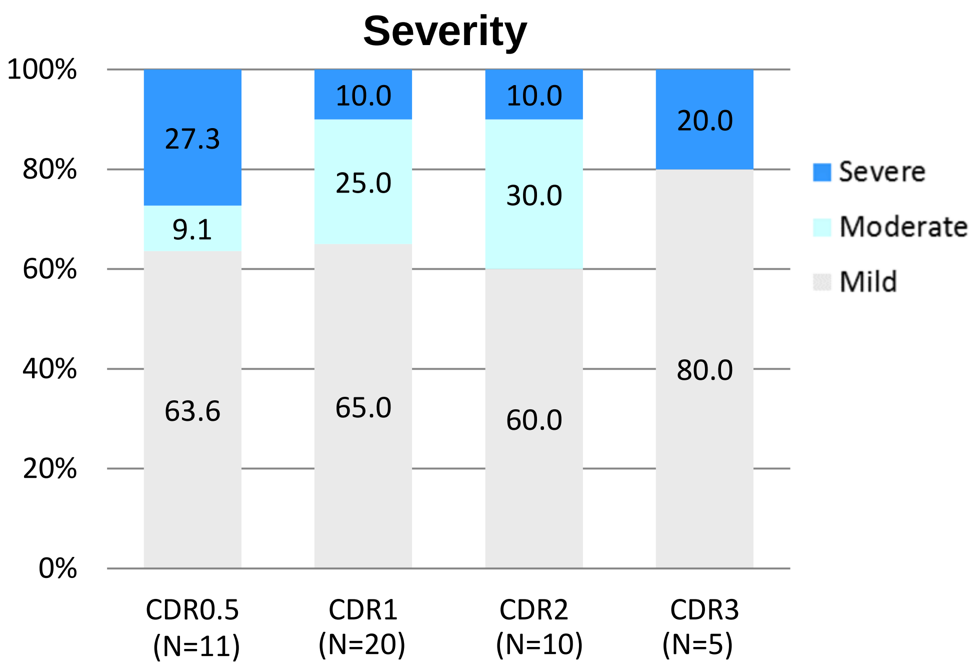

Severity
100%
80%
60%
40%
20%
0%
27.3
9.1
63.6
10.0
25.0
65.0
10.0
30.0
60.0
20.0
80.0
CDR0.5 (N=11)
CDR1 (N=20)
CDR2 (N=10)
CDR3 (N=5)
Severe
Moderate
Mild

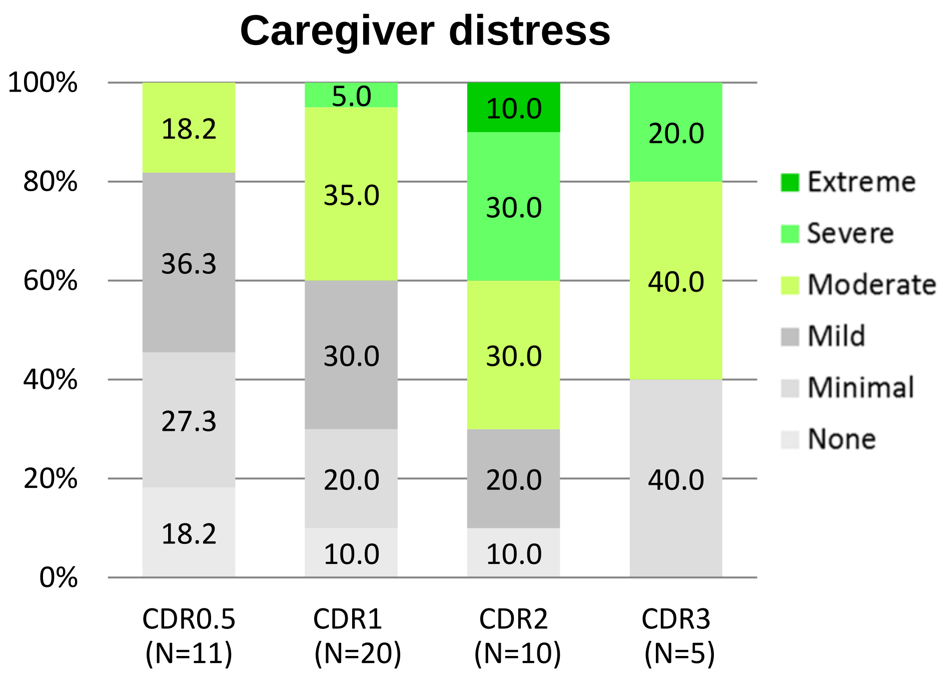

Caregiver distress
100%
80%
60%
40%
20%
0%
18.2
36.3
27.3
18.2
5.0
35.0
30.0
20.0
10.0
10.0
30.0
30.0
20.0
10.0
20.0
40.0
40.0
CDR0.5 (N=11)
CDR1 (N=20)
CDR2 (N=10)
CDR3 (N=5)
Extreme
Severe
Moderate
Mild
Minimal
None

Anxiety

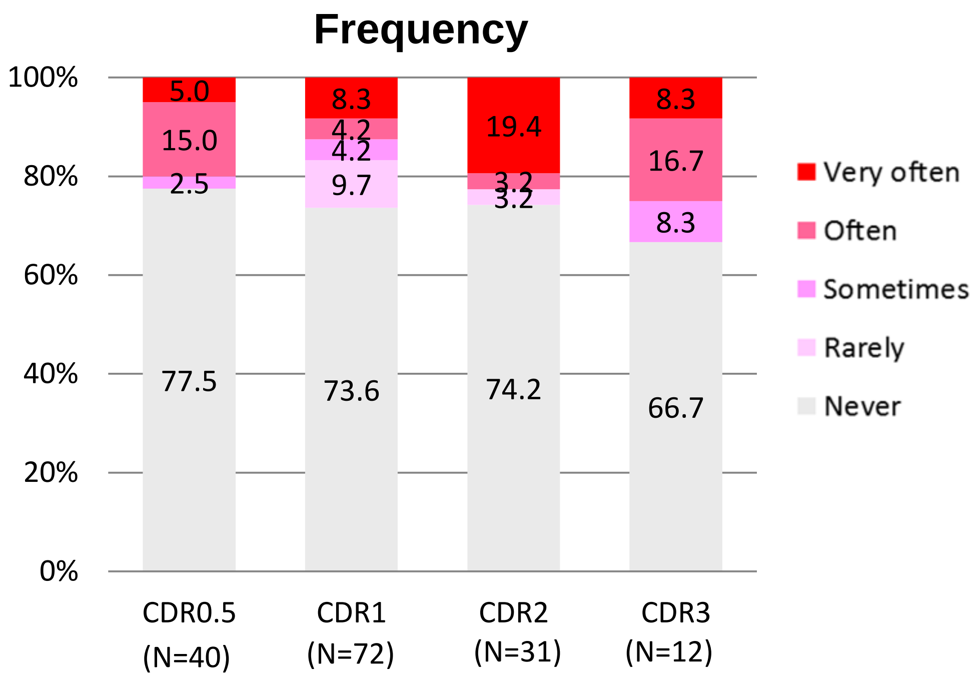
Frequency
100%
80%
60%
40%
20%
0%
5.0
15.0
2.5
77.5
8.3
4.2
4.2
9.7
73.6
19.4
3.2
3.2
74.2
8.3
16.7
8.3
66.7
CDR0.5 (N=40)
CDR1 (N=72)
CDR2 (N=31)
CDR3 (N=12)
Very often
Often
Sometimes
Rarely
Never

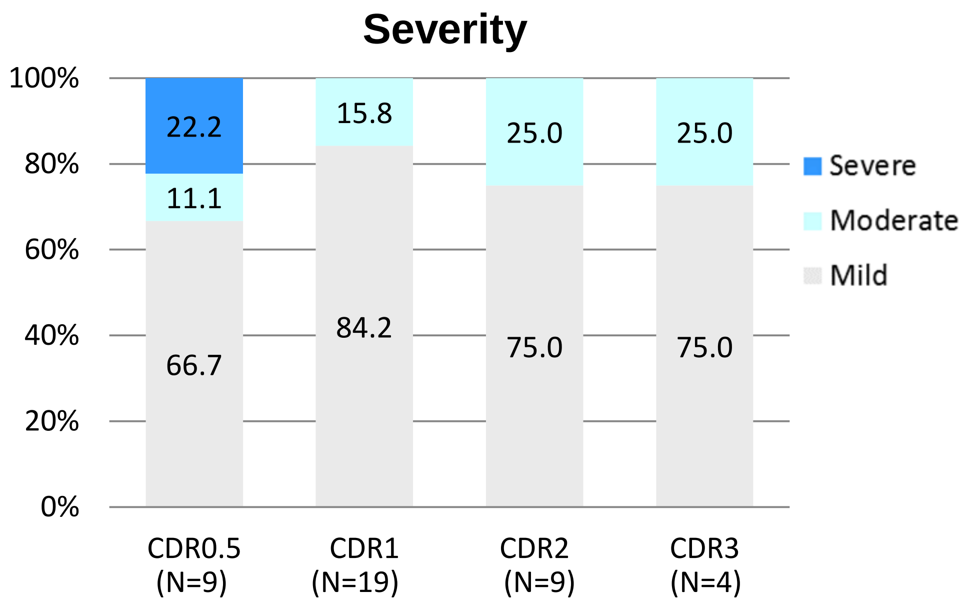
Severity
100%
80%
60%
40%
20%
0%
22.2
11.1
66.7
15.8
84.2
25.0
75.0
25.0
75.0
CDR0.5 (N=9)
CDR1 (N=19)
CDR2 (N=9)
CDR3 (N=4)
Severe
Moderate
Mild

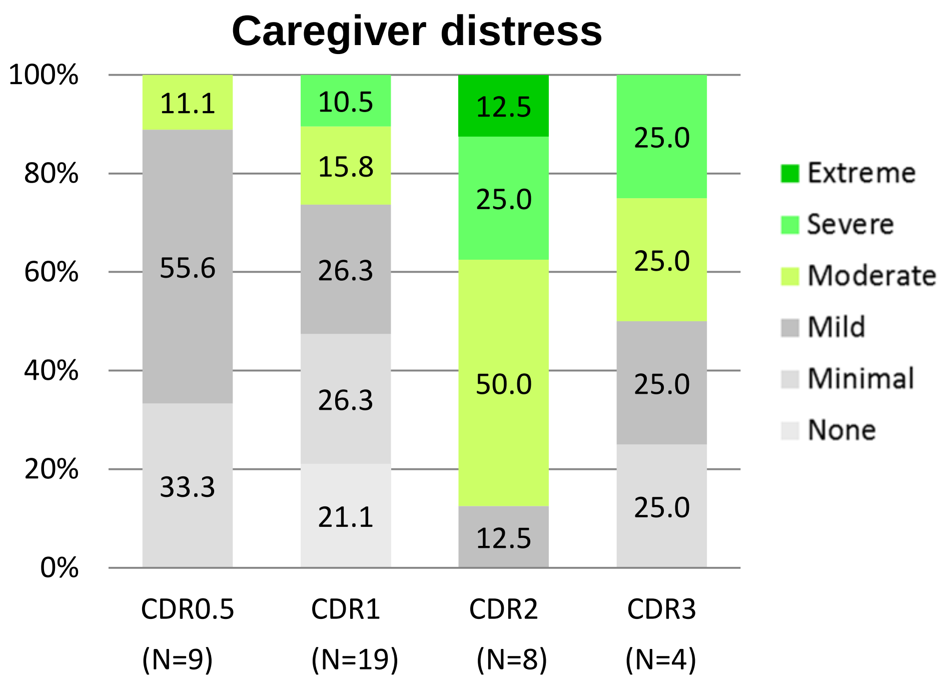
Caregiver distress
100%
80%
60%
40%
20%
0%
11.1
55.6
33.3
10.5
15.8
26.3
26.3
21.1
12.5
25.0
50.0
12.5
25.0
25.0
25.0
25.0
CDR0.5 (N=9)
CDR1 (N=19)
CDR2 (N=8)
CDR3 (N=4)
Extreme
Severe
Moderate
Mild
Minimal
None

Euphoria

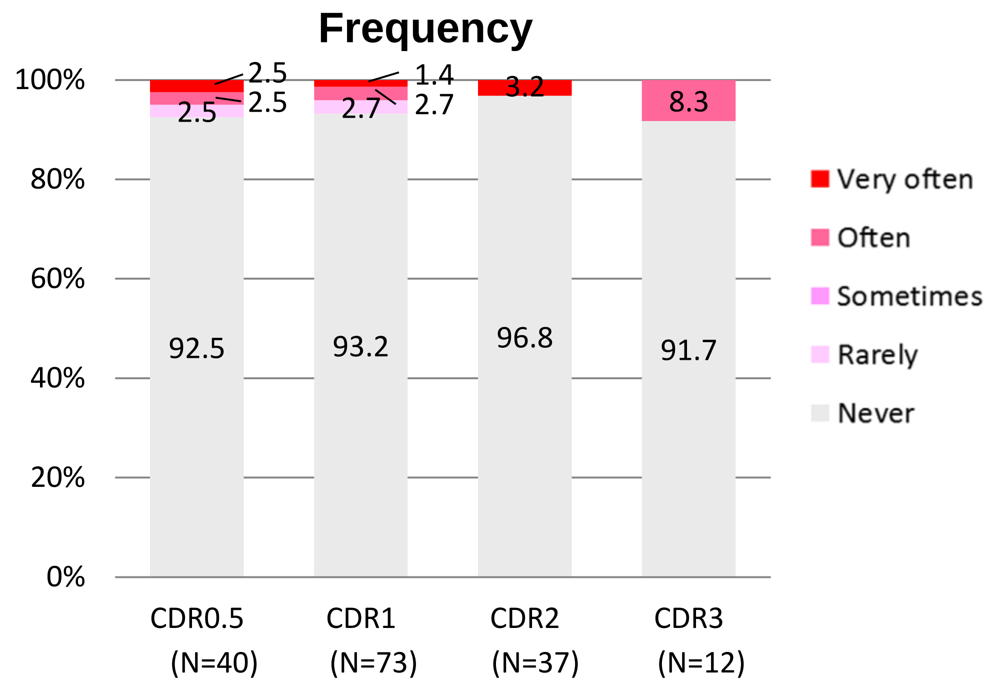
Frequency
100%
80%
60%
40%
20%
0%
2.5
2.5
2.5
92.5
1.4
2.7
2.7
93.2
3.2
96.8
8.3
91.7
CDR0.5 (N=40)
CDR1 (N=73)
CDR2 (N=37)
CDR3 (N=12)
Very often
Often
Sometimes
Rarely
Never

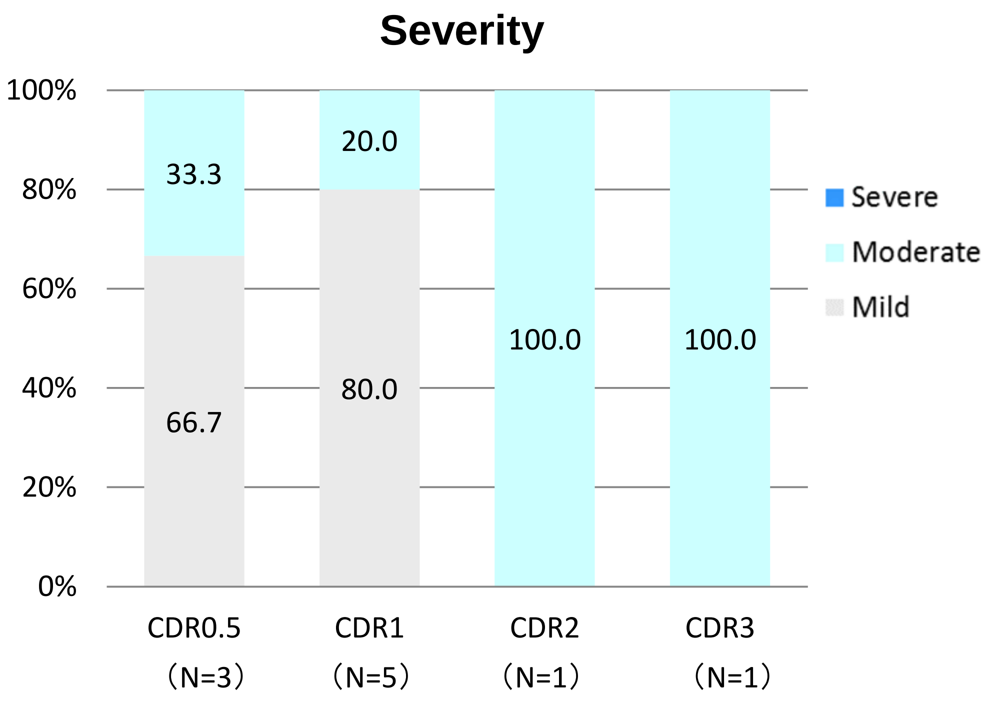
Severity
100%
80%
60%
40%
20%
0%
33.3
66.7
20.0
80.0
100.0
100.0
CDR0.5 （N=3）
CDR1 （N=5）
CDR2 （N=1）
CDR3 （N=1）
Severe
Moderate
Mild

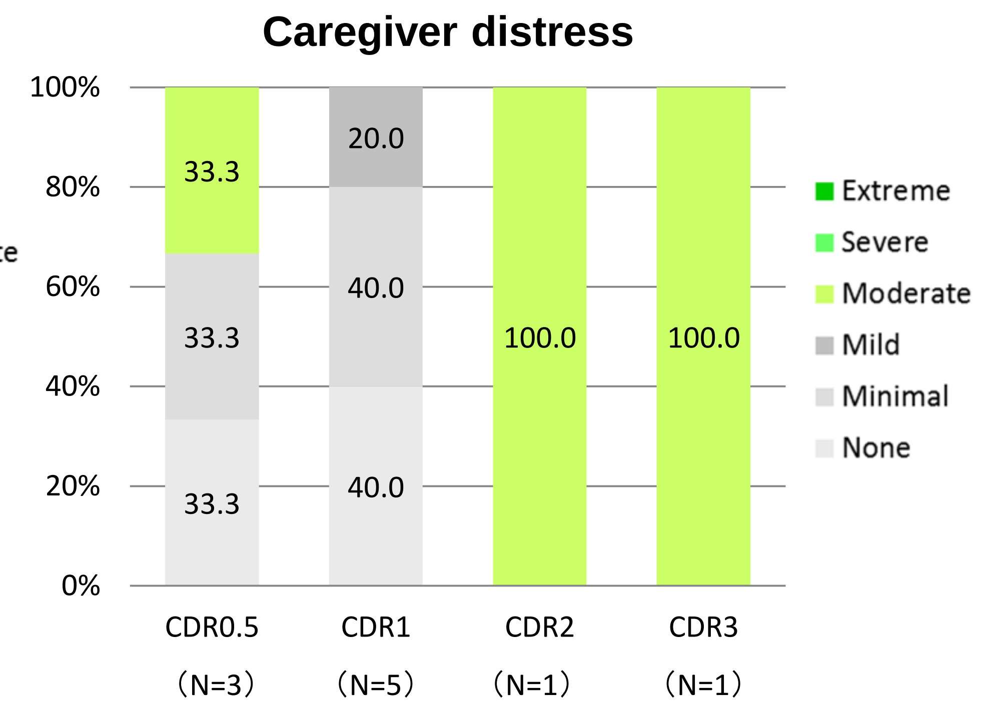
Caregiver distress
100%
80%
60%
40%
20%
0%
33.3
33.3
33.3
20.0
40.0
40.0
100.0
100.0
CDR0.5 （N=3）
CDR1 （N=5）
CDR2 （N=1）
CDR3 （N=1）
Extreme
Severe
Moderate
Mild
Minimal
None

Apathy

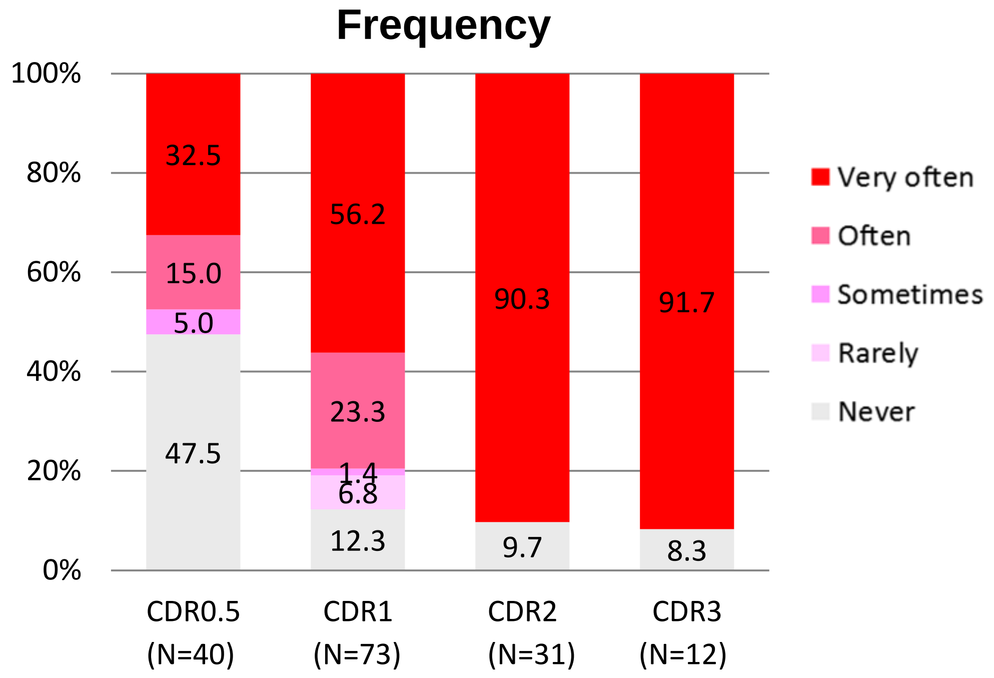
Frequency
100%
80%
60%
40%
20%
0%
32.5
15.0
5.0
47.5
56.2
23.3
1.4
6.8
12.3
90.3
9.7
91.7
8.3
CDR0.5 (N=40)
CDR1 (N=73)
CDR2 (N=31)
CDR3 (N=12)
Very often
Often
Sometimes
Rarely
Never

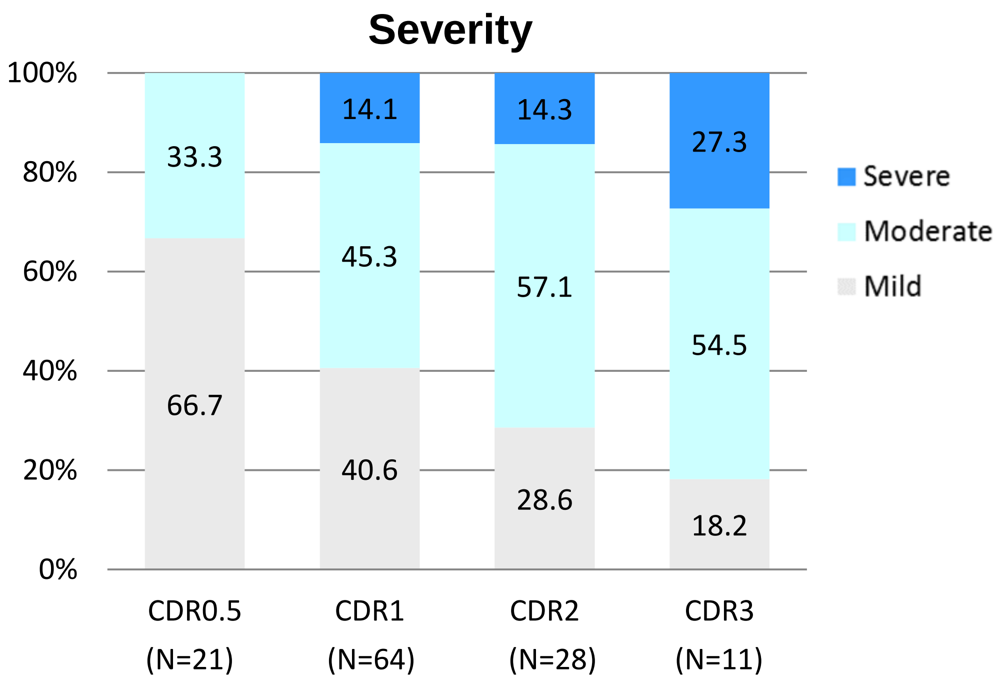
Severity
100%
80%
60%
40%
20%
0%
33.3
66.7
14.1
45.3
40.6
14.3
57.1
28.6
27.3
54.5
18.2
CDR0.5 (N=21)
CDR1 (N=64)
CDR2 (N=28)
CDR3 (N=11)
Severe
Moderate
Mild

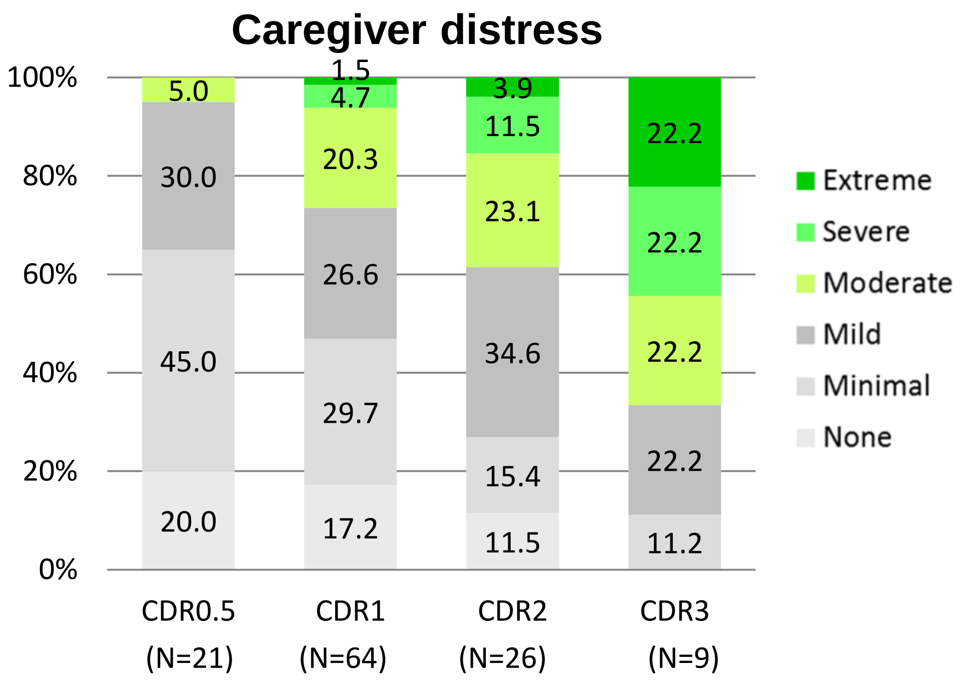
Caregiver distress
100%
80%
60%
40%
20%
0%
5.0
30.0
45.0
20.0
1.5
4.7
20.3
26.6
29.7
17.2
3.9
11.5
23.1
34.6
15.4
11.5
22.2
22.2
22.2
22.2
11.2
CDR0.5 (N=21)
CDR1 (N=64)
CDR2 (N=26)
CDR3 (N=9)
Extreme
Severe
Moderate
Mild
Minimal
None

Disinhibition

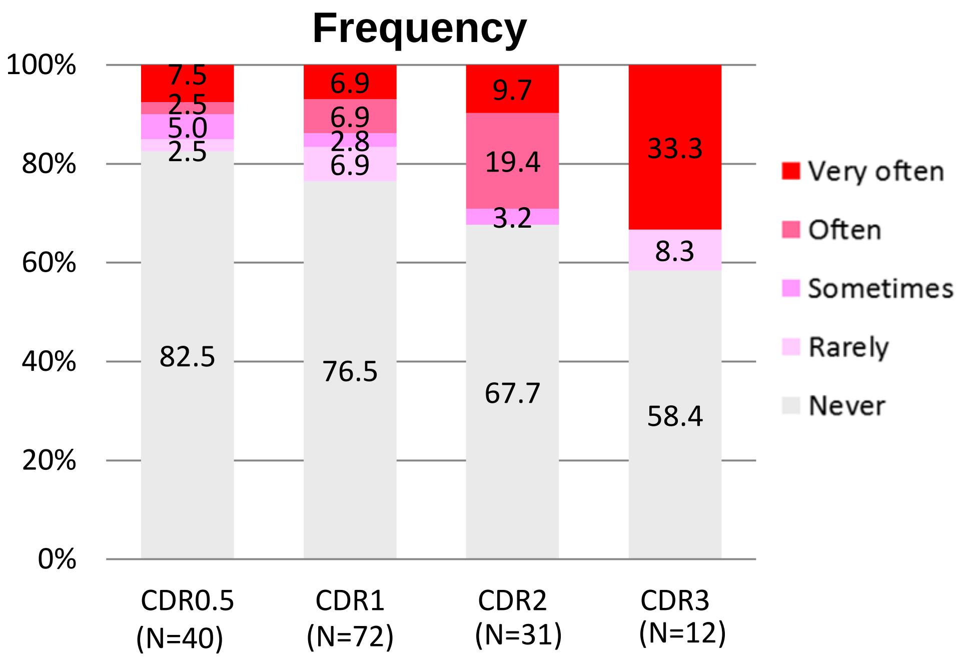

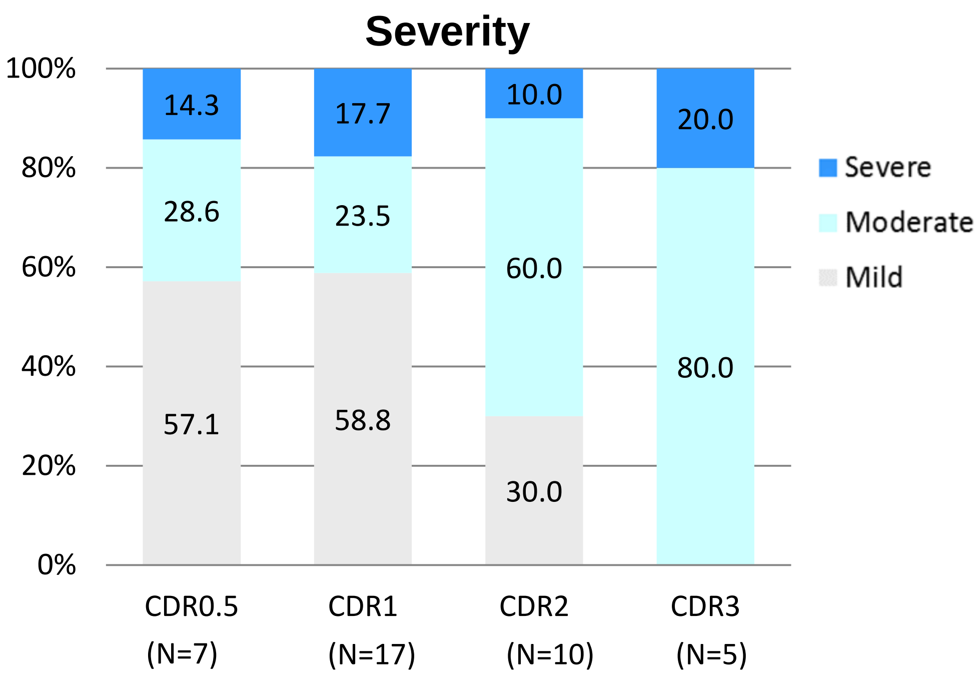

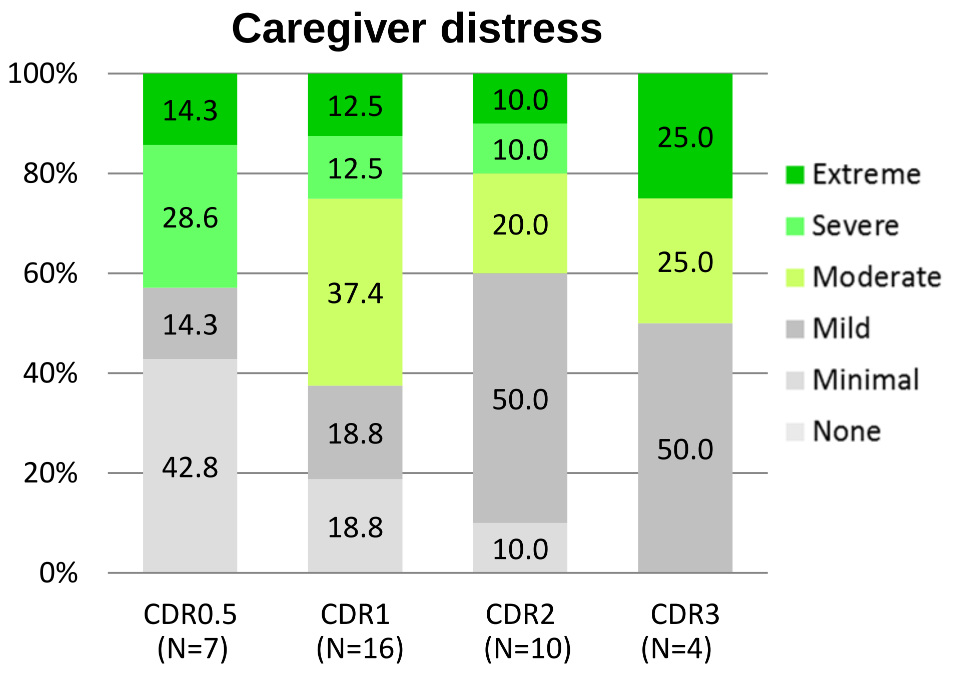

Irritability

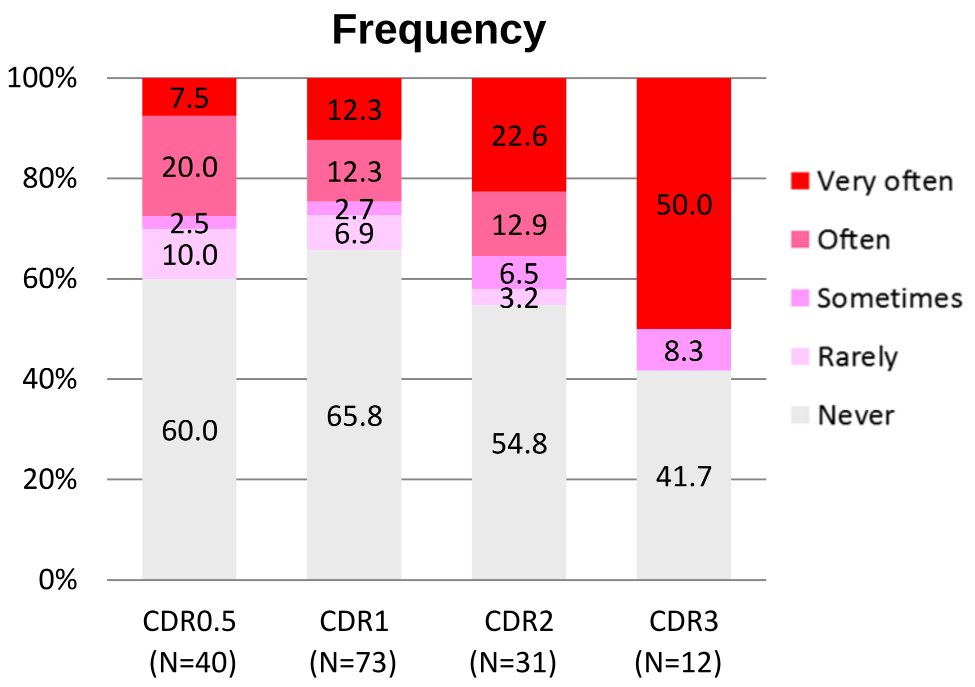

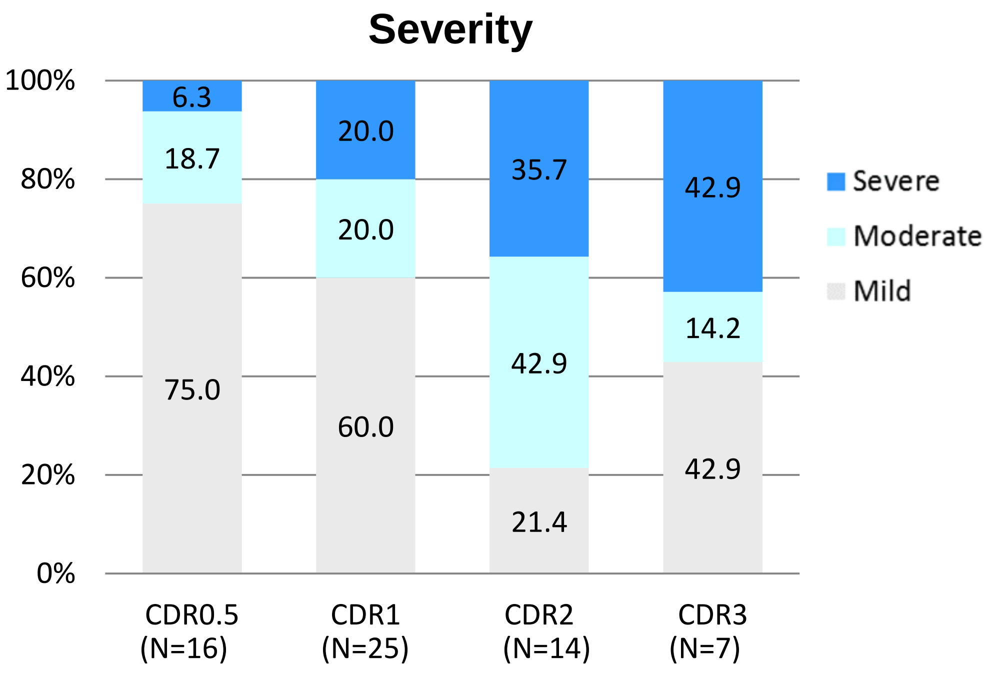

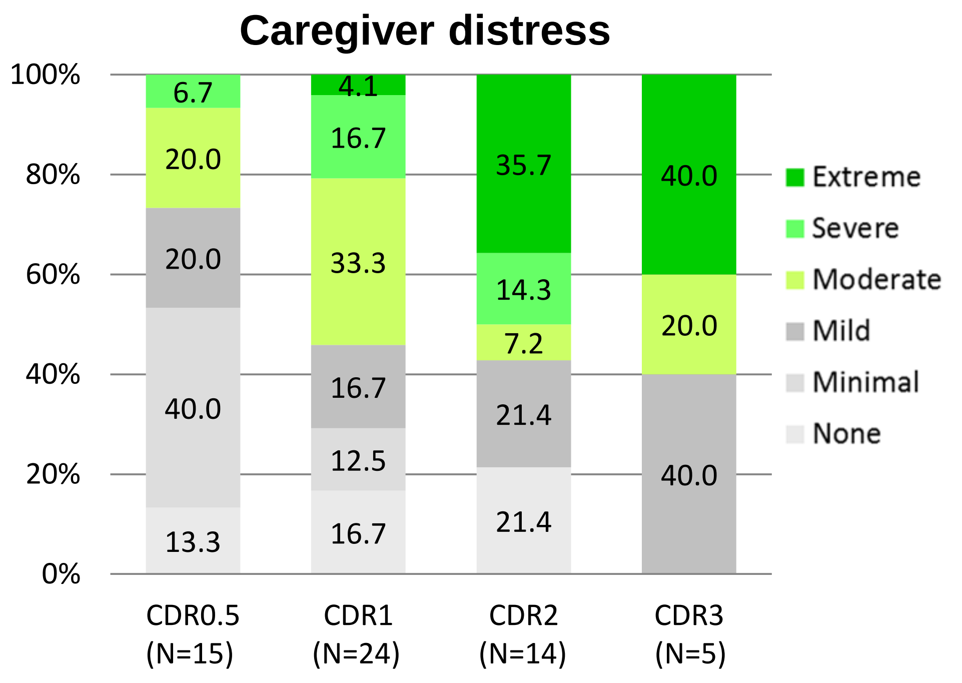

Aberrant motor behavior

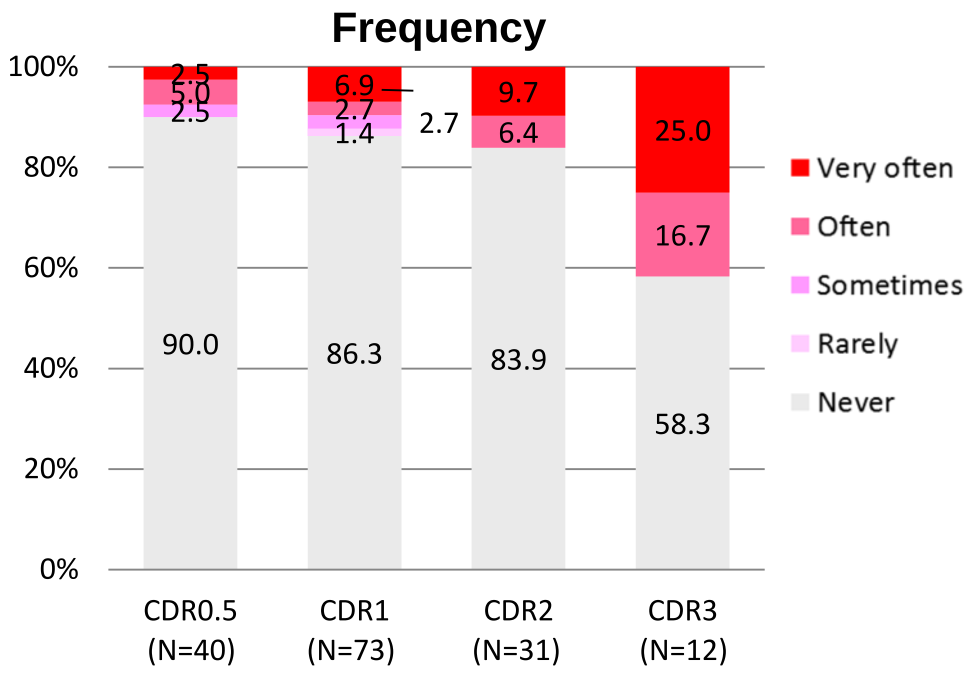

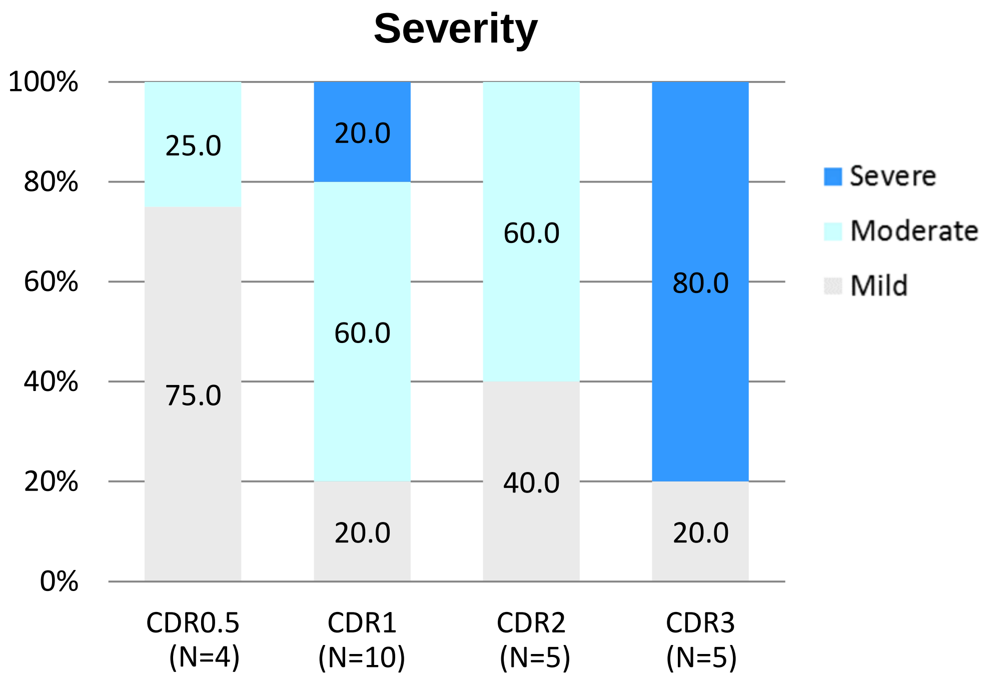

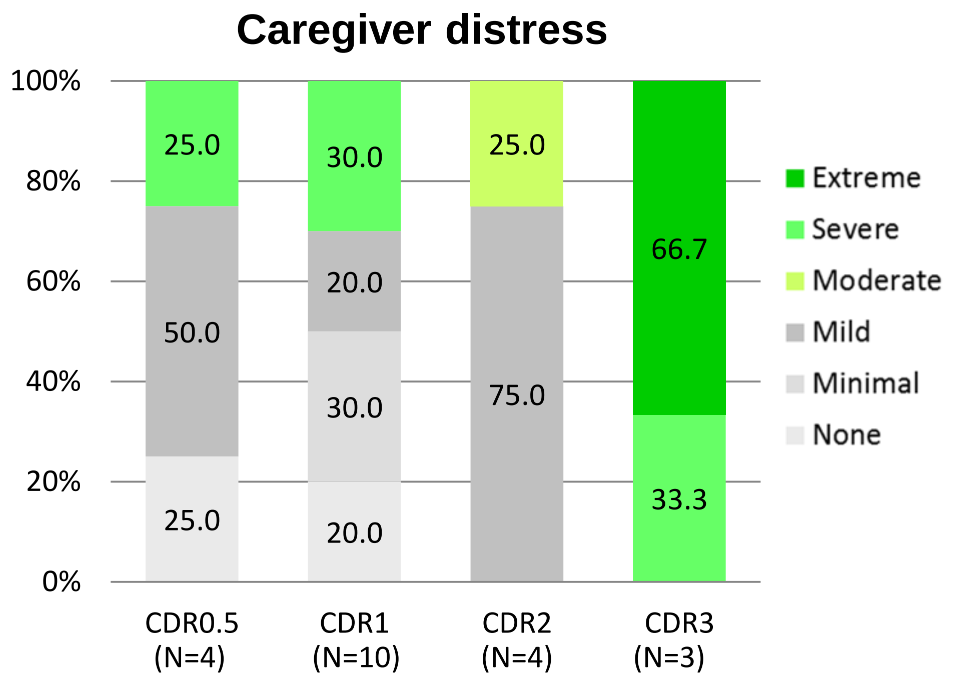

Sleep disturbances

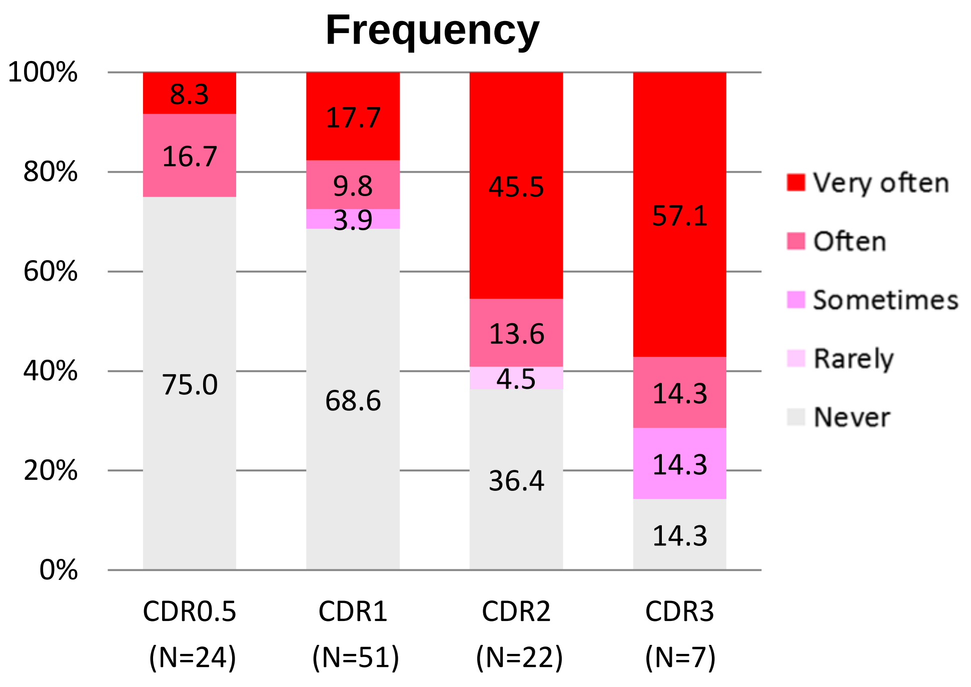

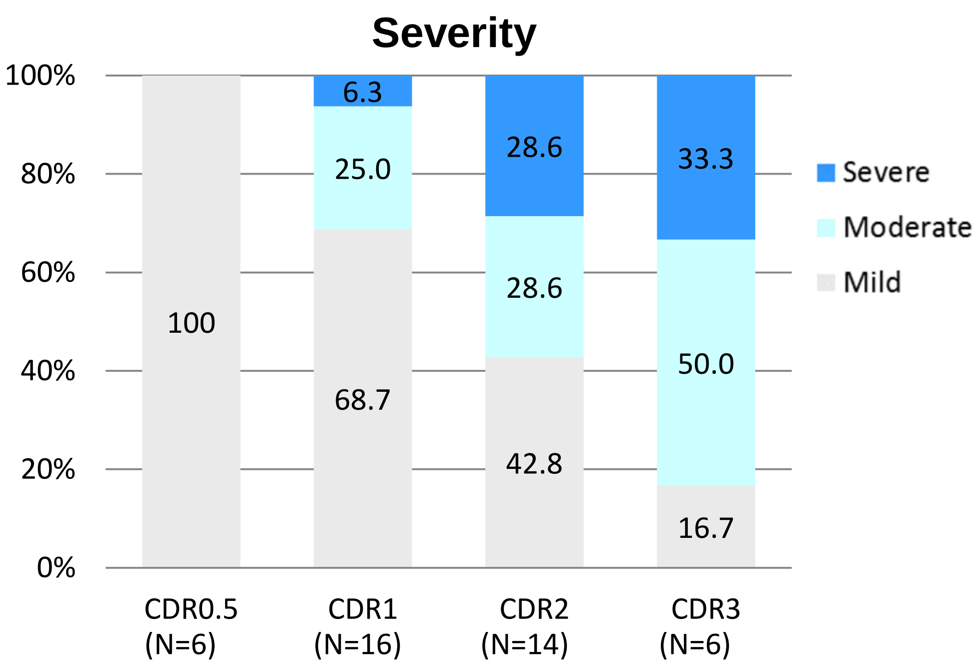

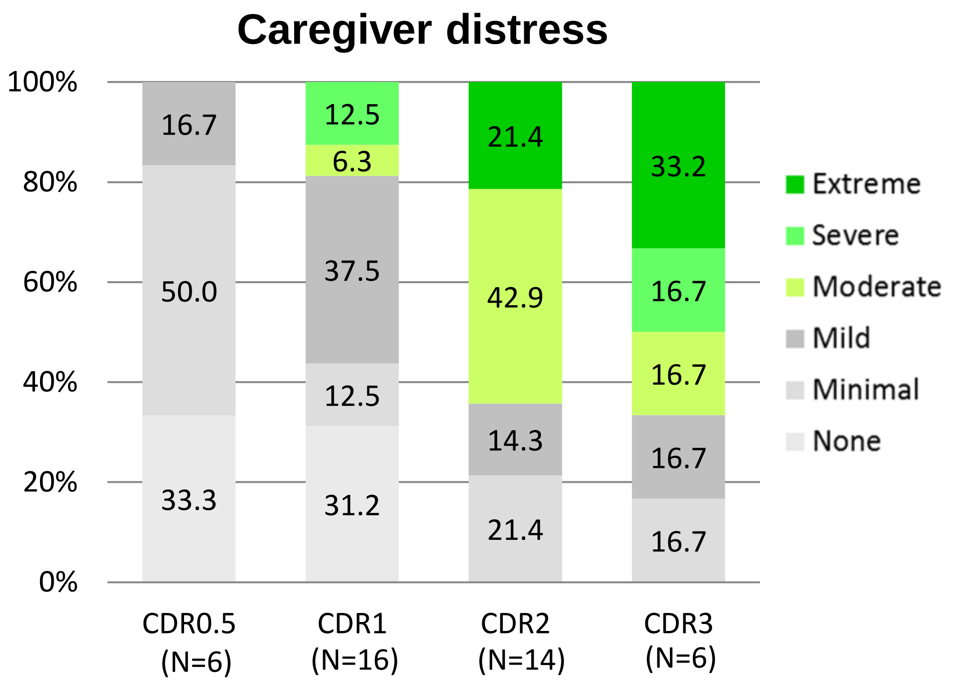

Eating abnormalities

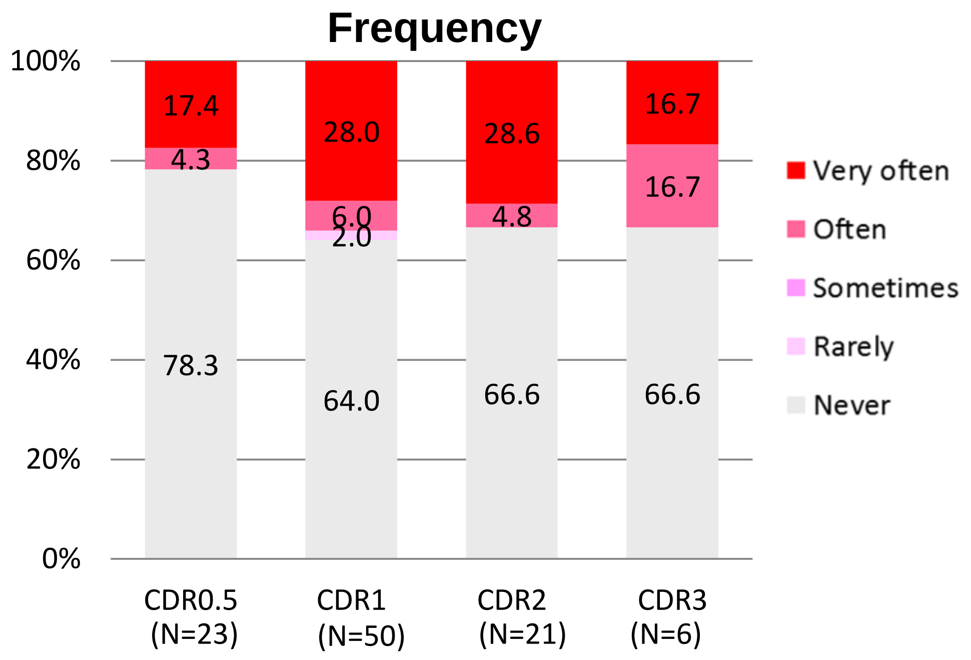

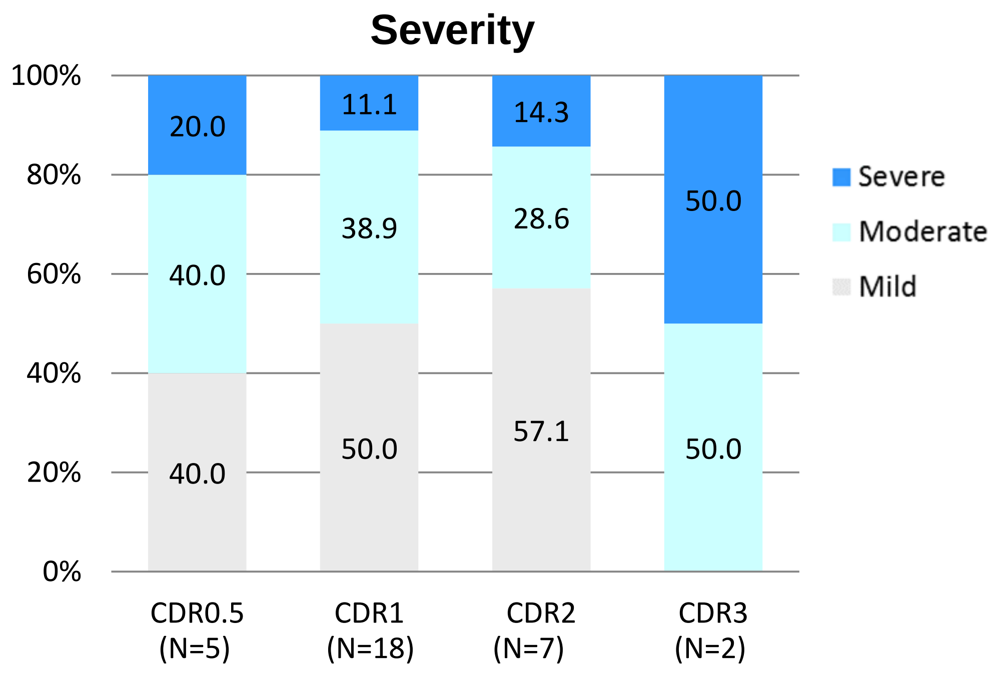

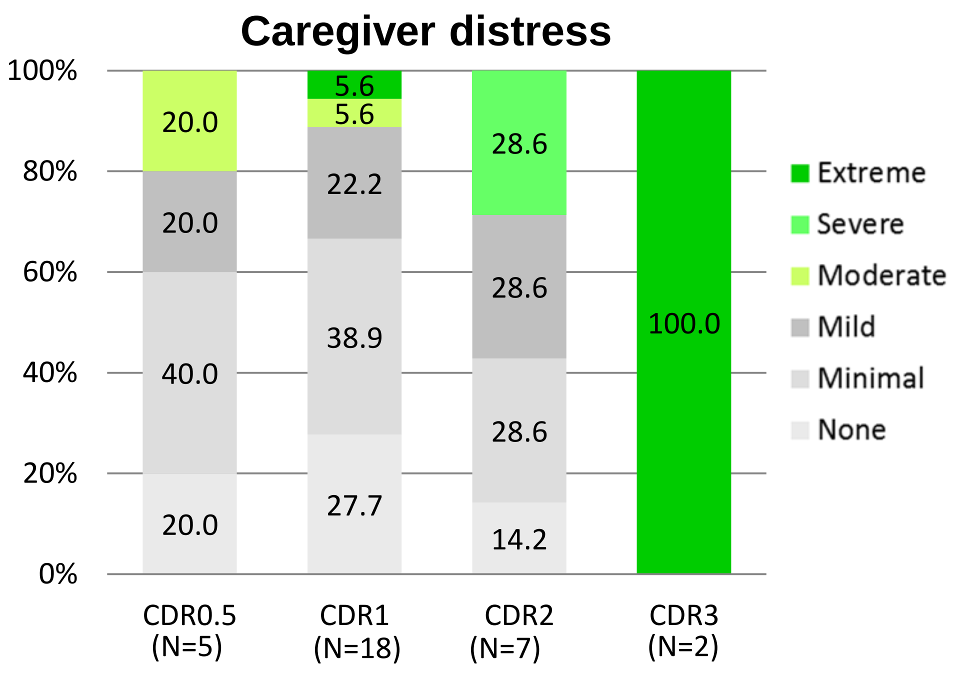

Supplement: S3 File — (PDF) [file pone.0161092.s003.pdf]
