# Supplementary material for: Differences of Behavioral and Psychological Symptoms of Dementia in Disease Severity in Four Major Dementias
Source: PLoS One. 2016 Aug 18;11(8):e0161092. doi: 10.1371/journal.pone.0161092 (PMC4990196; doi:10.1371/journal.pone.0161092)

S4 File.

Charts for BPSD in Frontotemporal lobar degeneration by disease severity

Delusions

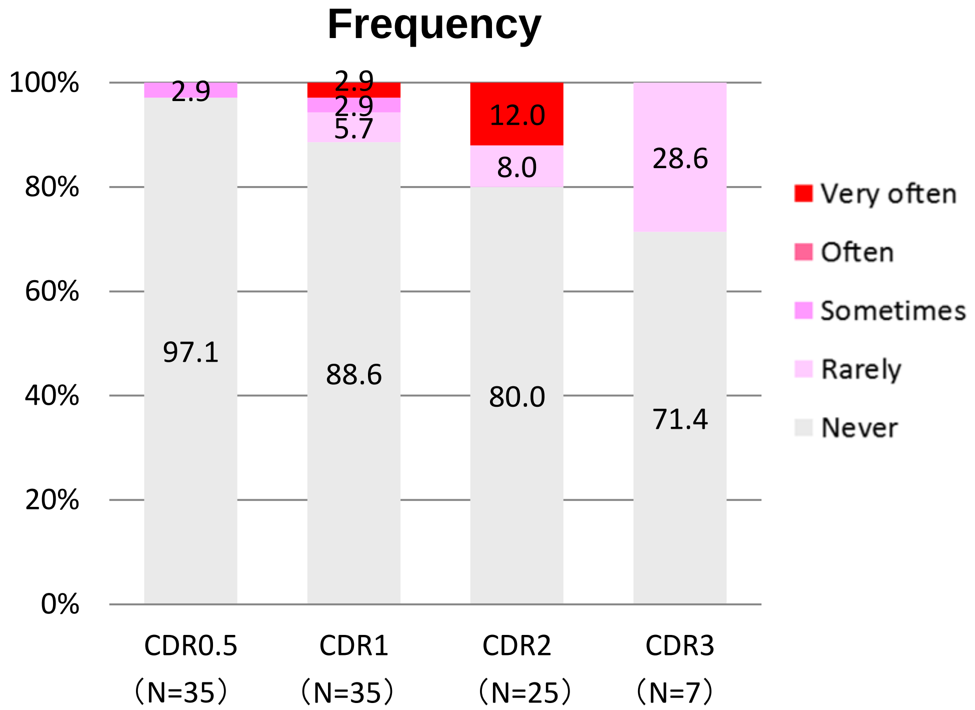
Frequency
100%
80%
60%
40%
20%
0%
2.9
97.1
2.9
2.9
5.7
88.6
12.0
8.0
80.0
28.6
71.4
CDR0.5 (N=35)
CDR1 (N=35)
CDR2 (N=25)
CDR3 (N=7)
Very often
Often
Sometimes
Rarely
Never

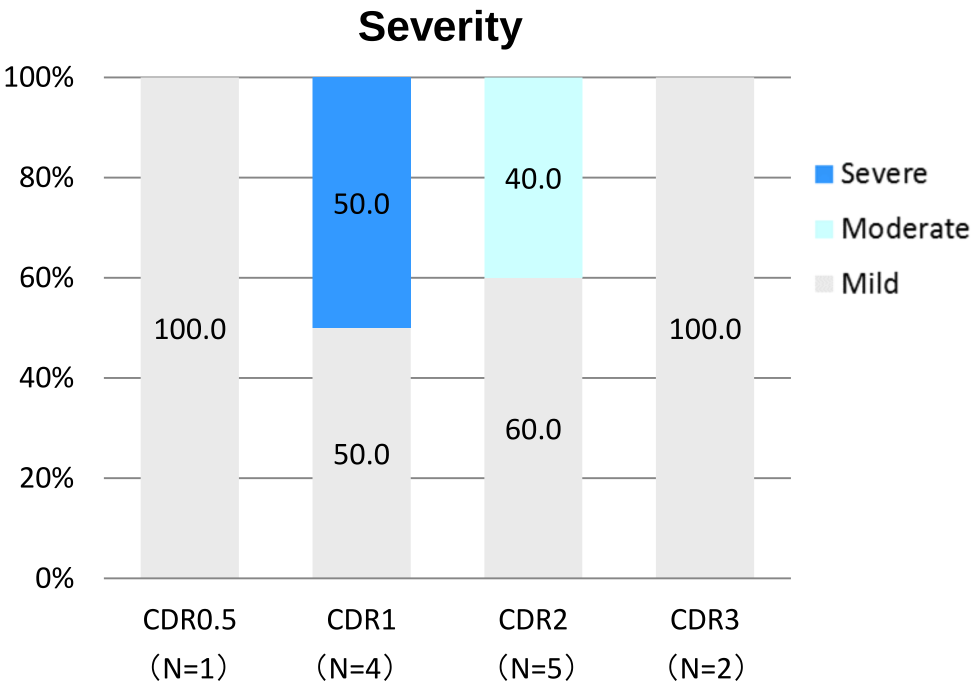
Severity
100%
80%
60%
40%
20%
0%
100.0
50.0
50.0
40.0
60.0
100.0
CDR0.5 (N=1)
CDR1 (N=4)
CDR2 (N=5)
CDR3 (N=2)
Severe
Moderate
Mild

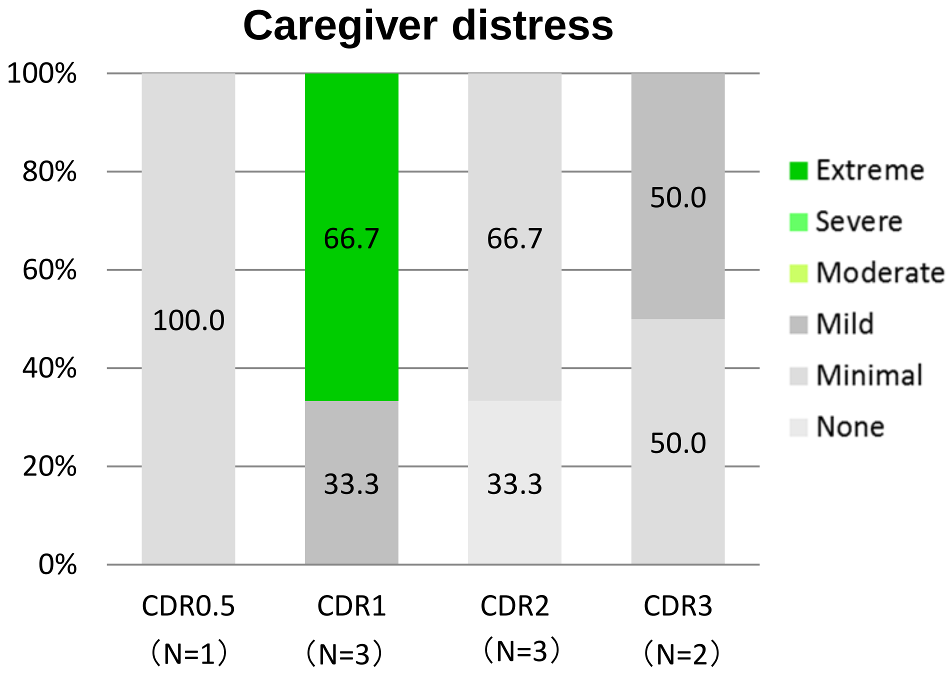
Caregiver distress
100%
80%
60%
40%
20%
0%
100.0
66.7
33.3
66.7
33.3
50.0
50.0
CDR0.5 (N=1)
CDR1 (N=3)
CDR2 (N=3)
CDR3 (N=2)
Extreme
Severe
Moderate
Mild
Minimal
None

Hallucinations

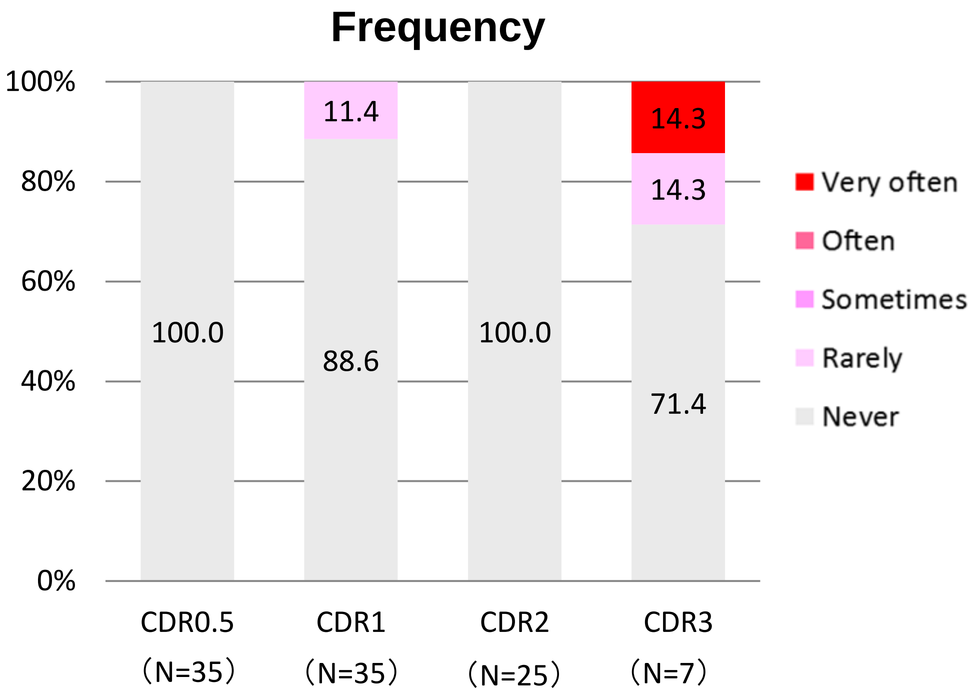

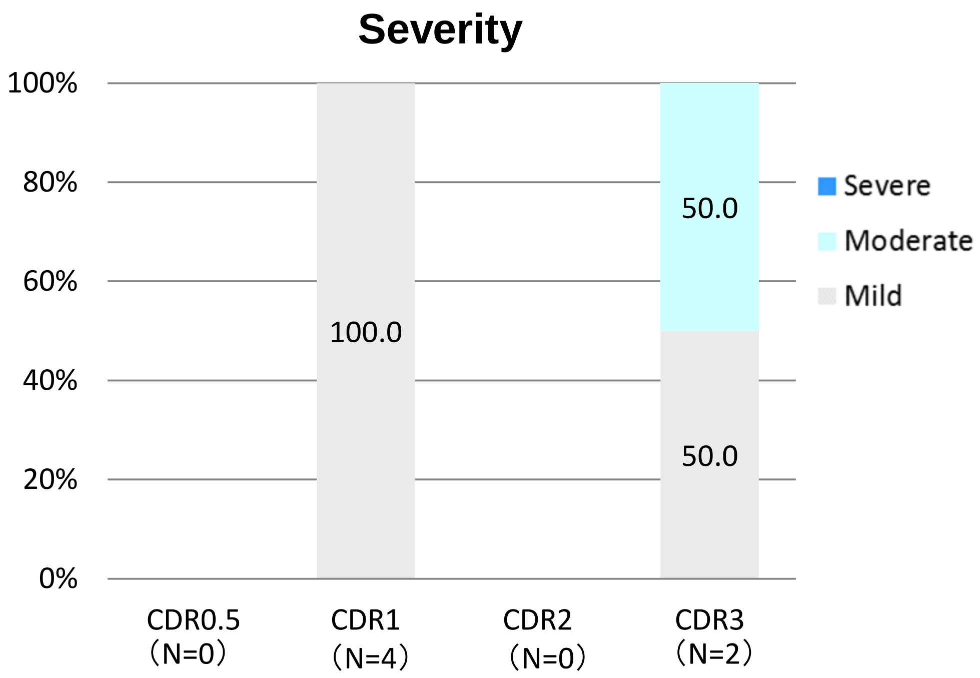

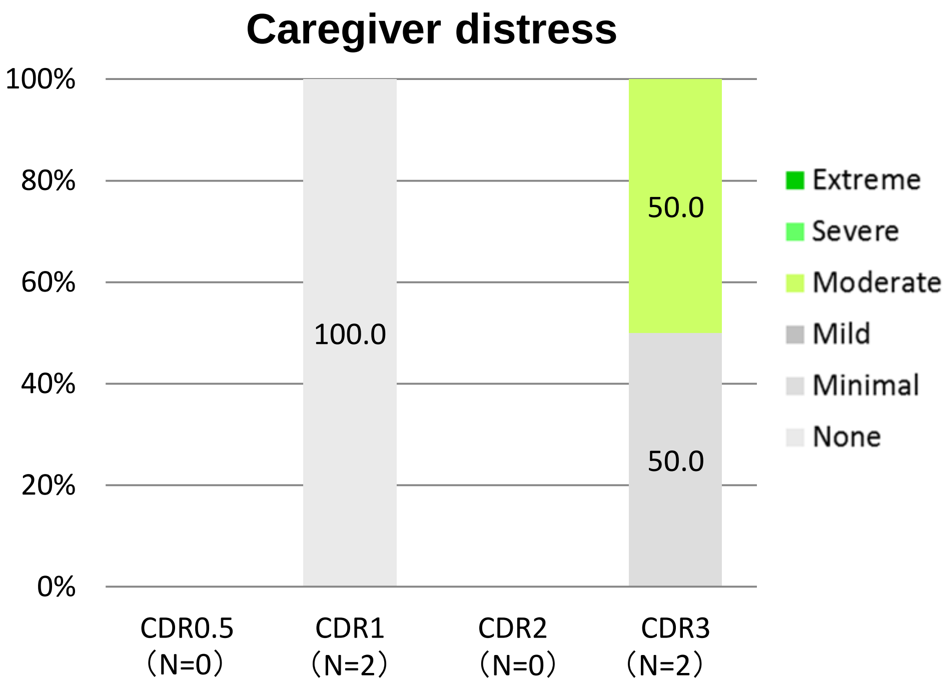

Agitation

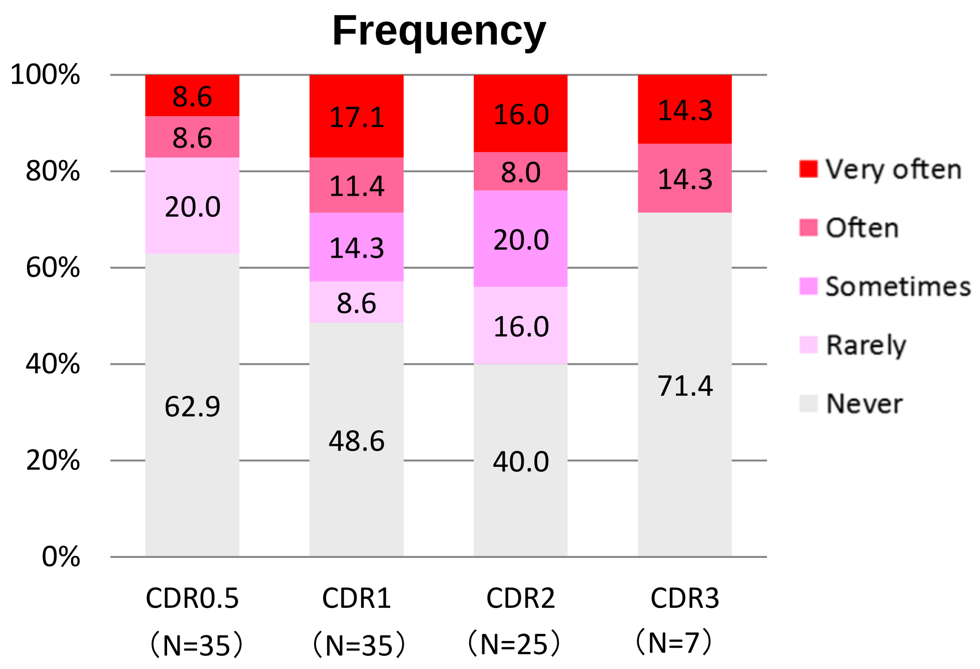
Frequency
100%
80%
60%
40%
20%
0%
8.6
8.6
20.0
62.9
17.1
11.4
14.3
8.6
48.6
16.0
8.0
20.0
16.0
40.0
14.3
14.3
71.4
Very often
Often
Sometimes
Rarely
Never
CDR0.5 (N=35)
CDR1 (N=35)
CDR2 (N=25)
CDR3 (N=7)

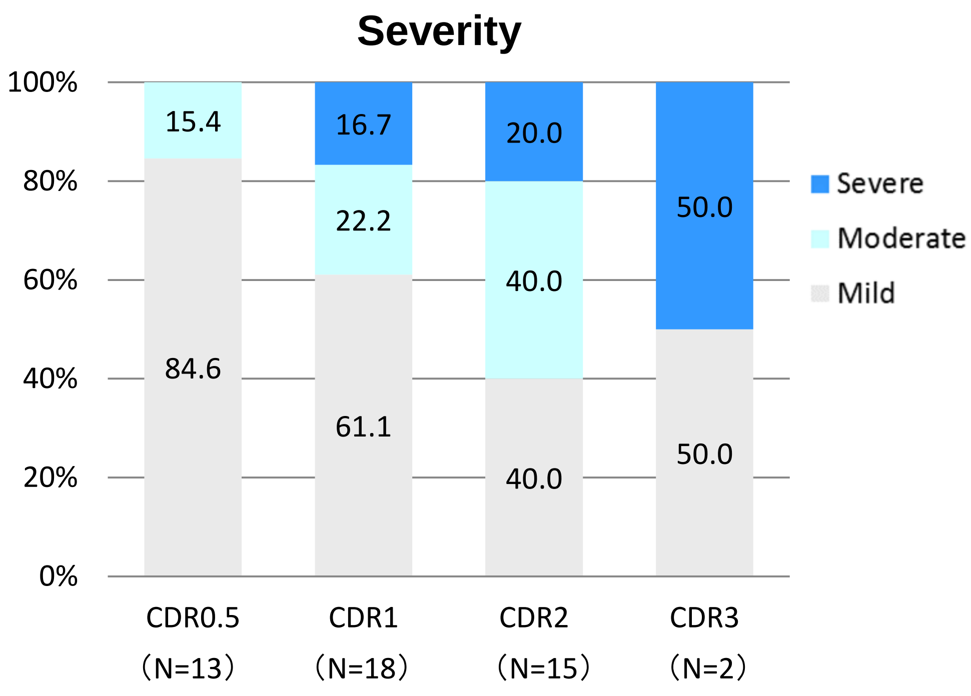
Severity
100%
80%
60%
40%
20%
0%
15.4
84.6
16.7
22.2
61.1
20.0
40.0
40.0
50.0
50.0
Severe
Moderate
Mild
CDR0.5 (N=13)
CDR1 (N=18)
CDR2 (N=15)
CDR3 (N=2)

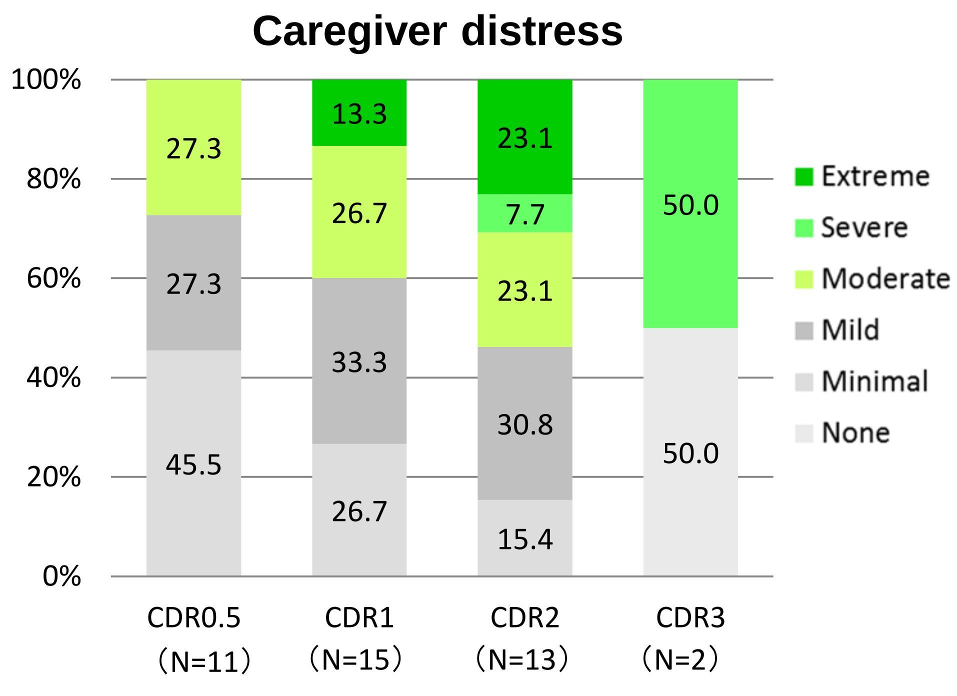
Caregiver distress
100%
80%
60%
40%
20%
0%
27.3
27.3
45.5
13.3
26.7
33.3
26.7
23.1
7.7
23.1
30.8
15.4
50.0
50.0
Extreme
Severe
Moderate
Mild
Minimal
None
CDR0.5 (N=11)
CDR1 (N=15)
CDR2 (N=13)
CDR3 (N=2)

Depression

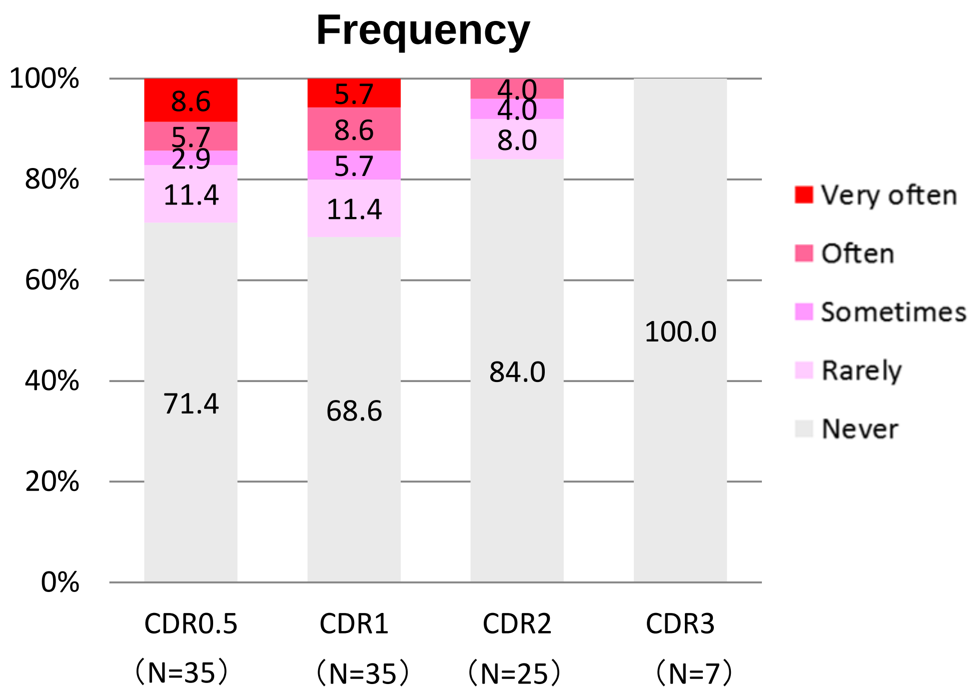
Frequency
100%
80%
60%
40%
20%
0%
8.6
5.7
2.9
11.4
71.4
5.7
8.6
5.7
11.4
68.6
4.0
4.0
8.0
84.0
100.0
CDR0.5 （N=35）
CDR1 （N=35）
CDR2 （N=25）
CDR3 （N=7）
Very often
Often
Sometimes
Rarely
Never

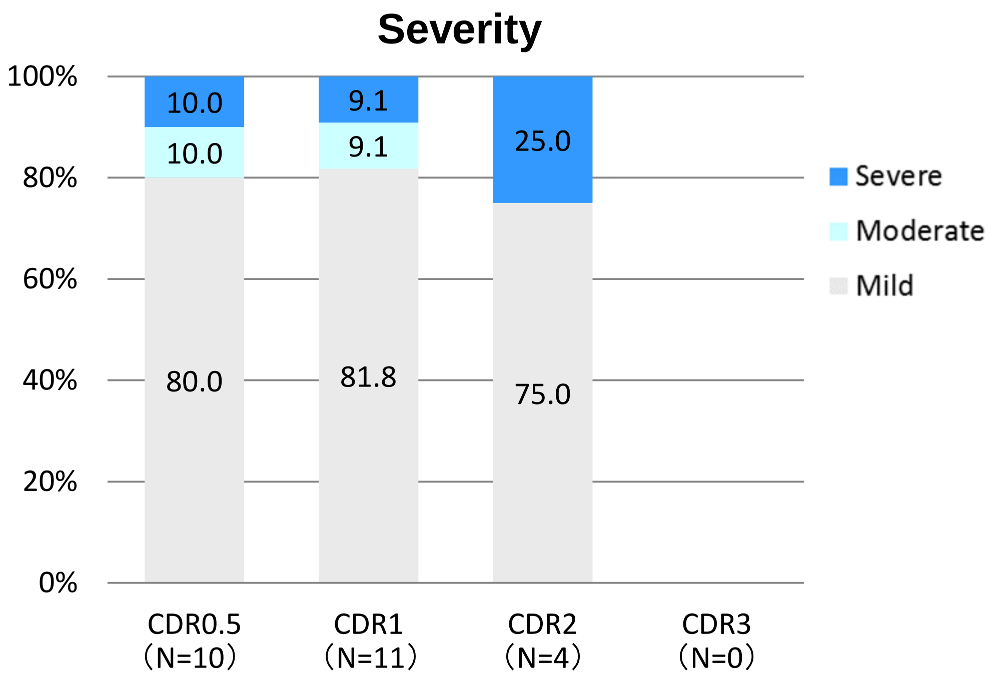
Severity
100%
80%
60%
40%
20%
0%
10.0
10.0
80.0
9.1
9.1
81.8
25.0
75.0
CDR0.5 （N=10）
CDR1 （N=11）
CDR2 （N=4）
CDR3 （N=0）
Severe
Moderate
Mild

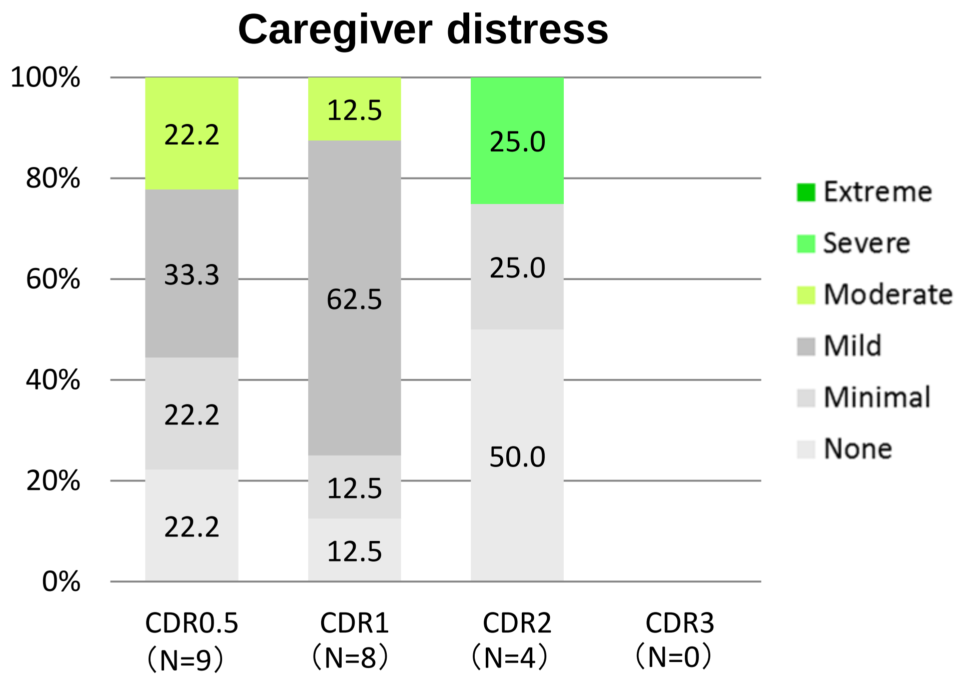
Caregiver distress
100%
80%
60%
40%
20%
0%
22.2
33.3
22.2
22.2
12.5
62.5
12.5
12.5
25.0
25.0
50.0
CDR0.5 （N=9）
CDR1 （N=8）
CDR2 （N=4）
CDR3 （N=0）
Extreme
Severe
Moderate
Mild
Minimal
None

Anxiety

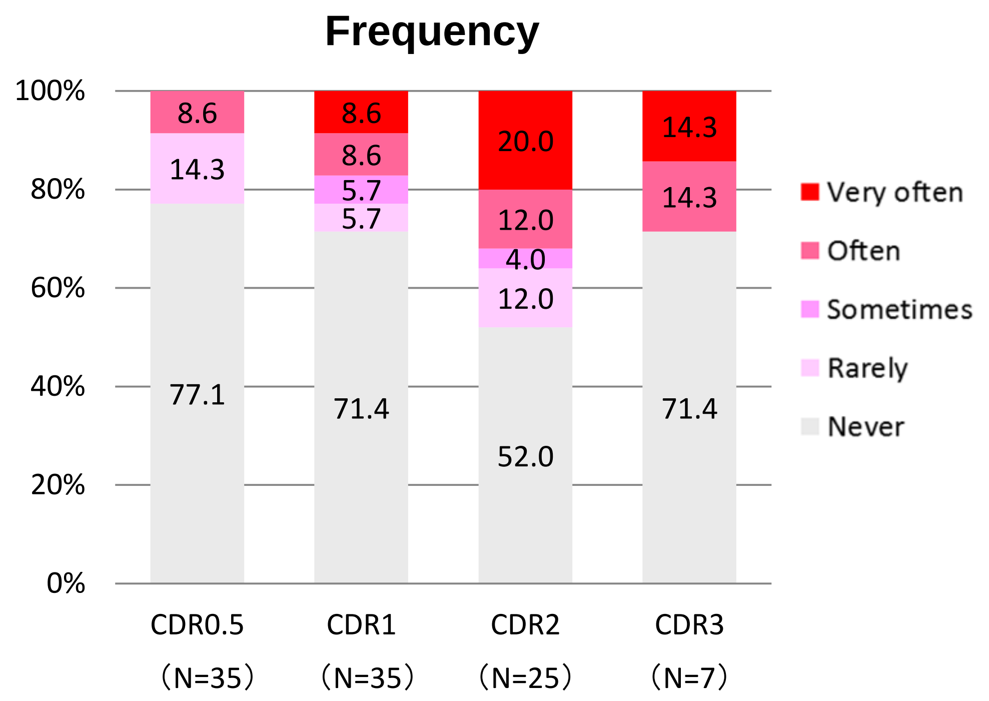
Frequency
100%
80%
60%
40%
20%
0%
8.6
14.3
77.1
8.6
8.6
5.7
5.7
71.4
20.0
12.0
4.0
12.0
52.0
14.3
14.3
71.4
Very often
Often
Sometimes
Rarely
Never
CDR0.5 (N=35)
CDR1 (N=35)
CDR2 (N=25)
CDR3 (N=7)

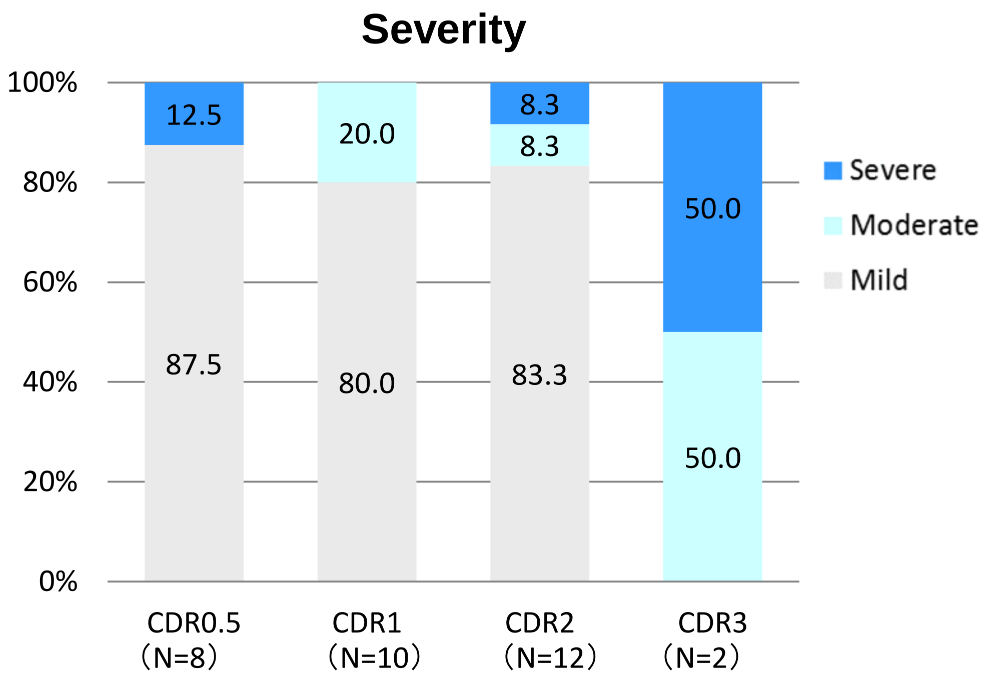
Severity
100%
80%
60%
40%
20%
0%
12.5
87.5
20.0
80.0
8.3
8.3
83.3
50.0
50.0
Severe
Moderate
Mild
CDR0.5 (N=8)
CDR1 (N=10)
CDR2 (N=12)
CDR3 (N=2)

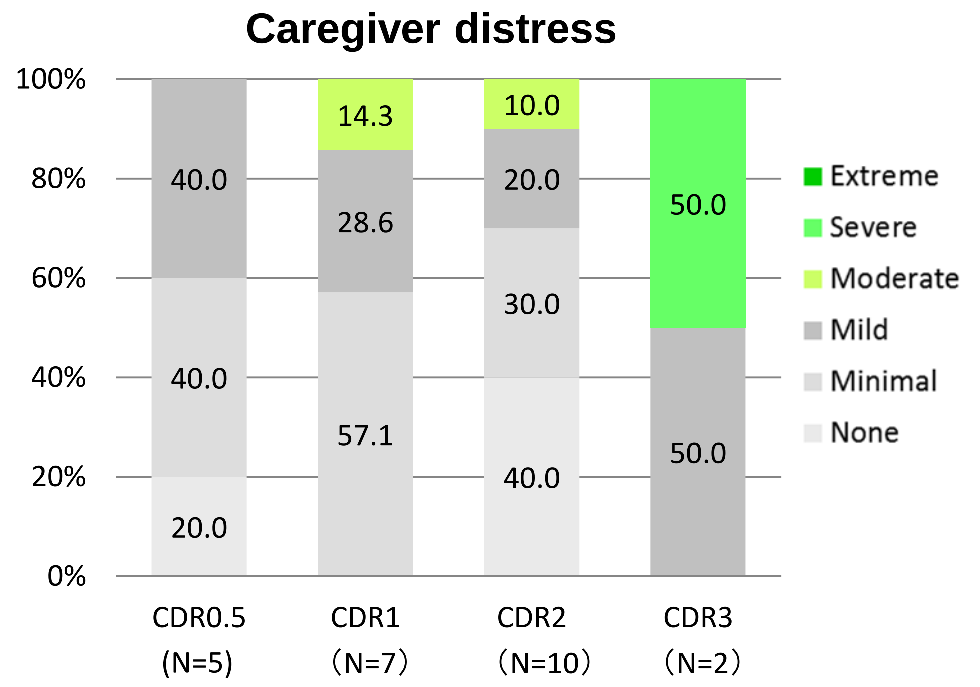
Caregiver distress
100%
80%
60%
40%
20%
0%
40.0
40.0
20.0
14.3
28.6
57.1
10.0
20.0
30.0
40.0
50.0
50.0
Extreme
Severe
Moderate
Mild
Minimal
None
CDR0.5 (N=5)
CDR1 (N=7)
CDR2 (N=10)
CDR3 (N=2)

Euphoria

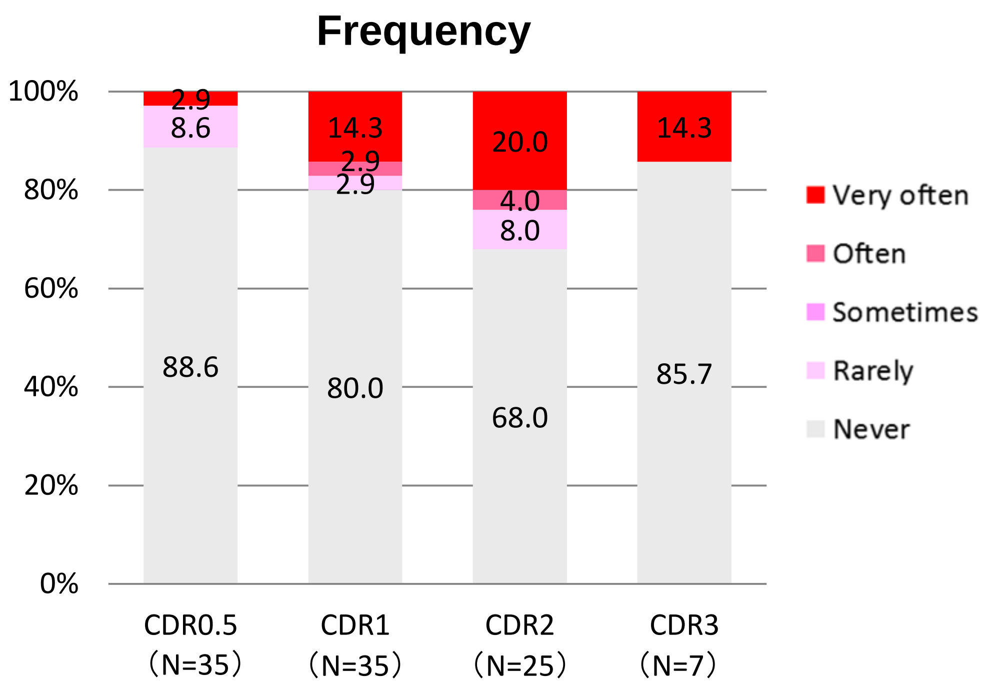
Frequency
100%
80%
60%
40%
20%
0%
2.9
8.6
88.6
14.3
2.9
2.9
80.0
20.0
4.0
8.0
68.0
14.3
85.7
CDR0.5 （N=35）
CDR1 （N=35）
CDR2 （N=25）
CDR3 （N=7）
Very often
Often
Sometimes
Rarely
Never

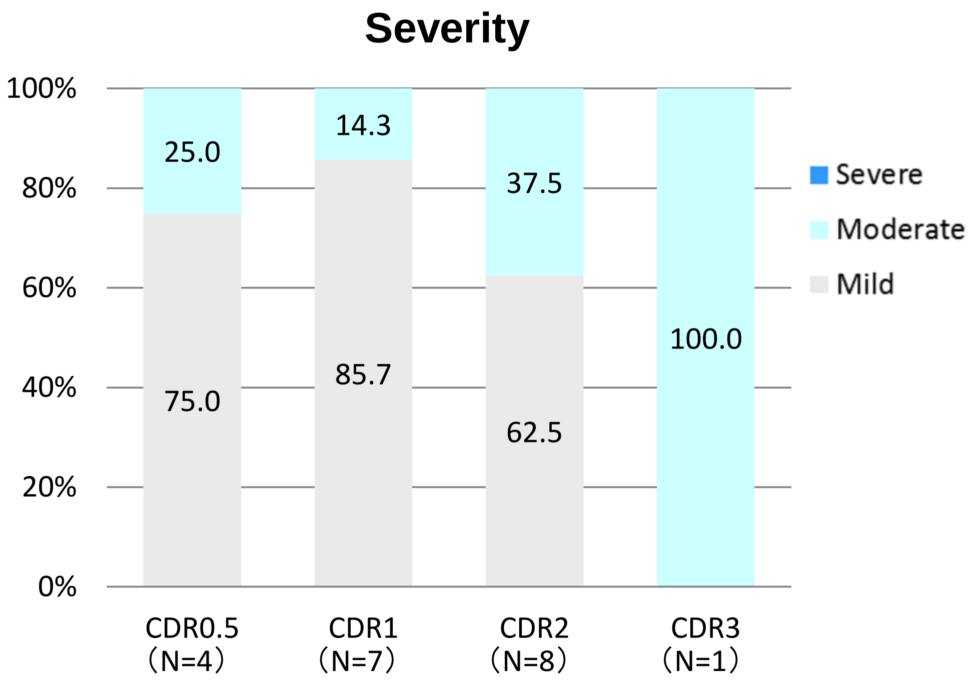
Severity
100%
80%
60%
40%
20%
0%
25.0
75.0
14.3
85.7
37.5
62.5
100.0
CDR0.5 （N=4）
CDR1 （N=7）
CDR2 （N=8）
CDR3 （N=1）
Severe
Moderate
Mild

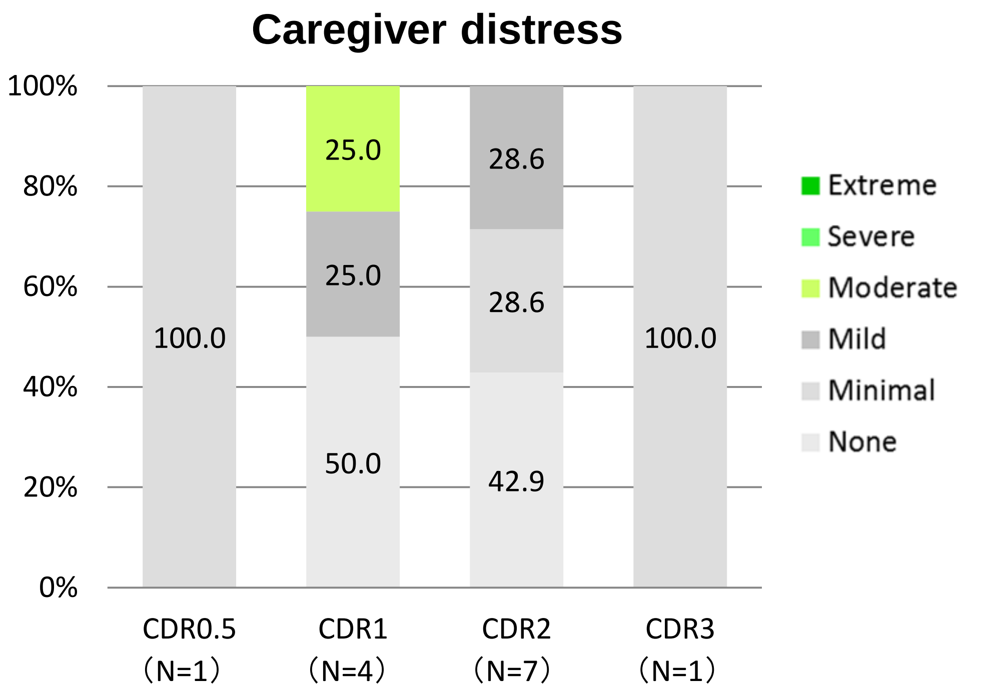
Caregiver distress
100%
80%
60%
40%
20%
0%
100.0
25.0
25.0
50.0
28.6
28.6
42.9
100.0
CDR0.5 （N=1）
CDR1 （N=4）
CDR2 （N=7）
CDR3 （N=1）
Extreme
Severe
Moderate
Mild
Minimal
None

Apathy

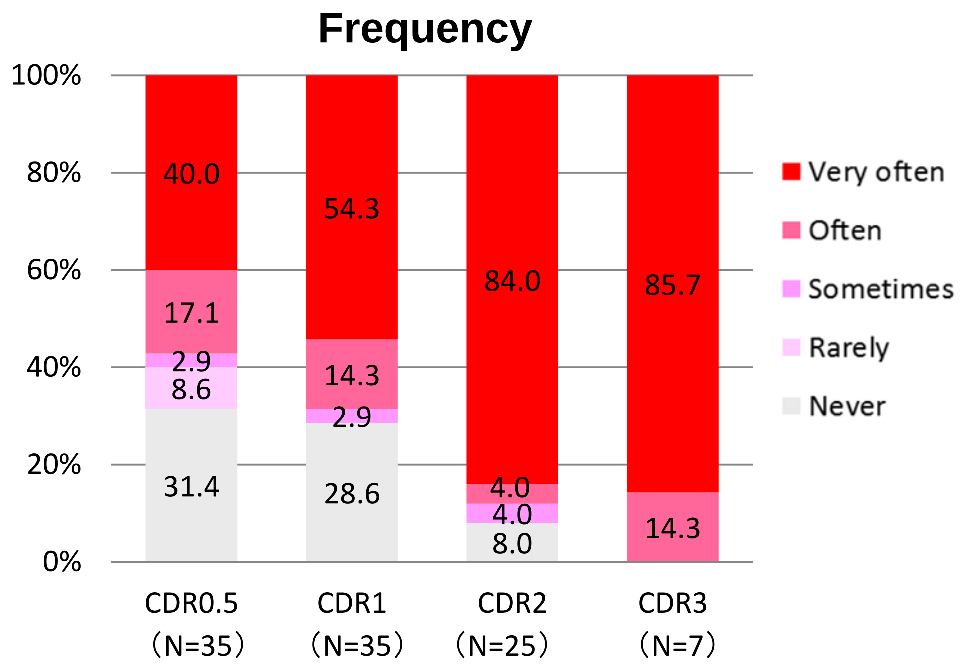
Frequency
100%
80%
60%
40%
20%
0%
40.0
17.1
2.9
8.6
31.4
54.3
14.3
2.9
28.6
84.0
4.0
4.0
8.0
85.7
14.3
CDR0.5 （N=35）
CDR1 （N=35）
CDR2 （N=25）
CDR3 （N=7）
Very often
Often
Sometimes
Rarely
Never

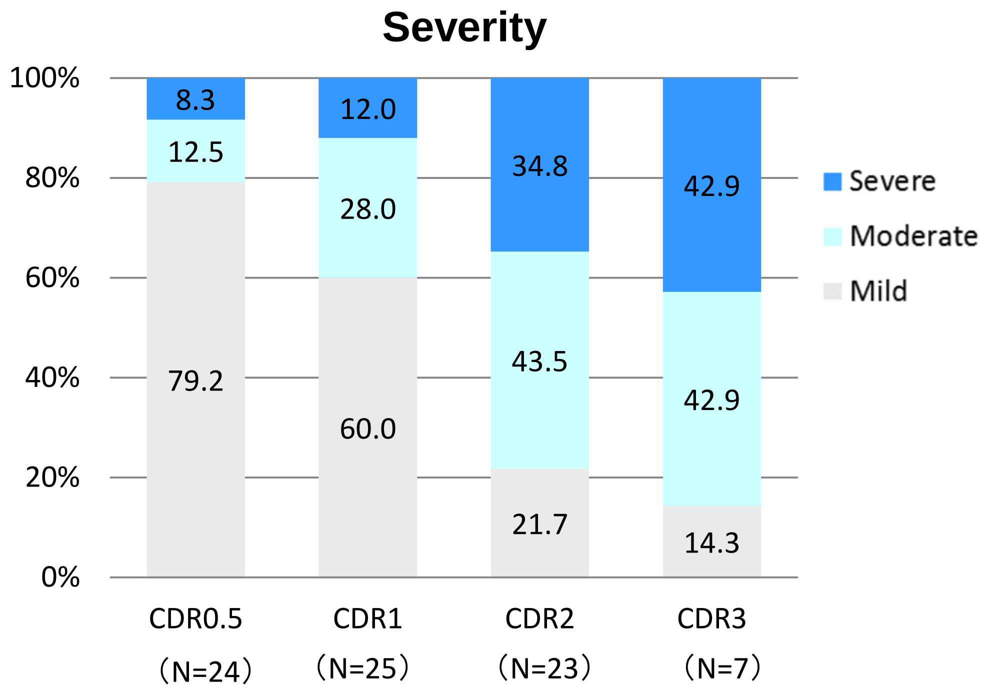
Severity
100%
80%
60%
40%
20%
0%
8.3
12.5
79.2
12.0
28.0
60.0
34.8
43.5
21.7
42.9
42.9
14.3
CDR0.5 （N=24）
CDR1 （N=25）
CDR2 （N=23）
CDR3 （N=7）
Severe
Moderate
Mild

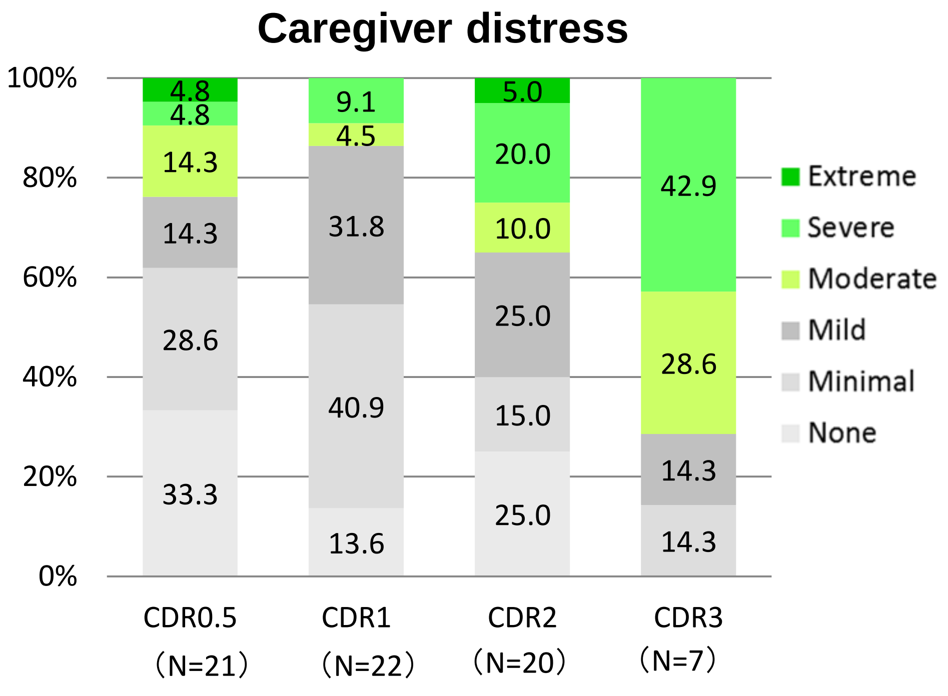
Caregiver distress
100%
80%
60%
40%
20%
0%
4.8
4.8
14.3
14.3
28.6
33.3
9.1
4.5
31.8
40.9
13.6
5.0
20.0
10.0
25.0
15.0
25.0
42.9
28.6
14.3
14.3
CDR0.5 （N=21）
CDR1 （N=22）
CDR2 （N=20）
CDR3 （N=7）
Extreme
Severe
Moderate
Mild
Minimal
None

Disinhibition

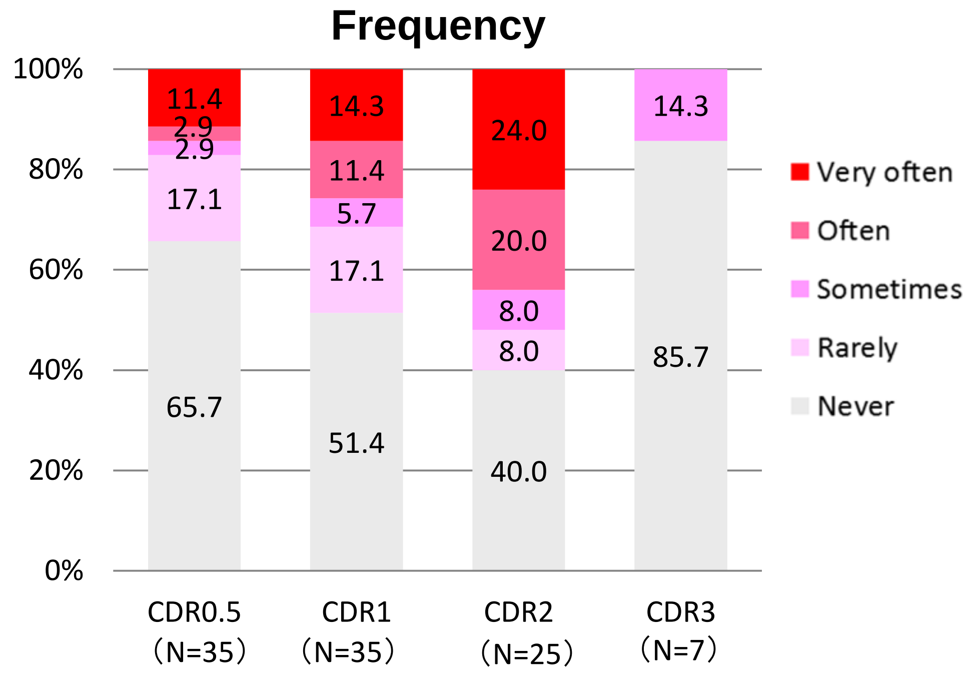

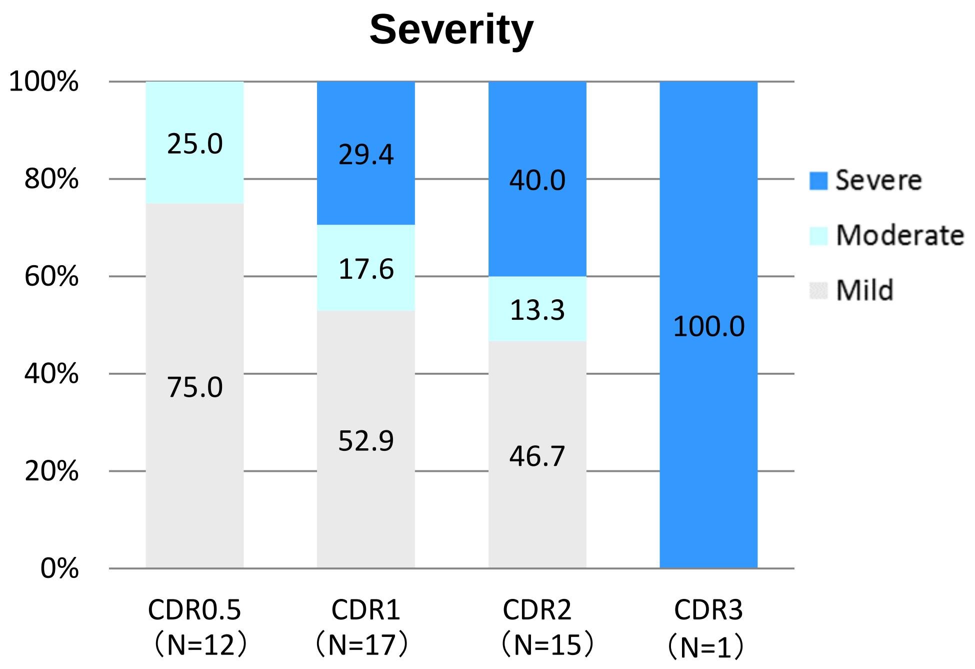

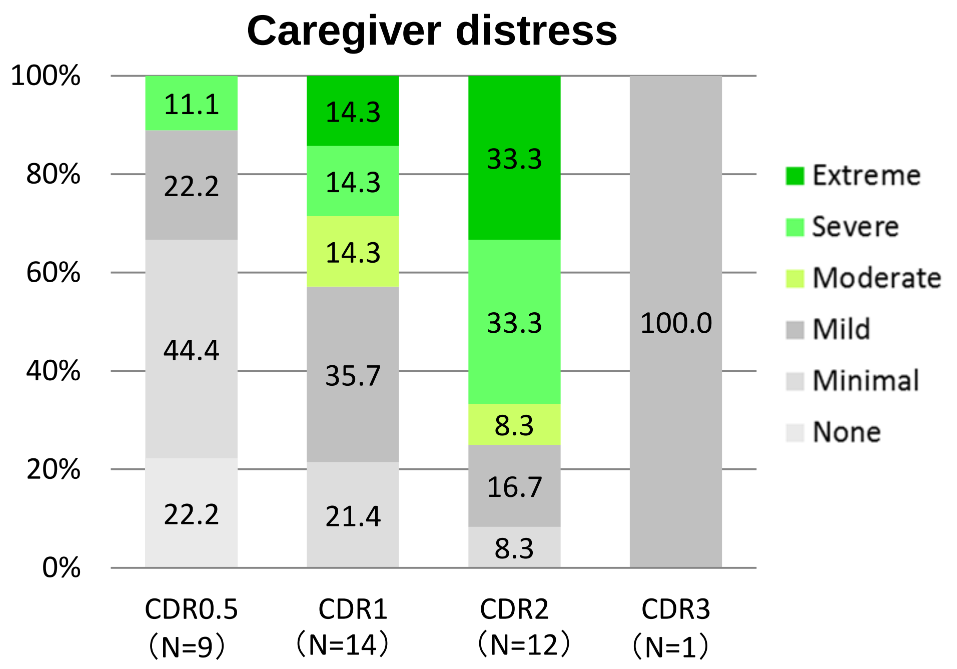

Irritability

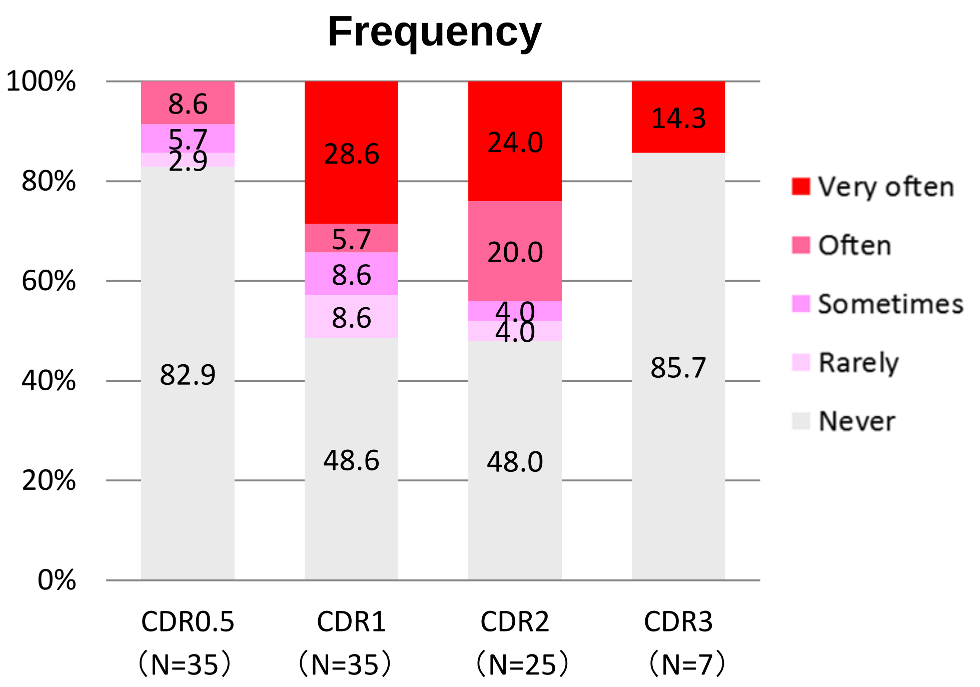

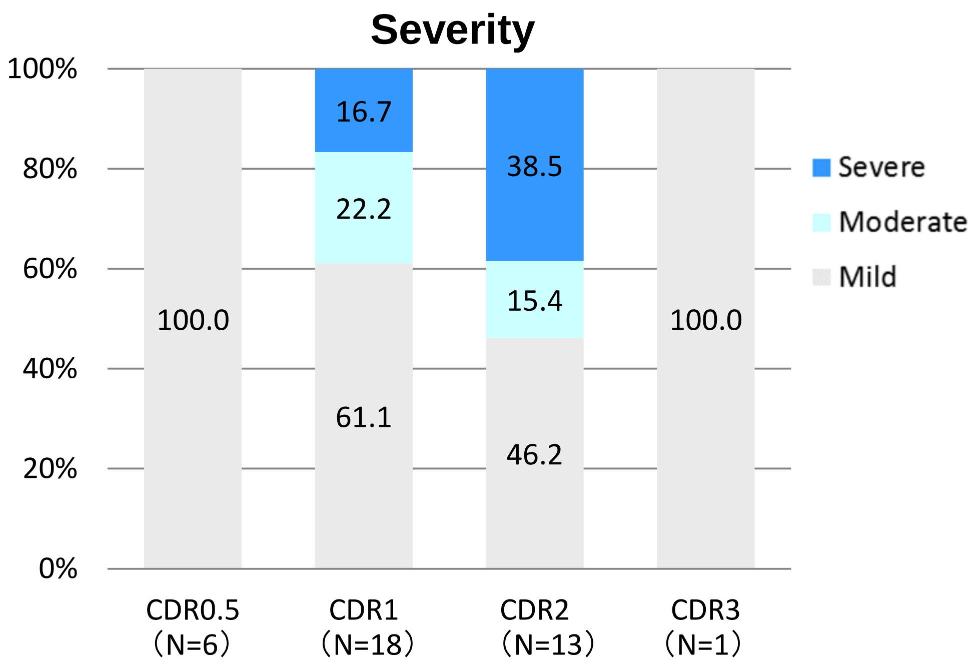

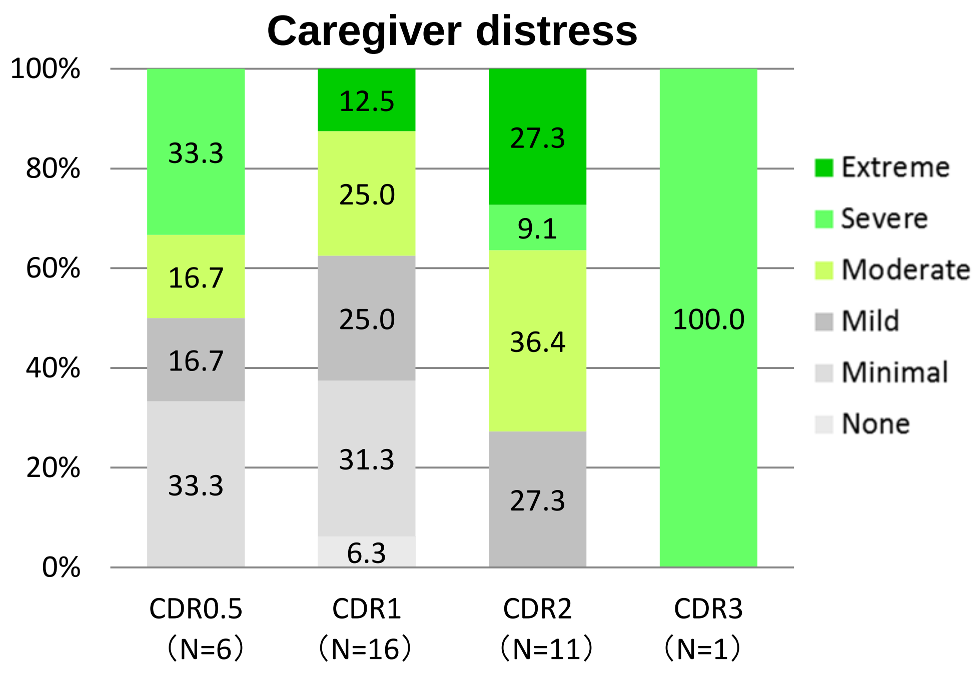

Aberrant motor behavior

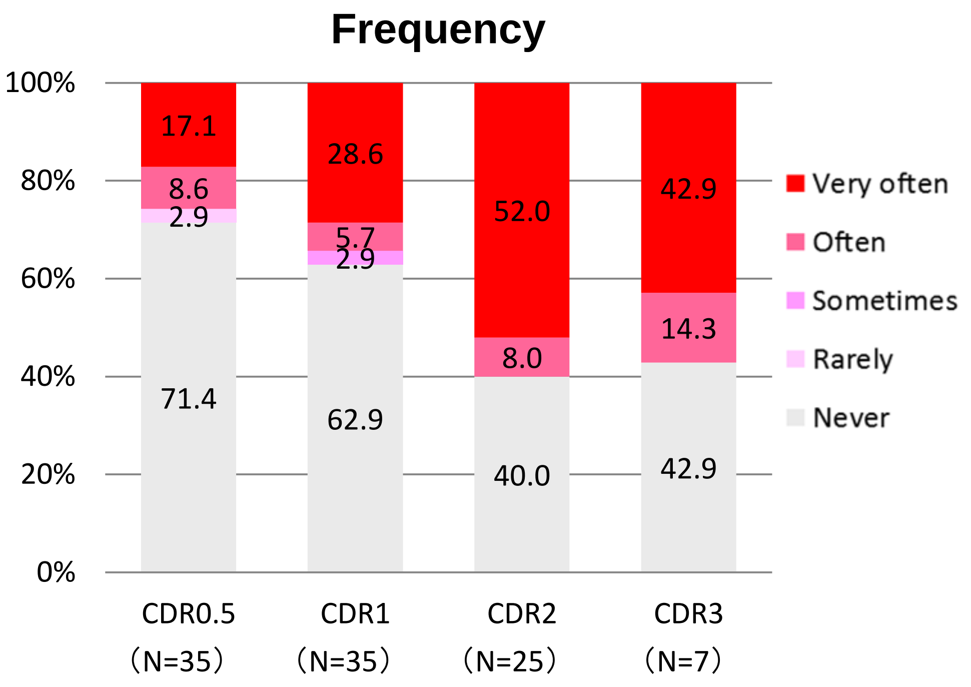

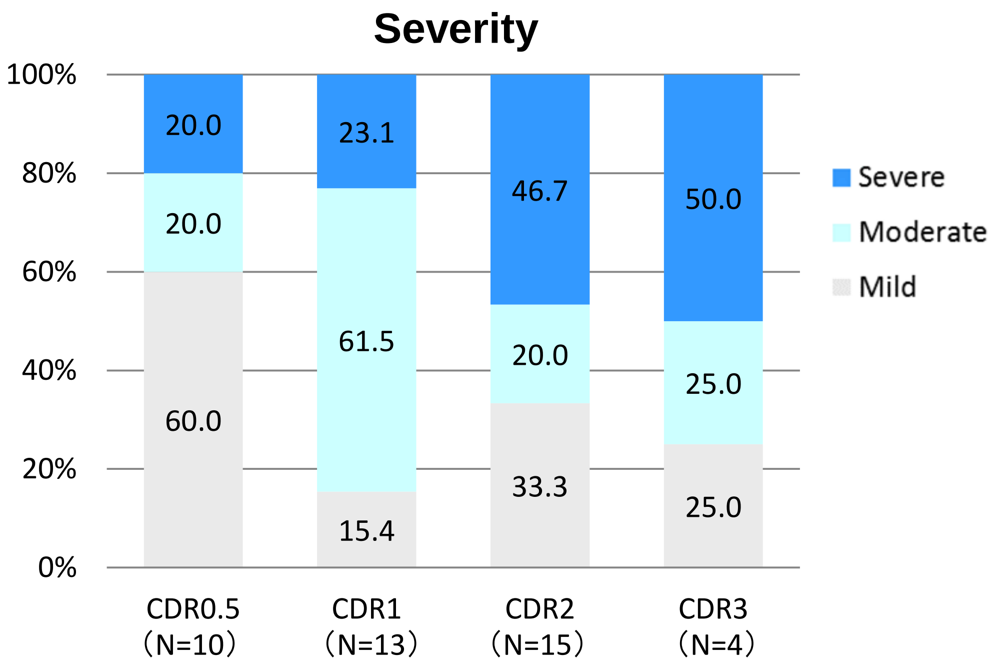

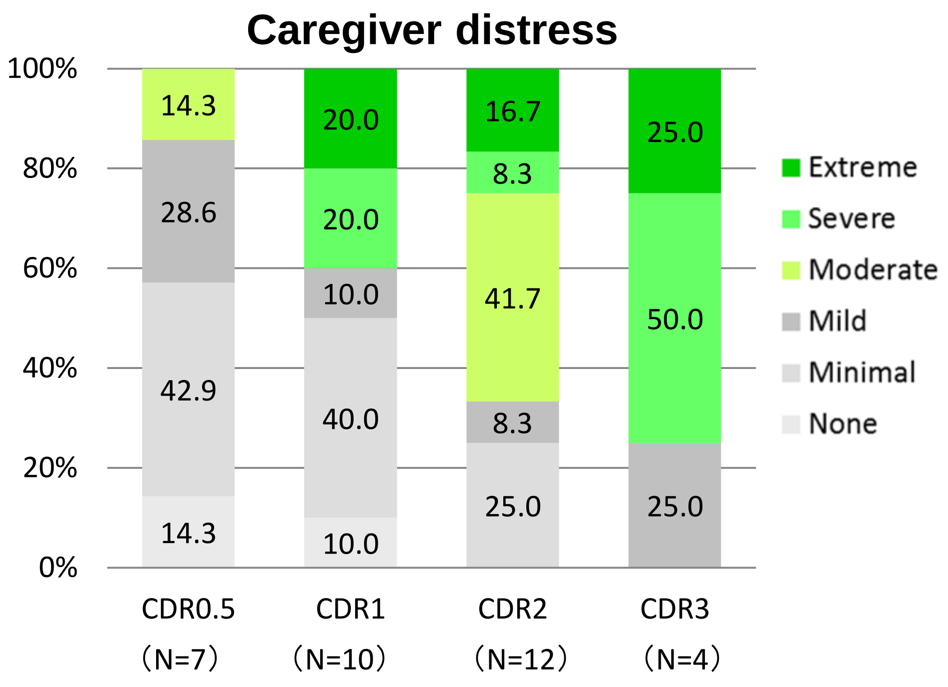

Sleep disturbances

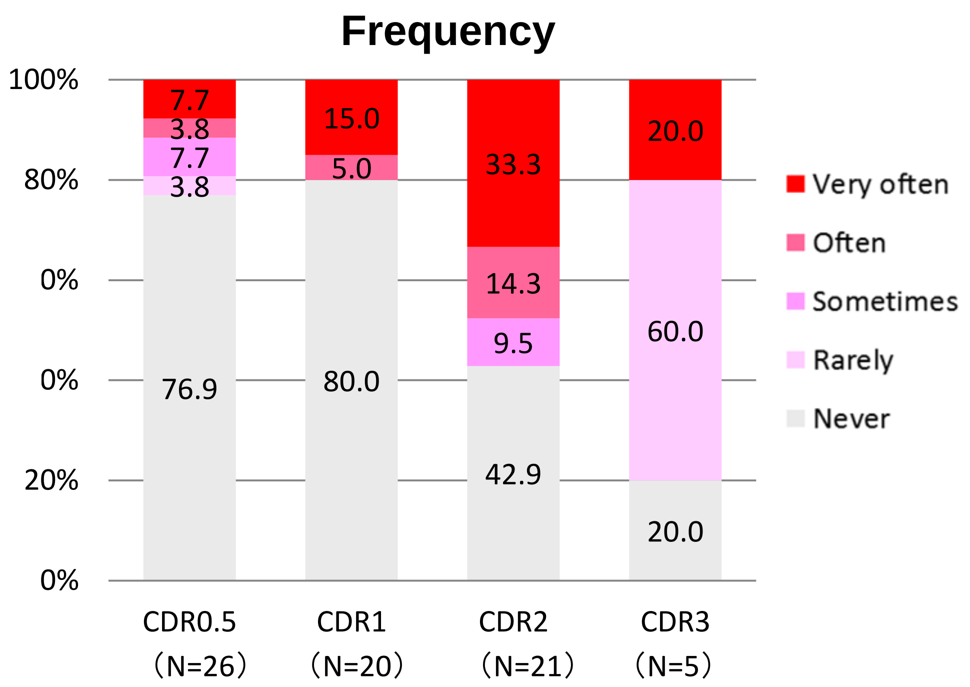

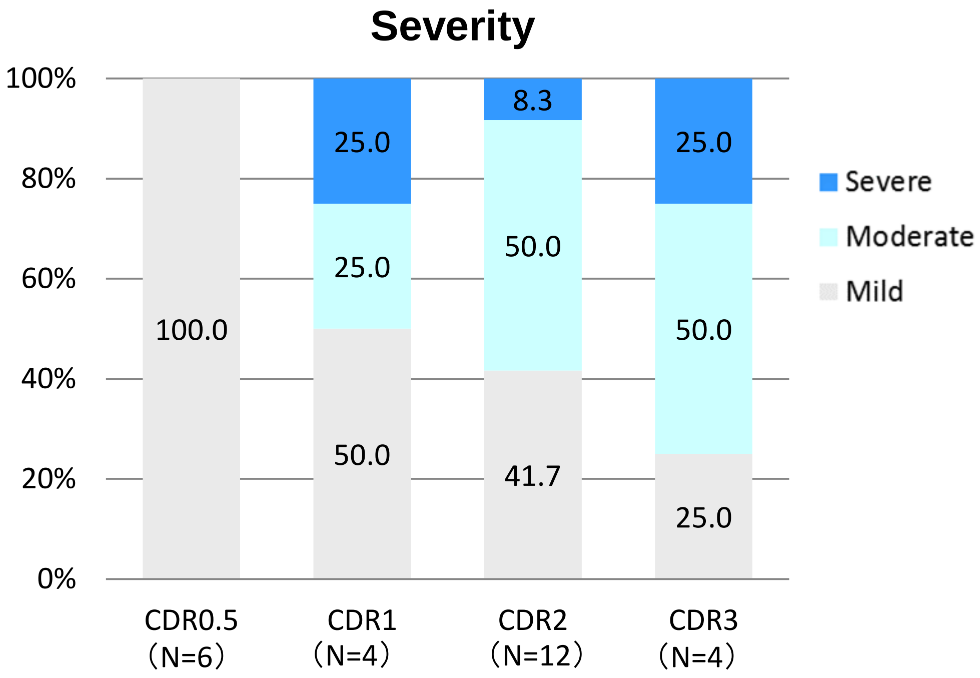

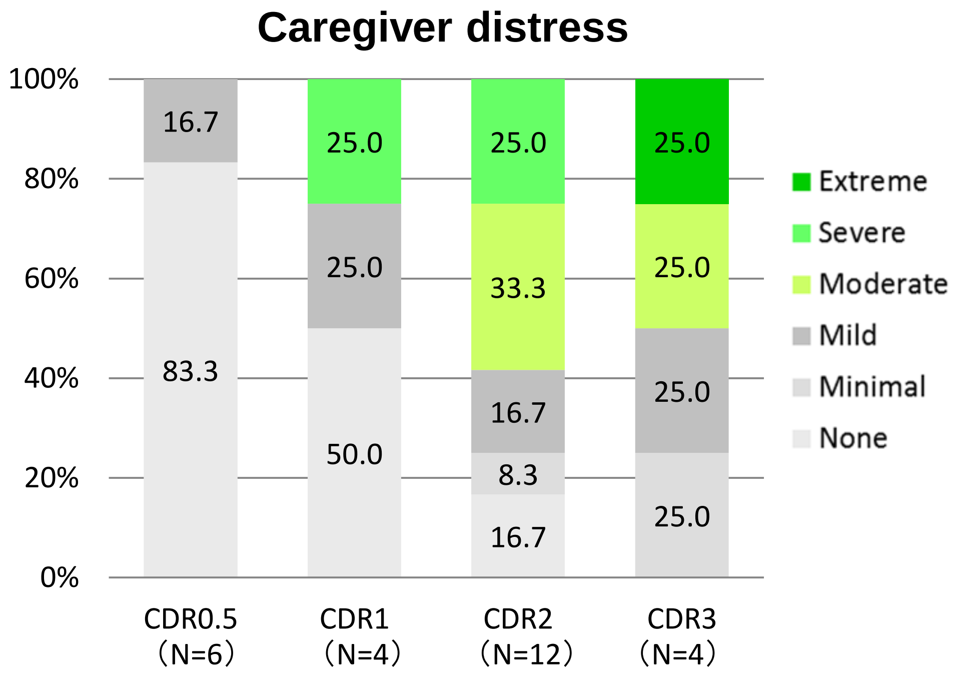

Eating abnormalities

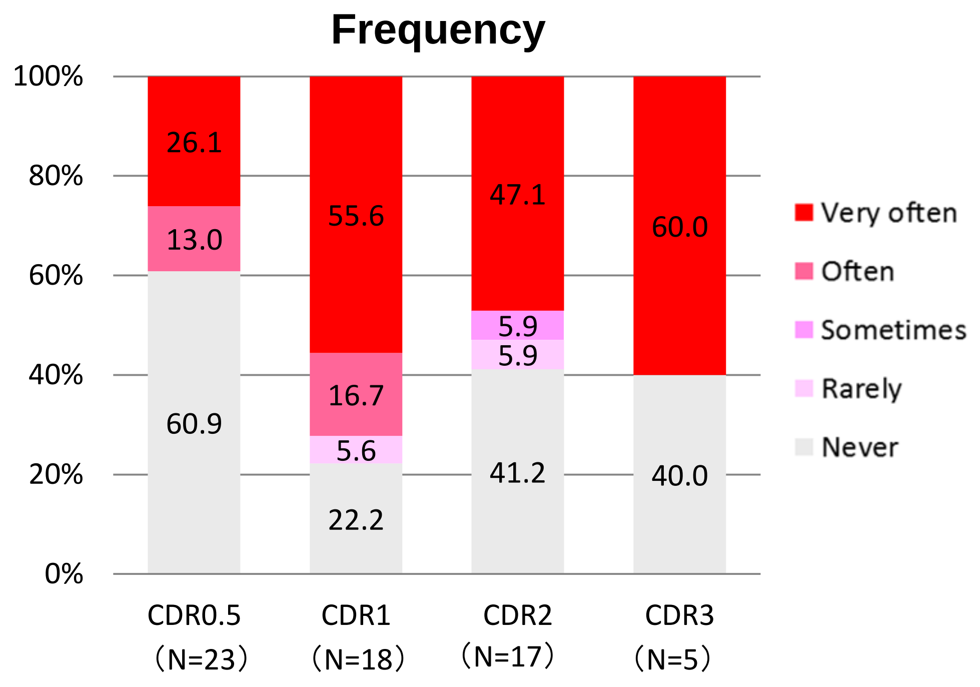

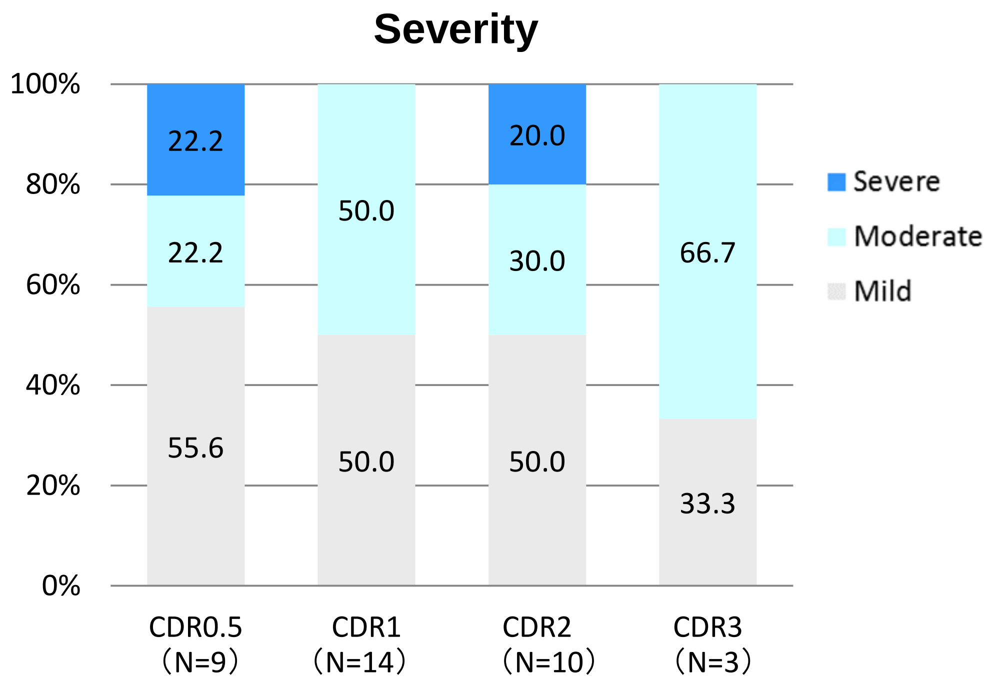

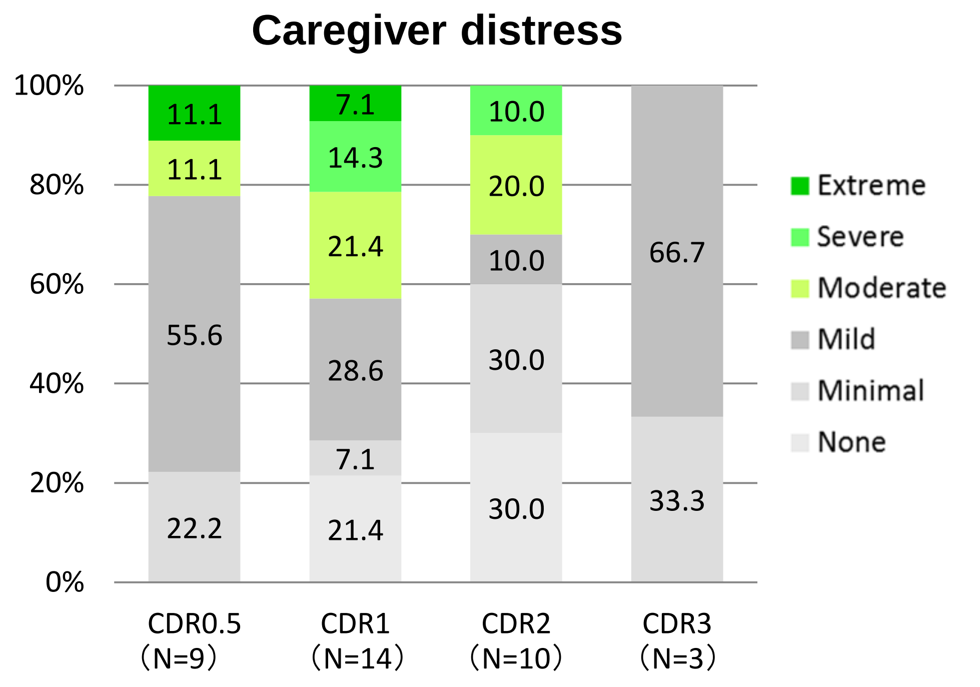

Supplement: S4 File — (PDF) [file pone.0161092.s004.pdf]
