# Supplementary material for: Differences of Behavioral and Psychological Symptoms of Dementia in Disease Severity in Four Major Dementias
Source: PLoS One. 2016 Aug 18;11(8):e0161092. doi: 10.1371/journal.pone.0161092 (PMC4990196; doi:10.1371/journal.pone.0161092)
Supplement: S1 Table — (DOCX) [file pone.0161092.s005.docx]

**S1 Table. Demographic data of patients with four dementias in this study**

|  | CDR | | | |  | Post hoc test^2^ | | |
| --- | --- | --- | --- | --- | --- | --- | --- | --- |
| Patient data | 0.5 | 1 | 2 | 3 | p value^1^ | 0.5 vs 1, 2, 3 | 1 vs 2, 3 | 2 vs 3 |
| Alzheimer’s disease |  |  |  |  |  |  |  |  |
| No. patients (male) | 371(131) | 479(149) | 195(50) | 46(9) |  |  |  |  |
| Age, years | 75.2±9.4 | 77.2±8.0 | 78.3±8.6 | 81.3±8.3 | <0.001 | 0.5<1, 2 | 1<3 |  |
| Education, years | 11.3±2.7 | 10.9±2.9 | 10.0±2.7 | 10.6±3.1 | <0.001 | 0.5>2 | 1>2 |  |
| MMSE score | 22.0±3.2 | 19.3±4.3 | 14.4±5.0 | 8.4±5.8 | <0.001 | 0.5>1, 2, 3 | 1>2, 3 | 2>3 |
| Dementia with Lewy bodies |  |  |  |  |  |  |  |  |
| No. patients (male) | 64(27) | 109(43) | 62(26) | 14(6) |  |  |  |  |
| Age, years | 79.2±5.2 | 78.0±6.1 | 79.9±6.2 | 80.4±5.3 | 0.266 |  |  |  |
| Education, years | 10.5±2.6 | 10.5±2.8 | 10.6±2.8 | 10.2±2.0 | 0.972 |  |  |  |
| MMSE score | 22.1±4.1 | 19.5±4.3 | 16.1±5.1 | 11.3±7.1 | <0.001 | 0.5>1, 2, 3 | 1>2, 3 |  |
| Vascular dementia |  |  |  |  |  |  |  |  |
| No. patients (male) | 40(17) | 73(40) | 31(19) | 12(4) |  |  |  |  |
| Age, years | 72.0±10.5 | 75.9±8.8 | 79.4±10.0 | 79.3±11.4 | 0.003 | 0.5<2 |  |  |
| Education, years | 11.3±2.6 | 11.0±2.7 | 10.0±2.8 | 9.3±0.9 | 0.076 |  |  |  |
| MMSE score | 22.9±3.4 | 20.5±3.5 | 16.1±4.7 | 11.5±5.4 | <0.001 | 0.5>1, 2, 3 | 1>2, 3 |  |
| Frontotemporal lobar degeneration |  |  |  |  |  |  |  |  |
| No. patients (male) | 35(15) | 35(18) | 25(14) | 7(5) |  |  |  |  |
| Age, years | 68.2±7.6 | 70.8±8.8 | 71.8±8.6 | 67.3±8.2 | 0.292 |  |  |  |
| Education, years | 12±2.6 | 11.1±2.6 | 12.4±3.5 | 10.8±1.8 | 0.387 |  |  |  |
| MMSE score | 22.5±5.2 | 17.8±6.2 | 15.3±5.8 | 7.2±4.3 | <0.001 | 0.5>1, 2, 3 | 1>3 | 2>3 |

CDR: clinical dementia rating, MMSE: Mini Mental State Examination

^1^Comparison between 4 CDR groups, Kruskal-Wallis test

^2^Mann-Whitney U test (p <0.05/6=0.0083)
