# Supplementary material for: Differences of Behavioral and Psychological Symptoms of Dementia in Disease Severity in Four Major Dementias
Source: PLoS One. 2016 Aug 18;11(8):e0161092. doi: 10.1371/journal.pone.0161092 (PMC4990196; doi:10.1371/journal.pone.0161092)
Supplement: S2 Table — (DOCX) [file pone.0161092.s006.docx]

**S2** **Table. Factor loadings for BPSDs in patients with Alzheimer’s disease**

|  | Factor 1 | Factor 2 | Factor 3 |
| --- | --- | --- | --- |
| Eigenvalues | 2.83 | 1.25 | 1.17 |
| % of variance explained | 23.6 | 10.4 | 9.7 |
| Delusions | **0.539** | 0.183 | 0.268 |
| Hallucinations | 0.057 | **0.352** | **0.397** |
| Agitation | **0.775** | 0.039 | 0.143 |
| Depression | **0.529** | 0.203 | -0.185 |
| Anxiety | **0.508** | 0.257 | -0.072 |
| Euphoria | -0.109 | 0.086 | **0.740** |
| Apathy | 0.153 | **0.629** | -0.198 |
| Disinhibition | **0.360** | -0.057 | **0.681** |
| Irritability | **0.723** | 0.036 | 0.127 |
| AMB | 0.166 | **0.559** | 0.219 |
| Sleep disturbances | 0.155 | **0.637** | 0.128 |
| Eating abnormalities | 0.042 | **0.514** | 0.043 |

AMB: Aberrant motor behavior

Significant loadings (≥ 0.30) were entered into the factor and are displayed in boldface.

The value of KMO was 0.766, and the Barlett’s sphericity test reached statistical significance (χ²=1060.1, df=66, p<0.001). The PCA found three components with eigenvalues exceeding 1, explaining 23.6, 10.4, and 9.7 percent of the variance respectively. Moreover, a plain break after the third component was seen by visual inspection of the scree plot. The Varimax rotation classified the 12 BPSDs into three factors.
