# Supplementary material for: Differences of Behavioral and Psychological Symptoms of Dementia in Disease Severity in Four Major Dementias
Source: PLoS One. 2016 Aug 18;11(8):e0161092. doi: 10.1371/journal.pone.0161092 (PMC4990196; doi:10.1371/journal.pone.0161092)
Supplement: S3 Table — (DOCX) [file pone.0161092.s007.docx]

**S3 Table. Neuropsychiatric Inventory scores of individual domains according to dementia severity in patients with Alzheimer’s disease**

|  |  | CDR | | | |  | Post hoc test^2^ | | |
| --- | --- | --- | --- | --- | --- | --- | --- | --- | --- |
| Symptoms |  | 0.5 | 1 | 2 | 3 | p value^1^ | 0.5 vs 1, 2, 3 | 1 vs 2, 3 | 2 vs 3 |
| Delusions | frequency | 0.5±1.1 | 0.7±1.2 | 1.1±1.6 | 1.4±1.8 | <0.001 | 0.5<2, 3 | 1<2, 3 |  |
|  | severity | 1.6±0.8 | 1.7±0.8 | 1.6±0.7 | 1.5±0.7 | 0.640 |  |  |  |
|  | ACD | 2.1±1.2 | 2.3±1.3 | 2.4±1.5 | 2.6±0.9 | 0.357 |  |  |  |
| Hallucinations | frequency | 0.1±0.5 | 0.2±0.7 | 0.4±1.0 | 0.9±1.4 | <0.001 | 0.5<2, 3 | 1<2, 3 | 2<3 |
|  | severity | 1.1±0.3 | 1.4±0.7 | 1.4±0.7 | 1.5±0.7 | 0.369 |  |  |  |
|  | ACD | 1.4±1.2 | 1.5±1.3 | 2.3±1.5 | 1.9±1.3 | 0.126 |  |  |  |
| Agitation | frequency | 0.4±1.0 | 0.8±1.3 | 1.3±1.6 | 1.8±1.8 | <0.001 | 0.5<1, 2, 3 | 1<2, 3 |  |
|  | severity | 1.4±0.6 | 1.5±0.6 | 1.7±0.7 | 1.8±0.7 | 0.017 |  |  |  |
|  | ACD | 2.0±1.3 | 2.3±1.3 | 2.7±1.3 | 2.8±1.3 | 0.001 | 0.5<2, 3 |  |  |
| Depression | frequency | 0.6±1.1 | 0.8±1.3 | 0.9±1.3 | 1.4±1.6 | <0.001 | 0.5<2, 3 |  |  |
|  | severity | 1.2±0.5 | 1.3±0.5 | 1.3±0.5 | 1.4±0.7 | 0.386 |  |  |  |
|  | ACD | 1.5±1.0 | 1.8±1.2 | 2.0±1.2 | 2.2±0.8 | 0.009 | 0.5<2, 3 |  |  |
| Anxiety | frequency | 0.6±1.2 | 0.7±1.4 | 1.1±1.6 | 1.1±1.6 | 0.001 | 0.5<2 | 1<2 |  |
|  | severity | 1.3±0.5 | 1.4±0.6 | 1.4±0.6 | 1.4±0.7 | 0.324 |  |  |  |
|  | ACD | 1.6±1.2 | 2.0±1.2 | 2.1±1.4 | 2.5±1.2 | 0.015 | 0.5<3 |  |  |
| Euphoria | frequency | 0.0±0.4 | 0.0±0.4 | 0.1±0.6 | 0.1±0.6 | 0.327 |  |  |  |
|  | severity | 1.2±0.4 | 1.2±0.6 | 1.5±0.5 | 3.0 | 0.064 |  |  |  |
|  | ACD | 1.5±1.0 | 0.9±1.1 | 1.9±1.7 | 0.0 | 0.303 |  |  |  |
| Apathy | frequency | 1.9±1.7 | 2.7±1.7 | 3.1±1.5 | 3.6±1.2 | <0.001 | 0.5<1, 2, 3 | 1<2, 3 |  |
|  | severity | 1.3±0.5 | 1.5±0.6 | 1.7±0.7 | 2.0±0.9 | <0.001 | 0.5<1, 2, 3 | 1<2, 3 |  |
|  | ACD | 1.1±0.9 | 1.4±1.1 | 1.9±1.2 | 2.1±1.3 | <0.001 | 0.5<2, 3 | 1<2, 3 |  |
| Disinhibition | frequency | 0.2±0.6 | 0.3±0.9 | 0.6±1.3 | 0.6±1.3 | <0.001 | 0.5<1, 2, 3 | 1<2 |  |
|  | severity | 1.6±0.6 | 1.5±0.6 | 1.8±0.7 | 1.4±0.7 | 0.103 |  |  |  |
|  | ACD | 2.3±1.4 | 2.3±1.5 | 2.8±1.5 | 2.4±1.5 | 0.238 |  |  |  |
| Irritability | frequency | 0.4±1.0 | 0.7±1.3 | 0.9±1.5 | 0.9±1.4 | <0.001 | 0.5<1, 2 |  |  |
|  | severity | 1.3±0.6 | 1.5±0.7 | 1.6±0.8 | 1.6±0.8 | 0.032 | 0.5<2 |  |  |
|  | ACD | 1.8±1.2 | 2.0±1.4 | 2.3±1.3 | 2.2±1.5 | 0.125 |  |  |  |
| AMB | frequency | 0.2±0.9 | 0.7±1.4 | 1.2±1.7 | 1.4±1.8 | <0.001 | 0.5<1, 2, 3 | 1<2, 3 |  |
|  | severity | 1.5±0.6 | 1.7±0.8 | 1.7±0.7 | 1.9±0.8 | 0.187 |  |  |  |
|  | ACD | 1.7±1.2 | 1.6±1.3 | 2.2±1.3 | 2.5±1.7 | 0.007 |  | 1<2 |  |
| Sleep disturbances | frequency | 0.4±1.1 | 0.8±1.5 | 1.1±1.6 | 1.5±1.7 | <0.001 | 0.5<1, 2, 3 | 1<3 |  |
|  | severity | 1.3±0.6 | 1.5±0.6 | 1.6±0.7 | 2.2±0.7 | <0.001 | 0.5<3 | 1<3 | 2<3 |
|  | ACD | 1.3±1.4 | 1.3±1.4 | 2.0±1.5 | 3.1±1.2 | <0.001 | 0.5<3 | 1<3 | 2<3 |
| Eating abnormalities | frequency | 0.5±1.2 | 0.9±1.5 | 1.0±1.6 | 0.9±1.7 | 0.001 | 0.5<1, 2 |  |  |
|  | severity | 1.6±0.6 | 1.6±0.6 | 1.6±0.6 | 1.7±0.8 | 0.952 |  |  |  |
|  | ACD | 0.9±1.1 | 1.3±1.2 | 1.7±1.3 | 2.4±1.3 | 0.004 | 0.5<2, 3 |  |  |

CDR: clinical dementia rating, ACD: associated caregiver distress, AMB: Aberrant motor behavior

^1^Comparison between 4 CDR groups, Kruskal-Wallis test

^2^Mann-Whitney U test (p <0.05/6=0.0083)
