# Supplementary material for: Differences of Behavioral and Psychological Symptoms of Dementia in Disease Severity in Four Major Dementias
Source: PLoS One. 2016 Aug 18;11(8):e0161092. doi: 10.1371/journal.pone.0161092 (PMC4990196; doi:10.1371/journal.pone.0161092)
Supplement: S4 Table — (DOCX) [file pone.0161092.s008.docx]

**S4 Table. Percentages of patients of individual domains according to dementia severity in patients with Alzheimer’s disease**

|  |  | CDR | | | |
| --- | --- | --- | --- | --- | --- |
| Symptoms | patients with | 0.5 | 1 | 2 | 3 |
| Delusions | symptom | 20.9 | 27.3 | 40.7 | 44.4 |
|  | severity 2 or 3 | 44.2 | 48.1 | 49.4 | 35.0 |
|  | ACD^1^ | 37.0 | 43.5 | 48.7 | 45.0 |
| Hallucinations | symptom | 4.6 | 7.0 | 14.5 | 35.5 |
|  | severity 2 or 3 | 11.8 | 24.2 | 28.6 | 37.5 |
|  | ACD^1^ | 23.5 | 24.2 | 37 | 37.5 |
| Agitation | symptom | 18.7 | 35.2 | 44.6 | 54.3 |
|  | severity 2 or 3 | 36.2 | 40.1 | 52.3 | 64.0 |
|  | ACD^1^ | 34.3 | 44.1 | 56.6 | 58.3 |
| Depression | symptom | 25.8 | 32.9 | 38.1 | 46.7 |
|  | severity 2 or 3 | 17.9 | 26.9 | 21.6 | 28.5 |
|  | ACD^1^ | 17.8 | 28.3 | 31.5 | 28.6 |
| Anxiety | symptom | 24.9 | 26.9 | 37.6 | 36.9 |
|  | severity 2 or 3 | 22.8 | 34.4 | 31.5 | 29.4 |
|  | ACD^1^ | 18.1 | 32.0 | 37.1 | 50.0 |
| Euphoria | symptom | 1.5 | 2.3 | 4.2 | 2.2 |
|  | severity 2 or 3 | 16.7 | 9.1 | 50.0 | 100 |
|  | ACD^1^ | 16.7 | 18.2 | 37.5 | 0 |
| Apathy | symptom | 61.2 | 77.8 | 83.5 | 91.1 |
|  | severity 2 or 3 | 25.1 | 42.2 | 56.8 | 61.0 |
|  | ACD^1^ | 7.2 | 16.0 | 35.0 | 30.7 |
| Disinhibition | symptom | 7.0 | 13.7 | 22.9 | 21.7 |
|  | severity 2 or 3 | 50.0 | 41.5 | 63.7 | 30.0 |
|  | ACD^1^ | 50.0 | 43.8 | 58.1 | 40.0 |
| Irritability | symptom | 20.3 | 29.4 | 31.6 | 32.6 |
|  | severity 2 or 3 | 24.0 | 38.8 | 47.5 | 40.0 |
|  | ACD^1^ | 25.0 | 32.3 | 46.8 | 40.0 |
| AMB | patients with symptom | 8.4 | 20.7 | 36.9 | 39.1 |
|  | severity 2 or 3 | 38.7 | 48.0 | 57.0 | 66.7 |
|  | ACD^1^ | 24.2 | 19.4 | 41.4 | 56.2 |
| Sleep disturbances | patients with symptom | 11.9 | 23.5 | 34.8 | 48.6 |
|  | severity 2 or 3 | 24.3 | 40.4 | 46.3 | 82.4 |
|  | ACD^1^ | 21.2 | 21.4 | 43.4 | 76.5 |
| Eating abnormalities | symptom | 13.8 | 26.0 | 28.0 | 23.3 |
|  | severity 2 or 3 | 50.0 | 50.0 | 47.5 | 57.1 |
|  | ACD^1^ | 11.4 | 14.7 | 25.0 | 57.1 |

CDR: clinical dementia rating, ACD: associated caregiver distress, ^1^Moderate or greater distress, AMB: Aberrant motor behavior
