# Supplementary material for: Differences of Behavioral and Psychological Symptoms of Dementia in Disease Severity in Four Major Dementias
Source: PLoS One. 2016 Aug 18;11(8):e0161092. doi: 10.1371/journal.pone.0161092 (PMC4990196; doi:10.1371/journal.pone.0161092)
Supplement: S5 Table — (DOCX) [file pone.0161092.s009.docx]

**S5** **Table. Factor loadings for BPSDs in patients with Dementia with Lewy bodies**

|  | Factor 1 | Factor 2 | Factor 3 | Factor 4 |
| --- | --- | --- | --- | --- |
| Eigenvalues | 3.25 | 1.48 | 1.23 | 1.11 |
| % of variance explained | 27.0 | 12.3 | 10.3 | 9.2 |
| Delusions | **0.831** | 0.246 | 0.130 | 0.057 |
| Hallucinations | **0.818** | 0.051 | 0.163 | -0.119 |
| Agitation | **0.622** | -0.137 | 0.046 | **0.514** |
| Depression | -0.024 | **0.828** | 0.152 | -0.024 |
| Anxiety | 0.073 | **0.770** | 0.056 | 0.173 |
| Euphoria | -0.273 | 0.108 | -0.180 | **0.679** |
| Apathy | 0.054 | 0.071 | **0.455** | **0.429** |
| Disinhibition | 0.077 | 0.086 | 0.281 | **0.480** |
| Irritability | **0.352** | 0.243 | 0.110 | **0.588** |
| AMB | 0.049 | 0.135 | **0.791** | -0.071 |
| Sleep disturbances | 0.243 | 0.077 | **0.760** | 0.159 |
| Eating abnormalities | **0.327** | **0.495** | 0.072 | 0.211 |

AMB: Aberrant motor behavior

Significant loadings (≥ 0.30) were entered into the factor and are displayed in boldface.

The value of KMO was 0.673, and the Barlett’s sphericity test reached statistical significance (χ²=422.0, df=66, p<0.001). The PCA found four components with eigenvalues exceeding 1, explaining 27.0, 12.3, 10.3, and 9.2 percent of the variance respectively. Moreover, a plain break after the fourth component was seen by visual inspection of the scree plot. The Varimax rotation classified the 12 BPSD into four factors.
