# Supplementary material for: Differences of Behavioral and Psychological Symptoms of Dementia in Disease Severity in Four Major Dementias
Source: PLoS One. 2016 Aug 18;11(8):e0161092. doi: 10.1371/journal.pone.0161092 (PMC4990196; doi:10.1371/journal.pone.0161092)
Supplement: S7 Table — (DOCX) [file pone.0161092.s011.docx]

**S7 Table. Percentages of patients of individual domains according to dementia severity in patients with Dementia with Lewy bodies**

|  |  | CDR | | | |
| --- | --- | --- | --- | --- | --- |
| Symptoms | patients with | 0.5 | 1 | 2 | 3 |
| Delusions | symptom | 50.8 | 60.5 | 74.2 | 71.4 |
|  | severity 2 or 3 | 65.6 | 54.5 | 56.5 | 50.0 |
|  | ACD^1^ | 48.2 | 53.4 | 50.0 | 40.0 |
| Hallucinations | symptom | 66.7 | 63.3 | 69.4 | 64.3 |
|  | severity 2 or 3 | 57.2 | 52.2 | 44.2 | 33.3 |
|  | ACD^1^ | 38.9 | 46.8 | 37.5 | 37.5 |
| Agitation | symptom | 30.7 | 30.3 | 48.4 | 42.8 |
|  | severity 2 or 3 | 26.3 | 33.4 | 46.6 | 50.0 |
|  | ACD^1^ | 33.4 | 53.6 | 53.9 | 66.7 |
| Depression | symptom | 33.3 | 36.7 | 37.1 | 35.7 |
|  | severity 2 or 3 | 52.4 | 27.5 | 30.4 | 0.0 |
|  | ACD^1^ | 27.8 | 36.2 | 40.9 | 40 |
| Anxiety | symptom | 33.9 | 36.7 | 48.4 | 28.6 |
|  | severity 2 or 3 | 42.9 | 40.0 | 13.3 | 25 |
|  | ACD^1^ | 55.6 | 37.1 | 32.0 | 50.0 |
| Euphoria | symptom | 3.2 | 2.7 | 4.8 | 0.0 |
|  | severity 2 or 3 | 50 | 33.3 | 66.7 | - |
|  | ACD^1^ | 0.0 | 0.0 | 0.0 | - |
| Apathy | symptom | 62.5 | 79.8 | 82.3 | 100 |
|  | severity 2 or 3 | 42.5 | 48.3 | 80.4 | 64.3 |
|  | ACD^1^ | 20.6 | 25.3 | 39.1 | 46.1 |
| Disinhibition | symptom | 12.9 | 9.2 | 16.1 | 14.3 |
|  | severity 2 or 3 | 50.0 | 40.0 | 80.0 | 50.0 |
|  | ACD^1^ | 37.5 | 55.5 | 90.0 | 50.0 |
| Irritability | symptom | 31.7 | 27.4 | 45.2 | 28.6 |
|  | severity 2 or 3 | 25.0 | 53.3 | 46.4 | 0.0 |
|  | ACD^1^ | 29.4 | 42.8 | 56.0 | 50.0 |
| AMB | symptom | 14.6 | 26.6 | 37.1 | 57.1 |
|  | severity 2 or 3 | 44.4 | 51.7 | 56.5 | 62.5 |
|  | ACD^1^ | 44.4 | 30.8 | 44.4 | 28.6 |
| Sleep disturbances | symptom | 48.9 | 54.0 | 68.1 | 81.8 |
|  | severity 2 or 3 | 40.9 | 41.5 | 56.3 | 77.8 |
|  | ACD^1^ | 36.4 | 26.8 | 50.0 | 50.0 |
| Eating abnormalities | symptom | 23.2 | 31.9 | 26.8 | 11.1 |
|  | severity 2 or 3 | 80.0 | 54.5 | 54.6 | 0.0 |
|  | ACD^1^ | 40.0 | 23.8 | 0.0 | 0.0 |

CDR: clinical dementia rating, ACD: associated caregiver distress, ^1^Moderate or greater distress, AMB: Aberrant motor behavior
