# Supplementary material for: Differences of Behavioral and Psychological Symptoms of Dementia in Disease Severity in Four Major Dementias
Source: PLoS One. 2016 Aug 18;11(8):e0161092. doi: 10.1371/journal.pone.0161092 (PMC4990196; doi:10.1371/journal.pone.0161092)
Supplement: S8 Table — (DOCX) [file pone.0161092.s012.docx]

**S8 Table. Factor loadings for BPSDs in patients with Vascular dementia**

|  | Factor 1 | Factor 2 | Factor 3 | Factor 4 |
| --- | --- | --- | --- | --- |
| Eigenvalues | 3.26 | 1.86 | 1.40 | 1.18 |
| % of variance explained | 27.1 | 15.5 | 11.6 | 9.8 |
| Delusions | 0.260 | 0.142 | -0.019 | **0.806** |
| Hallucinations | -0.070 | -0.010 | 0.015 | **0.868** |
| Agitation | **0.868** | 0.212 | 0.016 | 0.006 |
| Depression | -0.192 | **0.781** | 0.039 | 0.091 |
| Anxiety | 0.132 | **0.783** | 0.128 | 0.055 |
| Euphoria | -0.126 | -0.248 | **0.783** | -0.004 |
| Apathy | 0.205 | **0.626** | -0.201 | -0.047 |
| Disinhibition | **0.728** | -0.201 | 0.296 | 0.268 |
| Irritability | **0.890** | 0.111 | 0.188 | 0.021 |
| AMB | **0.326** | 0.053 | **0.633** | 0.004 |
| Sleep disturbances | **0.337** | **0.409** | **0.364** | 0.145 |
| Eating abnormalities | 0.240 | 0.219 | **0.548** | -0.017 |

AMB: Aberrant motor behavior

Significant loadings (≥ 0.30) were entered into the factor and are displayed in boldface.

The value of KMO was 0.689, and the Barlett’s sphericity test reached statistical significance (χ²=335.4, df=66, p<0.001). The PCA found four components with eigenvalues exceeding 1, explaining 27.1, 15.5, 11.6, and 9.8 percent of the variance respectively. Moreover, a plain break after the fourth component was seen by visual inspection of the scree plot. The Varimax rotation classified the 12 BPSD into four factors.
