# Supplementary material for: Differences of Behavioral and Psychological Symptoms of Dementia in Disease Severity in Four Major Dementias
Source: PLoS One. 2016 Aug 18;11(8):e0161092. doi: 10.1371/journal.pone.0161092 (PMC4990196; doi:10.1371/journal.pone.0161092)
Supplement: S9 Table — (DOCX) [file pone.0161092.s013.docx]

**S9 Table. Neuropsychiatric Inventory scores of individual domains according to dementia severity in patients with Vascular dementia**

|  |  | CDR | | | |  | Post hoc test^2^ | | |
| --- | --- | --- | --- | --- | --- | --- | --- | --- | --- |
| Symptoms |  | 0.5 | 1 | 2 | 3 | p value^1^ | 0.5 vs 1, 2, 3 | 1 vs 2, 3 | 2 vs 3 |
| Delusions | frequency | 0.4±1.2 | 0.3±0.9 | 0.7±1.4 | 1.8±2.0 | 0.007 |  | 1<3 |  |
|  | severity | 2.0±0.9 | 1.9±0.9 | 2.1±0.8 | 2.0±0.6 | 0.948 |  |  |  |
|  | ACD | 3.6±1.3 | 2.4±1.6 | 3.4±1.6 | 3.5±1.3 | 0.424 |  |  |  |
| Hallucinations | frequency | 0.2±0.7 | 0.3±0.8 | 0.3±1.0 | 1.0±1.5 | 0.053 |  |  |  |
|  | severity | 1.4±0.5 | 1.6±0.9 | 2.3±1.0 | 1.8±1.1 | 0.516 |  |  |  |
|  | ACD | 1.5±0.6 | 1.9±1.5 | 3.0±2.2 | 2.8±2.1 | 0.570 |  |  |  |
| Agitation | frequency | 0.6±1.2 | 1.1±1.6 | 1.7±1.7 | 2.4±1.8 | 0.002 | 0.5<2, 3 |  |  |
|  | severity | 1.7±0.5 | 1.7±0.8 | 1.7±0.8 | 2.1±0.8 | 0.554 |  |  |  |
|  | ACD | 2.4±1.3 | 2.8±1.3 | 3.5±1.2 | 3.3±1.6 | 0.187 |  |  |  |
| Depression | frequency | 0.7±1.3 | 0.7±1.3 | 0.9±1.5 | 1.0±1.5 | 0.738 |  |  |  |
|  | severity | 1.6±0.9 | 1.5±0.7 | 1.5±0.7 | 1.4±0.9 | 0.933 |  |  |  |
|  | ACD | 1.5±1.0 | 2.1±1.1 | 3.0±1.4 | 2.4±1.3 | 0.059 |  |  |  |
| Anxiety | frequency | 0.7±1.3 | 0.6±1.3 | 0.9±1.6 | 1.0±1.5 | 0.887 |  |  |  |
|  | severity | 1.6±0.9 | 1.2±0.4 | 1.3±0.5 | 1.3±0.5 | 0.659 |  |  |  |
|  | ACD | 1.8±0.7 | 1.7±1.3 | 3.4±0.9 | 2.5±1.3 | 0.012 | 0.5<2 | 1<2 |  |
| Euphoria | frequency | 0.2±0.8 | 0.2±0.7 | 0.1±0.7 | 0.3±0.9 | 0.887 |  |  |  |
|  | severity | 1.3±0.6 | 1.2±0.4 | 2.0 | 2.0 | 0.321 |  |  |  |
|  | ACD | 1.3±1.5 | 0.8±0.8 | 3.0 | 3.0 | 0.274 |  |  |  |
| Apathy | frequency | 1.9±1.8 | 3.0±1.4 | 3.6±1.2 | 3.7±1.2 | <0.001 | 0.5<1, 2, 3 | 1<2 |  |
|  | severity | 1.3±0.5 | 1.7±0.7 | 1.9±0.7 | 2.1±0.7 | 0.010 | 0.5<2, 3 |  |  |
|  | ACD | 1.2±0.8 | 1.7±1.2 | 2.2±1.3 | 3.2±1.4 | 0.001 | 0.5<2, 3 | 1<3 |  |
| Disinhibition | frequency | 0.5±1.2 | 0.6±1.3 | 1.0±1.6 | 1.4±1.9 | 0.182 |  |  |  |
|  | severity | 1.6±0.8 | 1.6±0.8 | 1.8±0.6 | 2.2±0.4 | 0.251 |  |  |  |
|  | ACD | 2.6±1.7 | 2.8±1.3 | 2.6±1.2 | 3.0±1.4 | 0.901 |  |  |  |
| Irritability | frequency | 1.1±1.5 | 1.0±1.5 | 1.5±1.7 | 2.2±2.0 | 0.130 |  |  |  |
|  | severity | 1.3±0.6 | 1.6±0.8 | 2.1±0.8 | 2.0±1.0 | 0.025 | 0.5<2 |  |  |
|  | ACD | 1.7±1.2 | 2.3±1.5 | 3.0±2.0 | 3.4±1.5 | 0.087 |  |  |  |
| AMB | frequency | 0.3±0.9 | 0.4±1.1 | 0.6±1.4 | 1.5±1.9 | 0.046 | 0.5<3 |  |  |
|  | severity | 1.3±0.5 | 2.0±0.7 | 1.6±0.5 | 2.6±0.9 | 0.057 |  |  |  |
|  | ACD | 2.0±1.6 | 1.9±1.6 | 2.3±0.5 | 4.7±0.6 | 0.072 |  |  |  |
| Sleep disturbances | frequency | 0.8±1.5 | 1.0±1.6 | 2.3±1.9 | 2.9±1.7 | 0.001 | 0.5<2, 3 | 1<2, 3 |  |
|  | severity | 1.0±0.0 | 1.4±0.6 | 1.9±0.9 | 2.2±0.8 | 0.015 | 0.5<3 |  |  |
|  | ACD | 0.8±0.8 | 1.6±1.4 | 2.9±1.4 | 3.3±1.6 | 0.005 | 0.5<2 |  |  |
| Eating abnormalities | frequency | 0.8±1.6 | 1.3±1.8 | 1.3±1.9 | 1.2±1.8 | 0.700 |  |  |  |
|  | severity | 1.8±0.8 | 1.6±0.7 | 1.6±0.8 | 2.5±0.7 | 0.424 |  |  |  |
|  | ACD | 1.4±1.1 | 1.3±1.3 | 2.0±1.5 | 5.0±0.0 | 0.084 |  |  |  |

CDR: clinical dementia rating, ACD: associated caregiver distress, AMB: Aberrant motor behavior

^1^Comparison between 4 CDR groups, Kruskal-Wallis test

^2^Mann-Whitney U test (p <0.05/6=0.0083)
