# Supplementary material for: Differences of Behavioral and Psychological Symptoms of Dementia in Disease Severity in Four Major Dementias
Source: PLoS One. 2016 Aug 18;11(8):e0161092. doi: 10.1371/journal.pone.0161092 (PMC4990196; doi:10.1371/journal.pone.0161092)
Supplement: S10 Table — (DOCX) [file pone.0161092.s014.docx]

**S10 Table. Percentages of patients of individual domains according to dementia severity in patients with Vascular dementia**

|  |  | CDR | | | |
| --- | --- | --- | --- | --- | --- |
| Symptoms | patients with | 0.5 | 1 | 2 | 3 |
| Delusions | symptom | 15.0 | 13.6 | 25.8 | 50.0 |
|  | severity 2 or 3 | 66.6 | 60.0 | 75.0 | 83.3 |
|  | ACD^1^ | 80.0 | 60.0 | 75.0 | 75.0 |
| Hallucinations | symptom | 12.5 | 12.5 | 13.0 | 41.7 |
|  | severity 2 or 3 | 40.0 | 33.3 | 75.0 | 40.0 |
|  | ACD^1^ | 0.0 | 44.5 | 75.0 | 50.0 |
| Agitation | symptom | 25.0 | 37.0 | 54.8 | 66.7 |
|  | severity 2 or 3 | 70.0 | 51.8 | 52.9 | 75.0 |
|  | ACD^1^ | 37.5 | 57.7 | 76.4 | 66.6 |
| Depression | symptom | 27.5 | 27.4 | 32.3 | 41.7 |
|  | severity 2 or 3 | 36.4 | 35.0 | 40.0 | 20.0 |
|  | ACD^1^ | 18.2 | 40 | 70.0 | 60.0 |
| Anxiety | symptom | 22.5 | 26.4 | 25.8 | 33.3 |
|  | severity 2 or 3 | 33.3 | 15.8 | 25.0 | 25.0 |
|  | ACD^1^ | 11.1 | 26.3 | 87.5 | 50 |
| Euphoria | symptom | 7.5 | 6.8 | 3.2 | 8.3 |
|  | severity 2 or 3 | 33.3 | 20.0 | 100 | 100 |
|  | ACD^1^ | 33.3 | 0.0 | 100 | 100 |
| Apathy | symptom | 52.5 | 87.7 | 90.3 | 91.7 |
|  | severity 2 or 3 | 33.3 | 59.4 | 71.4 | 81.8 |
|  | ACD^1^ | 5.0 | 26.5 | 38.5 | 66.6 |
| Disinhibition | symptom | 17.5 | 23.5 | 32.3 | 41.6 |
|  | severity 2 or 3 | 42.9 | 41.2 | 70.0 | 100 |
|  | ACD^1^ | 42.9 | 62.4 | 40.0 | 50.0 |
| Irritability | symptom | 40.0 | 34.2 | 45.2 | 58.3 |
|  | severity 2 or 3 | 25.0 | 40.0 | 78.6 | 57.1 |
|  | ACD^1^ | 26.7 | 54.1 | 57.2 | 60.0 |
| AMB | symptom | 10.0 | 13.7 | 16.1 | 41.7 |
|  | severity 2 or 3 | 25.0 | 80.0 | 60.0 | 80.0 |
|  | ACD^1^ | 25.0 | 30.0 | 25.0 | 100 |
| Sleep disturbances | symptom | 25.0 | 31.4 | 63.6 | 85.7 |
|  | severity 2 or 3 | 0.0 | 31.3 | 57.2 | 83.3 |
|  | ACD^1^ | 0.0 | 18.8 | 64.3 | 66.6 |
| Eating abnormalities | symptom | 21.7 | 36.0 | 33.4 | 33.4 |
|  | severity 2 or 3 | 60.0 | 50.0 | 42.9 | 100 |
|  | ACD^1^ | 20.0 | 11.2 | 28.6 | 100 |

CDR: clinical dementia rating, ACD: associated caregiver distress, ^1^Moderate or greater distress, AMB: Aberrant motor behavior
