# Supplementary material for: Differences of Behavioral and Psychological Symptoms of Dementia in Disease Severity in Four Major Dementias
Source: PLoS One. 2016 Aug 18;11(8):e0161092. doi: 10.1371/journal.pone.0161092 (PMC4990196; doi:10.1371/journal.pone.0161092)
Supplement: S11 Table — (DOCX) [file pone.0161092.s015.docx]

**S11 Table. Factor loadings for BPSDs in patients with Frontotemporal lobar degeneration**

|  | Factor 1 | Factor 2 | Factor 3 | Factor 4 | Factor 5 |
| --- | --- | --- | --- | --- | --- |
| Eigenvalues | 3.98 | 1.65 | 1.35 | 1.15 | 1.04 |
| % of variance explained | 33.2 | 13.7 | 11.3 | 9.6 | 8.7 |
| Delusions | **0.366** | -0.039 | **0.619** | -0.236 | **0.371** |
| Hallucinations | -0.018 | **0.868** | 0.207 | 0.038 | -0.113 |
| Agitation | **0.757** | 0.292 | 0.297 | 0.120 | 0.135 |
| Depression | 0.018 | -0.001 | 0.028 | 0.116 | **0.937** |
| Anxiety | 0.050 | **0.699** | 0.210 | -0.093 | **0.414** |
| Euphoria | **0.463** | **0.599** | -0.124 | 0.253 | -0.117 |
| Apathy | 0.021 | -0.014 | 0.180 | **0.875** | 0.027 |
| Disinhibition | **0.864** | 0.121 | 0.074 | 0.217 | -0.063 |
| Irritability | **0.812** | -0.215 | 0.200 | -0.181 | 0.039 |
| AMB | 0.299 | 0.069 | **0.725** | **0.348** | 0.089 |
| Sleep disturbances | 0.019 | **0.339** | **0.815** | 0.164 | -0.110 |
| Eating abnormalities | **0.514** | 0.205 | 0.053 | **0.539** | 0.188 |

AMB: Aberrant motor behavior

Significant loadings (≥ 0.30) were entered into the factor and are displayed in boldface.

The value of KMO was 0.694, and the Barlett’s sphericity test reached statistical significance (χ²=262.5, df=66, p<0.001). The PCA found five components with eigenvalues exceeding 1, explaining 33.2, 13.7, 11.3, 9.6, and 8.7 percent of the variance respectively. Moreover, a plain break after the fifth component was seen by visual inspection of the scree plot. The Varimax rotation classified the 12 BPSDs into five factor.
