# Supplementary material for: Differences of Behavioral and Psychological Symptoms of Dementia in Disease Severity in Four Major Dementias
Source: PLoS One. 2016 Aug 18;11(8):e0161092. doi: 10.1371/journal.pone.0161092 (PMC4990196; doi:10.1371/journal.pone.0161092)
Supplement: S13 Table — (DOCX) [file pone.0161092.s017.docx]

**S13 Table. Percentages of patients of individual domains according to dementia severity in patients with Frontotemporal lobar degeneration**

|  |  | CDR | | | |
| --- | --- | --- | --- | --- | --- |
| Symptoms | patients with | 0.5 | 1 | 2 | 3 |
| Delusions | symptom | 2.9 | 11.4 | 20.0 | 28.6 |
|  | severity 2 or 3 | 0.0 | 50.0 | 40.0 | 0.0 |
|  | ACD^1^ | 0.0 | 66.7 | 0.0 | 0.0 |
| Hallucinations | symptom | 0.0 | 11.4 | 0.0 | 28.6 |
|  | severity 2 or 3 | - | 0.0 | - | 50.0 |
|  | ACD^1^ | - | 0.0 | - | 50.0 |
| Agitation | symptom | 37.1 | 51.4 | 60.0 | 28.6 |
|  | severity 2 or 3 | 15.4 | 38.9 | 60.0 | 50.0 |
|  | ACD^1^ | 27.3 | 40.0 | 53.9 | 50.0 |
| Depression | symptom | 28.6 | 31.4 | 16.0 | 0.0 |
|  | severity 2 or 3 | 20.0 | 18.2 | 25.0 | - |
|  | ACD^1^ | 22.2 | 12.5 | 25.0 | - |
| Anxiety | symptom | 22.9 | 28.6 | 48.0 | 28.6 |
|  | severity 2 or 3 | 12.5 | 20.0 | 16.6 | 100.0 |
|  | ACD^1^ | 0.0 | 14.3 | 10.0 | 50.0 |
| Euphoria | symptom | 11.4 | 20.0 | 32.0 | 14.3 |
|  | severity 2 or 3 | 25.0 | 14.3 | 37.5 | 100.0 |
|  | ACD^1^ | 0.0 | 25.0 | 0.0 | 0.0 |
| Apathy | symptom | 68.6 | 71.4 | 92.0 | 100.0 |
|  | severity 2 or 3 | 20.8 | 40.0 | 78.3 | 85.8 |
|  | ACD^1^ | 23.9 | 13.6 | 35.0 | 71.5 |
| Disinhibition | symptom | 34.3 | 48.6 | 60.0 | 14.3 |
|  | severity 2 or 3 | 25.0 | 47.0 | 53.3 | 100.0 |
|  | ACD^1^ | 11.1 | 42.9 | 74.9 | 0.0 |
| Irritability | symptom | 17.1 | 51.4 | 52.0 | 14.3 |
|  | severity 2 or 3 | 0.0 | 38.9 | 53.9 | 0.0 |
|  | ACD^1^ | 50.0 | 37.5 | 72.8 | 100 |
| AMB | symptom | 28.6 | 37.1 | 60.0 | 57.1 |
|  | severity 2 or 3 | 40.0 | 84.6 | 66.7 | 75.0 |
|  | ACD^1^ | 14.3 | 40.0 | 66.7 | 75.0 |
| Sleep disturbances | symptom | 23.1 | 20.0 | 57.1 | 80.0 |
|  | severity 2 or 3 | 0.0 | 50.0 | 58.3 | 75.0 |
|  | ACD^1^ | 0.0 | 25.0 | 58.3 | 50.0 |
| Eating abnormalities | symptom | 39.1 | 77.8 | 58.8 | 60.0 |
|  | severity 2 or 3 | 44.4 | 50.0 | 50.0 | 66.7 |
|  | ACD^1^ | 22.2 | 42.8 | 30.0 | 0.0 |

CDR: clinical dementia rating, ACD: associated caregiver distress, ^1^Moderate or greater distress AMB: Aberrant motor behavior
